# Computational and Experimental Studies on the α-Functionalization of Ketones Using Domino Reactions: A Strategy to Increase Chemoselectivity at the α-Carbon of Ketones

**DOI:** 10.3390/molecules30051114

**Published:** 2025-02-28

**Authors:** Hui Sun, Li-Heng Yang, Meng-Yun Fu, Bin Cui

**Affiliations:** Manganese Catalysis and Asymmetric Synthesis Laboratory, Hebei University of Science and Technology, Shijiazhuang 050018, China; 15944666234@163.com (L.-H.Y.); ff13403122702@126.com (M.-Y.F.)

**Keywords:** domino reactions, chemoselectivity, α-functionalization of carbonyl compounds, DFT calculation

## Abstract

A facile strategy to increase the chemoselectivity of domino reactions was proposed and successfully applied in the α-functionalization of ketones. The strategy involved widening the activation energy of the main reaction and side reaction through intermolecular interactions, thereby increasing the chemoselectivity of the domino reaction. In the proposed α-functionalization reaction, TMSCF_3_ acted as an excellent reagent which increased the nucleophilicity of DMF through the Van der Waals force and reduced the nucleophilicity of H_2_O through a hydrogen bond. We found that TMSCF_3_ can increase the activation energy difference between the main reaction and side reaction using DFT calculations, which greatly increased chemoselectivity and avoided the formation of by-products. TMSCF_3_ was recycled by rectification, and the average recovery rate was 87.2%. DFT calculations, XRD experiments, and control experiments were performed to support this mechanism. We are confident that this strategy has the potential to deliver significant practical advancements while simultaneously fostering broader innovation in the field of domino synthesis.

## 1. Introduction

**Scheme 1:**
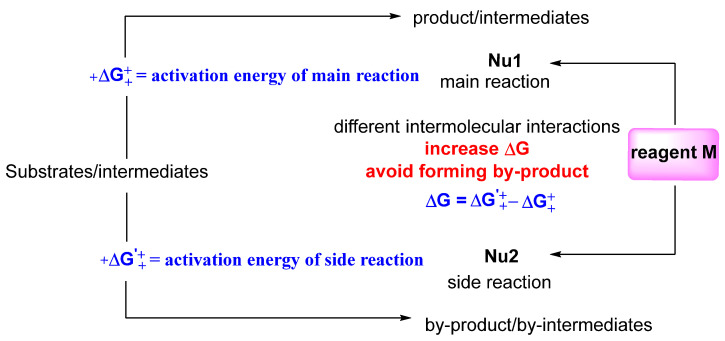
A facile strategy to increase the chemoselectivity of domino reactions.

Domino reactions incorporate two or more consecutive transformations in a single reaction vessel, in which the formed functionalities in the previous reaction steps are used in subsequent transformations without the addition of other materials [1,2,3,4,5]. Given that domino reactions proceed without the isolation of any intermediates, they have the advantages of atom-economy, reduced generation of waste, and the saving of materials, energy and time. Nevertheless, domino reactions often bring about side reactions [2,3,4,5,6,7]. The main reason for this is that domino reactions cannot guarantee that the product of the previous step will only be the next step without the inclusion of other steps within such complex reaction conditions. In order to solve the chemoselectivity problem of domino reactions, we conceived a facile strategy, as shown in Scheme 1. We added reagent **M** into the domino reaction system to expand the activation energy of the main reaction and side reaction through intermolecular interactions, thereby increasing chemoselectivity and yields (Scheme 1). Under the guidance of the proposed strategy, we carried out a series of explorations to prove the feasibility of the strategy. As a result, we successfully designed an α-functionalization method involving a carbonyl compound reaction via the bromination/nucleophilic substitution/hydrolysis sequence based on the above-mentioned strategy. We believe that this strategy has the potential to fundamentally reshape the landscape of multi-step synthetic operations, with the ability to convert reactions previously limited by low chemoselectivity into highly chemoselective domino transformations. Beyond simply enhancing chemoselectivity and improving main product yields, this strategy provides both a theoretical framework and practical case studies for conceiving and developing domino synthetic methodologies. In doing so, it not only delivers tangible advancements in application but also encourages broader innovation in domino synthesis.

**Scheme 2:**
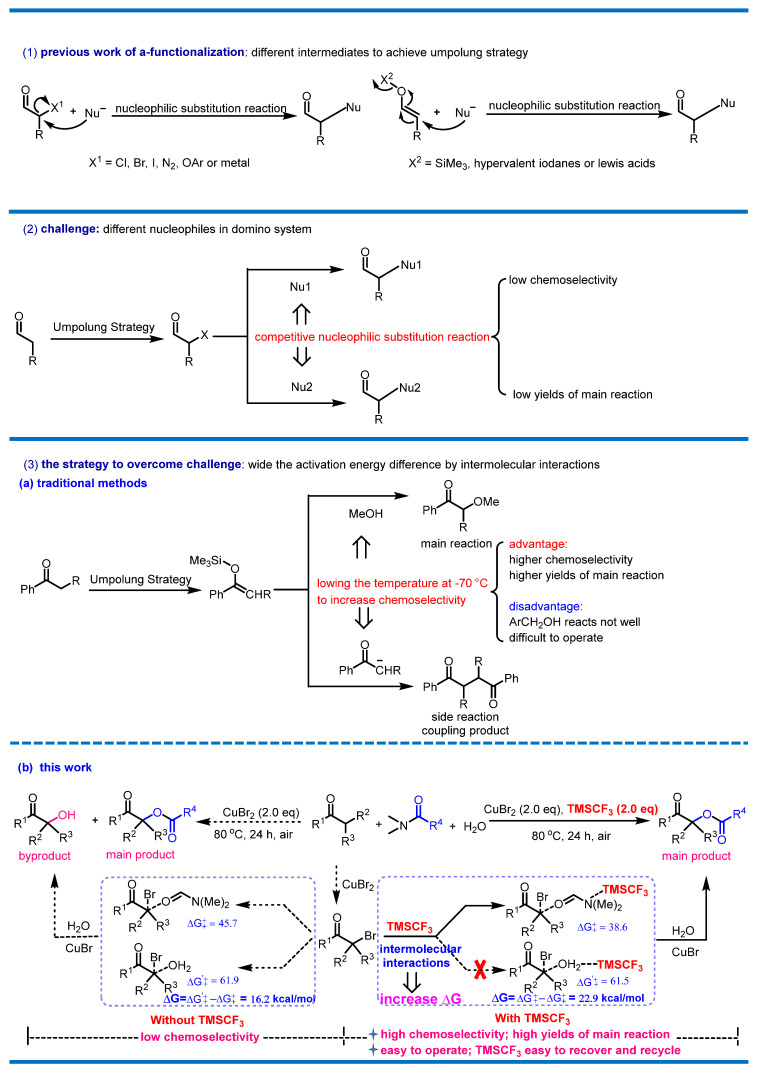
(**1**) Previous works on α-functionalization; (**2**) the challenge of two nucleophiles in a domino reaction; (**3**) (**a**) traditional methods; and (**b**) this work created to overcome the challenge.

**Scheme 3:**
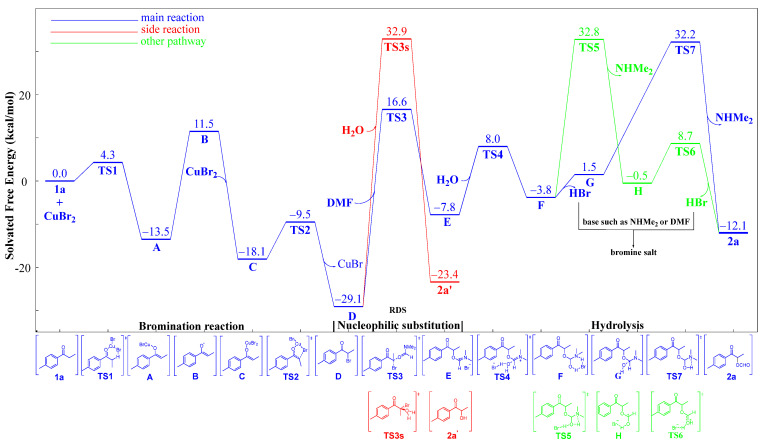
DFT calculations of domino strategy.

Many α-functionalization reactions of carbonyl compounds, which have been the subject of increasing interest in recent decades, are cascade reactions that require the reversal of the polarity of carbonyl compounds [8,9]. In conversion, functionalization at the α-position can be achieved with the help of various intermediates [10,11,12,13], such as halogens [14,15,16,17,18,19,20,21,22,23,24], hypervalent iodine [25,26,27,28,29], silyl enol ethers, ethers [30], α-diazo ketones [31,32,33,34], Lewis acids [35], and transition-metal catalysts (Scheme 2, (1)) [36,37]. These intermediates obtain the target compounds through the nucleophilic substitution process. Due to the presence of multiple nucleophiles in the domino reaction system, the nucleophilic substitution process may produce undesirable by-products, resulting in low chemoselectivity and yields (Scheme 2, (2)). In previous studies, many reactions needed to be achieved through the multi-step reaction process instead of the domino strategy in order to increase chemoselectivity [38,39,40,41,42,43]. Although many reactions were tolerated through the domino strategy, these studies required extreme measures to increase chemoselectivity, such as lowering the temperature [44,45], which can reduce reactivity and the reaction scope (Scheme 2, (3a)). Compared with these traditional strategies used to increase chemoselectivity, our proposed strategy does not sacrifice excellent reactivity and the reaction scope in exchange for relatively high chemoselectivity (Scheme 2, (3a)). Therefore, we applied the proposed strategy to the α-functionalization of ketones in order to increase chemoselectivity without affecting its reactivity and the reaction scope (Scheme 2, (3b)).

**Figure 1:**
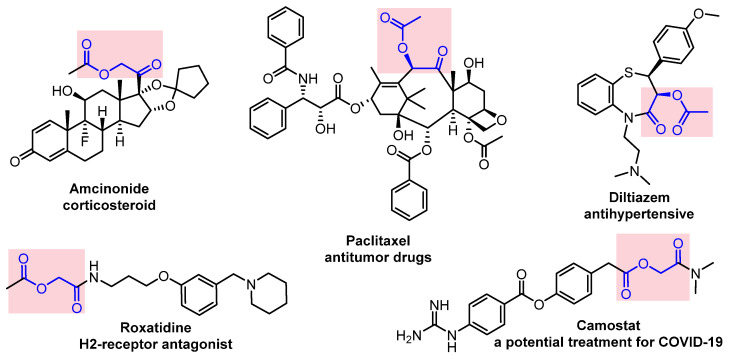
Drugs containing α-acyloxycarbonyl group.

As seen in the above-mentioned works regarding the α-functionalization of ketones, α-acyloxycarbonyl compounds are versatile synthons for the synthesis of various cyclic and heterocyclic natural products and pharmaceuticals [46]. A number of classic drugs, lead compounds, anti-inflammatory drugs [47], antitumor drugs, antihypertensive drugs, H2 receptor antagonists, and potential treatment drugs for COVID-19 [48,49,50] contain α-acyloxycarbonyl group (Figure 1). The development of a green and practical methodology using the domino strategy is needed for the preparation of α-acyloxy ketones.

## 2. Results and Discussion

In order to increase the chemoselectivity of the reaction and reduce the yield of by-products, the mechanism of the acyloxylation domino reaction was studied by DFT calculations (Scheme 3). Computational and experimental studies showed that nucleophilic substitution is the main process for the production of by-product **2a′** (Scheme 3, nucleophilic substitution). The reason for this is that the domino system contained both H_2_O and DMF nucleophiles, and the Gibbs free energy difference of TS3 and TS3s was 16.3 kcal/mol, which caused the side reaction to proceed under 80 °C. In order to improve the chemoselectivity of the reaction, we explored a suitable method to reduce the Gibbs free energy of TS3 or to increase the Gibbs free energy of TS3s, thereby making the barrier larger between the Gibbs free energy of TS3 and TS3s.

**Figure 2:**
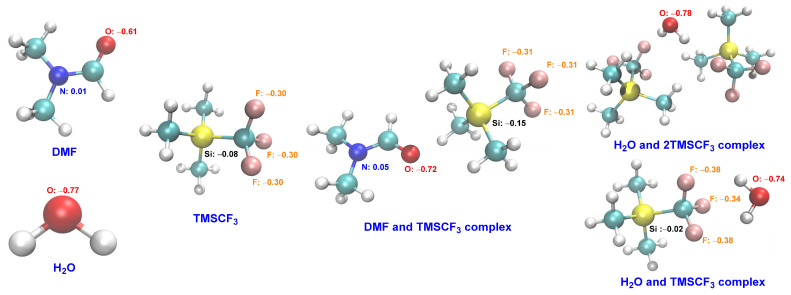
The charge distribution of DMF, H_2_O, TMSCF_3,_ and the complex.

In our previous work, we found that DMF and TMSCF_3_ reagents interact together and to successfully activate trifluoromethyl [51,52]. The ^1^H NMR experiments were carried out to study the possible interaction between H_2_O and TMSCF_3_. Upfield shifts were observed for protons of H_2_O when H_2_O and TMSCF_3_ were mixed in CDCl_3_ (Table 1). Our previous work and ^1^H NMR experiments indicated that an interaction existed between TMSCF_3_, H_2_O, and DMF. When using the DFT calculation for the DMF and TMSCF_3_ complex, we found that the oxygen’s charge [53,54,55] of the DMF and TMSCF_3_ complex was increased compared with that of DMF (Figure 2, the DMF and TMSCF_3_ complex vs. DMF). The oxygen’s charge [53,54,55] of the H_2_O and TMSCF_3_ complex, which was formed through hydrogen bonding, was slightly reduced compared with that of H_2_O (Figure 2, the H_2_O and TMSCF_3_ complex vs. H_2_O). Similarly, the condensed Fukui functions and RDG proved that the results are consistent with the charge analysis method (the details of this are in the Supplementary Materials: Part 2. the condensed Fukui functions and RDG of DMF, H_2_O, and their complexes with TMSCF_3_). These results laterally showed that the addition of TMSCF_3_ increases the nucleophilicity of DMF and reduces the nucleophilicity of H_2_O.

**Scheme 4:**
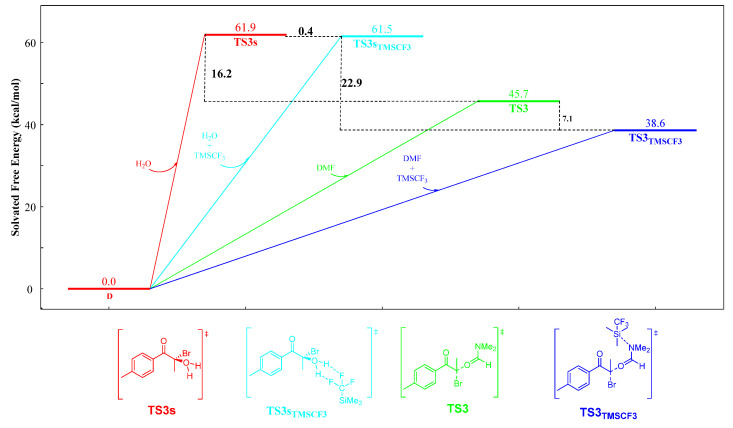
The activation energy of TS3, TS3s, TS3_TMSCF3,_ and TS3s_TMSCF3_.

In order to further prove the rationality of the strategy, we calculated the nucleophilic substitution of the domino reaction under TMSCF_3_ conditions, and the results are shown in Scheme 4. The results show that the activation energy decreased after adding TMSCF_3_ (Scheme 4, **TS3_TMSCF3_** vs. **TS3** and **TS3s_TMSCF3_** vs. **TS3s**), and the Gibbs free energy increased to 22.9 kcal/mol (Scheme 4, **TS3_TMSCF3_** vs. **TS3s_TMSCF3_**). In the process of the nucleophilic substitution reaction, DMF had a greater advantage and it was difficult or impossible to react H_2_O because of its excessively high activation energy (Scheme 4, **TS3_TMSCF3_** vs. **TS3s_TMSCF3_**). Therefore, this strategy can prevent the formation of by-products and can increase chemoselectivity. The enhancement of the chemoselectivity of the nucleophilic substitution process was beneficial for the subsequent hydrolysis process, which required the participation of H_2_O (Scheme 3, hydrolysis).

On the basis of the DFT calculation results, we surveyed a variety of reaction conditions using **1a** and DMF as a model system, as seen in Table 2. We found that CuBr_2_ effectively participates in the required reaction of **1a** and DMF to provide **2a** under TMSCF_3_ conditions, and the by-product **2a′** was not detected (Table 2, entry 1). An amount of 65% of the yield of **2a** and 23% of the yield of by-product **2a′** was obtained without TMSCF_3,_ which is consistent with the result of the DFT calculations (Table 2, entry 2). The results were unsatisfactory when other trimethylsilyl derivatives were used (Table S1 in Supplementary Materials). Other halogenated copper salts were used instead of CuBr_2,_ and the result was not satisfactory (Table 2, entries 3–6). Reactions without CuBr_2_ were not successful (Table 2, entry 7). A further reduction in the amount of CuBr_2_ from 2.0 equiv to 1.0 equiv led to a drop in substrate conversions (Table 2, entries 1 vs. 8) and the increase in the amount of CuBr_2_ from 2.0 equiv to 3.0 equiv had little effect on the substrate conversions (Table 2, entries 1 vs. 9). The reaction was suppressed without H_2_O (Table 2, entry 10) and the reaction had little effect on the Ar protection condition compared with the air condition (Table 2, entry 11). Therefore, the α-formyloxylation reaction was finally carried out in the standard conditions.

**Figure 3:**
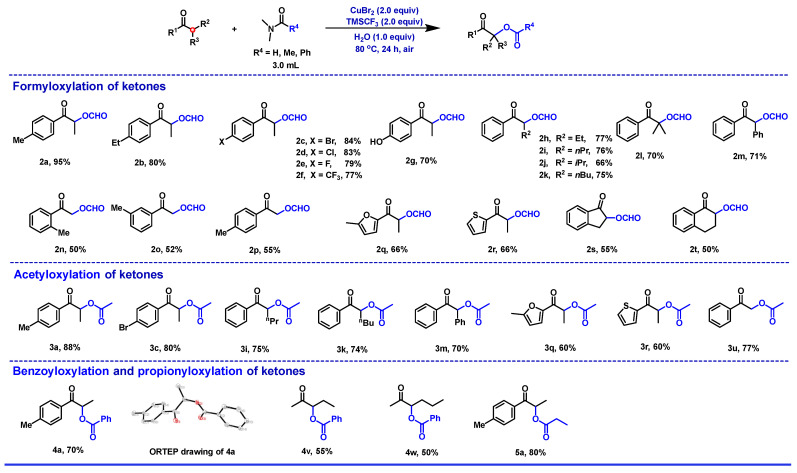
α-Formyloxylation and α-acyloxylation of ketone substrates.

Having identified the optimal reaction conditions, various substituted ketones were evaluated to determine their influence on the overall reactivity and to establish the reaction scope (Figure 3). In general, the α-formyloxylation methodology was amenable to a range of aryl-substituted α-formyloxy ketones that bear various electron-withdrawing and electron-donating substituents, furnishing the corresponding products in typically good-to-excellent yields (Figure 3, **2a**–**2g**). The substrates of *ortho*-methyl (**1n**), *meta*-methyl (**1o**), and *para*-methyl (**1p**) substituents on the benzene rings did not significantly influence the reaction process. The steric effect of aromatic and aliphatic ketones was investigated and the results indicate that the small groups were slightly superior to the bulky groups in the reactions (Figure 3, **2h**–**2m**). Other aromatic rings of the aromatic ketones, such as the furan ring and the thiophene ring, had little effect on the course of the reaction, and the resulting products were obtained in good isolated yields (Figure 3, **2q**–**2r**). Cyclic ketones such as **1s** and **1t** obtained corresponding products in moderate yields. To prove the scalability of the reaction, substrate **1a** was carried out on a gram scale, and the desired product **2a** was obtained in a 63% isolated yield. By slowly adding CuBr_2_ without changing the amounts of other materials, the yield increased to 92%. After the α-formyloxylation of the ketones, the α-acyloxylation of the ketones was included within the reaction with N,N-dimethylamides (R^4^ = Me, Ph, and Et) instead of DMF (Figure 3, **3**, **4** and **5**). N,N-dimethylamides (R^4^ = Me, Ph, and Et) displayed good reactivity under standard conditions, and the results indicated that the size of the acyl group on the N,N-dimethylamides had little effect on the course of the reaction (Figure 3, **2**, **3**, **4,** and **5**). Compound **4a** was subjected to an X-ray diffraction experiment to determine the skeleton, and the ORTEP drawing clearly confirmed the structure (Figure S2 in the Supplementary Materials).

**Scheme 5:**
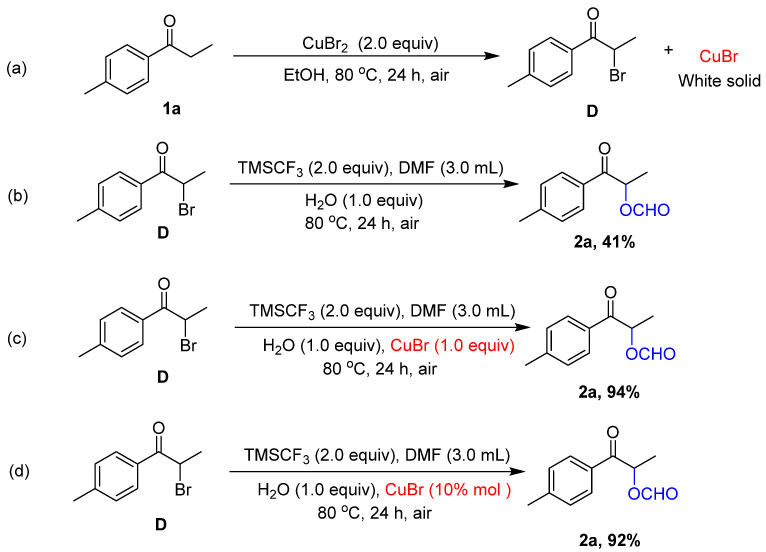
Control experiments of domino reaction processes.

Although DFT calculations had theoretically provided the domino reaction mechanism (Scheme 3), it was necessary to further verify the domino reaction process through experiments. The designed domino reaction had three reaction processes, namely bromination, nucleophilic substitution, and hydrolysis. We adopted a segmented method to verify the reaction process in Scheme 5. To ensure that the reaction first took place through the bromination reaction process, we added **1a**, CuBr_2,_ without any other substances to the reaction system. The brominated product **D** and a white solid powder were obtained after careful separation and purification (Scheme 5, (a)). Based on the designed reaction mechanism and the results of DFT calculations, we guessed that the white solid powder was CuBr. Further XRD studies of the white solid powder indicated that it was CuBr (Figure S1 in the Supplementary Materials). It was proven that the bromination reaction process was tolerated because of the designed mechanism. Then, we added **D**, DMF, TMSCF_3,_ and H_2_O to the reaction system, which performed nucleophilic substitution and hydrolysis processes. We found that the reaction was achieved, but the yield of **2a** was significantly lower than that in the domino reaction system (Scheme 5, (b)). We guessed that the product CuBr of the previous bromination process promoted the subsequent processes. CuBr was added to the reaction system of (b), and we found that it greatly promoted the processes of nucleophilic substitution and hydrolysis (Scheme 5, (b) vs. (c)). It was noteworthy that CuBr displayed good reactivity, and 10% CuBr was shown to be successful (Scheme 5, (d)).

**Scheme 6:**
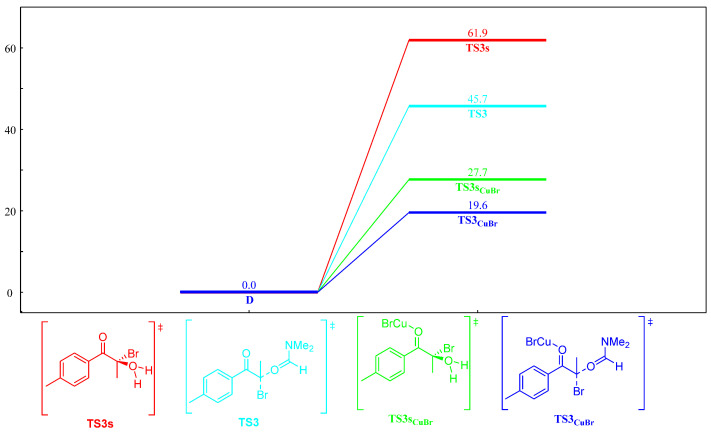
The activation energy of **TS3**, **TS3s**, **TS3_CuBr_**, and **TS3s_CuBr_**.

To obtain detailed insight into the role of CuBr in the domino reaction, control experiments and DFT calculations were carried out, as seen in Scheme 6. CuBr greatly reduced the activation energy of the nucleophilic substitution process (Scheme 6, **TS3_CuBr_** vs. **TS3** and **TS3s_CuBr_** vs. **TS3s**). However, CuBr had little effect on the activation energy difference between the main reaction and the side reaction (Scheme 6, the difference between **TS3** and **TS3s** vs. the difference between **TS3_CuBr_** and **TS3s_CuBr_**), which did not increase chemoselectivity.

TMSCF_3_ played a key role in increasing the chemoselectivity of the reaction. We successfully recovered TMSCF_3_ in the reaction system through rectification (Figure S3 in the Supplementary Materials). This showed that TMSCF_3_ could be recovered and reused, which embodies the concept of atomic economy.

## 3. Conclusions

In summary, a facile strategy was proposed to increase the chemoselectivity of domino reactions, and this strategy was used to design an α-formyloxylation and an α-acyloxylation domino reaction of ketones. The α-acyloxylation of ketones proved the feasibility of the strategy. TMSCF_3,_ when added to the reaction system, enlarged the activation energy difference between the main reaction and side reaction through intermolecular interactions, thereby increasing chemoselectivity and avoiding the formation of by-products. TMSCF_3_ was recovered and reused, which embodies the concept of atomic economy. The chemoselectivity problem of other multi-step reactions or domino reactions solved by this strategy is under further study.

## 4. Materials and Methods

### 4.1. General Experimental Information

All reactions were carried out in sealed tube at 80 °C under air, unless stated otherwise. The purchased reagents were of the highest commercial quality and were used without further purification, unless otherwise stated. DMF and DMA were dried by CaCl_2_ and vigorously stirred with CaH_2_ overnight; then, this mixture was distilled out and sealed using 4 Å molecular sieves for storage under N_2_ protection. The reagents were weighed using a Sartorius analytical balance (BCE224-1CCN) with a precision of 0.0001 g. The ^1^H and ^13^C NMR spectra were recorded on a Bruker DRX 500 (or 400) spectrometer at 298 K using deuterated chloroform as the solvent and TMS as the internal reference. The ^1^H NMR were recorded as follows: chemical shift (δ, ppm), multiplicity (s = singlet, d = doublet, t = triplet, m = multiplet, coupling constant (s) in Hz, integration). The data for ^13^C NMR are reported in terms of chemical shift (δ, ppm). The reactions were monitored through thin layer chromatography (TLC) on silica gel plates (GF 254) using a mixture of iodine and silica gel as the visualizing agent, unless otherwise stated. Flash column chromatography was performed using silica gel (200–400 meshes). The infrared (IR) spectra were recorded with a KBr pellet, and the wavenumbers were given in cm^−1^. HRMS analyses were carried out with Varian FTICR-MS 7.0 T. The melting points were obtained on a Mettler Toledo MP50 apparatus.

### 4.2. General Procedure for Acyloxylation Reaction

Ketone (1.0 mmol), CuBr_2_ (2.0 mmol, 446.7 mg), TMSCF_3_ (2.0 mmol, 284.4 mg), and H_2_O (1.0 mmol, 18.0 mg) were added to a sealed tube (40 mL). Amide (3.0 mL) was added, and the mixture was stirred vigorously at 80 °C for 24 h. After completion, the reaction mixture was added into DCM (30 mL) and washed with NaCl solution (50 mL × 3). The organic mixture was dried with MgSO_4_, concentrated in vacuo, and separated with flash column chromatography.

### 4.3. Computational Details

All DFT [56] calculations were carried out using the Gaussian 16 ES64L-G16RevB.01 [57] suite of programs, and some calculated results were processed using Multiwfn [54,55]. The geometries of intermediates and transition states were optimized using the PBE0 [58,59,60] level of theory in combination with the DFT-D3 dispersion corrections with the Becke–Johnson damping scheme (D3BJ) [61,62]. The def2-SVP basis set [63,64] was used for all atoms and the solvent effects were computed with the SMD solvation model [65] (solvent = DMF). Vibrational frequency calculations were performed for all stationary points to confirm if each optimized structure was a local minimum or a transition state structure. All of the optimized transition state structures had only one imaginary (negative) frequency, and all minima (reactants, products, and intermediates) had no imaginary frequencies. The single-point energies and solvent effects were computed with the wB97XD [66]/def2-TZVPP [67,68] basis sets. The corrections of free energies [69] were applied for entropy calculations with a frequency cut-off of 100 cm^−1^ using the Shermo [70] program. The relative Gibbs free energies are given in kcal/mol, which were calculated by adding the gas-phase thermal and non-thermal corrections at 353.15 K to the single-point energies. The charge calculations were conducted at the B3LYP/6–31G + (d,p) level through becke charge [53]. The 3D images of the structures were prepared using VMD [71].

### 4.4. General Procedure for Control Experiments Processes

#### 4.4.1. CuBr_2_ Without Other Substances Supported the Reaction

**1a** (1.0 mmol, 148.5 mg) and CuBr_2_ (2.0 mmol, 446.74 mg) were added to a sealed tube (40 mL). EtOH (3.0 mL) was added, and the mixture was stirred vigorously at 80 °C for 24 h. After completion, the reaction mixture was filtered. Then, the white powder and organic filtrate were obtained. XRD studies of the white powder indicated that it was CuBr (Figure S1). The organic filtrate was added into DCM (30 mL) and washed with NaCl solution (50 mL × 3). The organic mixture was dried with MgSO_4_, concentrated in vacuo, and separated with flash column chromatography (petroleum ether:ethyl acetate = 60:1) to obtain **D.** ^1^H NMR (500 MHz, CDCl_3_) δ 7.92 (d, *J* = 8.2 Hz, 2H), 7.28 (d, *J* = 8.0 Hz, 2H), 5.28 (q, *J* = 6.6 Hz, 1H), 2.42 (s, 3H), 1.89 (d, *J* = 6.6 Hz, 3H). ^13^C NMR (126 MHz, CDCl_3_) δ 193.0, 144.7, 131.5, 129.5, 129.1, 41.7, 21.7, 20.2.

#### 4.4.2. The Reaction Was Supported Without CuBr

**D** (1.0 mmol), TMSCF_3_ (2.0 mmol, 284.4 mg), and H_2_O (1.0 mmol, 18.0 mg) were added to a sealed tube (40 mL). DMF (3.0 mL) was added, and the mixture was stirred vigorously at 80 °C for 24 h. After completion, the reaction mixture was added into DCM (30 mL) and washed with NaCl solution (50 mL × 3). The organic mixture was dried with MgSO_4_, concentrated in vacuo, and was separated with flash column chromatography to obtain **2a** (41%).

#### 4.4.3. CuBr Supported the Reaction

**D** (1.0 mmol), CuBr (1.0 mmol, 143.5 mg), TMSCF_3_ (2.0 mmol, 284.4 mg), and H_2_O (1.0 mmol, 18.0 mg) were added to a sealed tube (40 mL). DMF (3.0 mL) was added, and the mixture was stirred vigorously at 80 °C for 24 h. After completion, the reaction mixture was added into DCM (30 mL) and washed with NaCl solution (50 mL × 3). The organic mixture was dried with MgSO_4_, concentrated in vacuo, and separated with flash column chromatography to obtain **2a** (94%).

#### 4.4.4. A 10% Amount of CuBr Supported the Reaction

**D** (1.0 mmol), CuBr (1.0 mmol, 143.5 mg), TMSCF_3_ (2.0 mmol, 284.4 mg), and H_2_O (1.0 mmol, 18.0 mg) were added to a sealed tube (40 mL). DMF (3.0 mL) was added and the mixture was stirred vigorously at 80 °C for 24 h. After completion, the reaction mixture was added into DCM (30 mL) and washed with NaCl solution (50 mL × 3). The organic mixture was dried with MgSO_4_, concentrated in vacuo, and separated with flash column chromatography to obtain **2a** (92%).

#### 4.4.5. Scale-Up Experiments

**1a** (10.0 mmol, 1.4850 g), CuBr_2_ (20.0 mmol, 4.4670 g), TMSCF_3_ (20.0 mmol, 2.8440 g), and H_2_O (10.0 mmol, 0.1800 g) were added to a sealed tube (100 mL). DMF (30.0 mL) was added, and the mixture was stirred vigorously at 80 °C for 24 h. After completion, the reaction mixture was added into DCM (300 mL) and washed with NaCl solution (500 mL × 3). The organic mixture was dried with MgSO_4_, concentrated in vacuo, and separated with flash column chromatography to obtain **2a** (63%). When the operation was changed by slowly adding CuBr, the yield of **2a** increased to 92%.

### 4.5. General Procedure for Rectification

The α-Acyloxylation reaction of substrate **1a** was carried out on a gram scale (20.2 g) under the optimized reaction conditions with TMSCF_3_ (28.4 g). After completion, the reaction mixture was added into a rectification tower, and then TMSCF_3_ was recovered (Figure S3).

### 4.6. Compounds Characterization

#### 4.6.1. 1-Oxo-1-(p-tolyl)propan-2-yl Formate (**2a**)

According to the general procedure, **1a** (1.0 mmol, 148.5 mg), CuBr_2_ (2.0 mmol, 446.7 mg), TMSCF_3_ (2.0 mmol, 284.4 mg), and H_2_O (1.0 mmol, 18.0 mg) were added to DMF (3.0 mL) with a reaction time of 24 h to produce **2a** (183.6 mg, 95% yield) which was isolated as a white solid powder (m.p. = 74–75 °C) after purification through silica gel chromatography (petroleum ether:ethyl acetate = 50:1). ^1^H NMR (500 MHz, CDCl_3_) δ 8.12 (s, 1H), 7.85 (d, *J* = 7.8 Hz, 2H), 7.29 (d, *J* = 7.9 Hz, 2H), 6.10 (q, *J* = 7.0 Hz, 1H), 2.42 (s, 3H), 1.57 (d, *J* = 7.0 Hz, 3H). ^13^C NMR (126 MHz, CDCl_3_) δ 195.3, 160.0, 144.7, 131.6, 129.6, 129.0, 70.6, 21.7, 17.2. IR (KBr): 2987, 2961, 2360, 2331, 1719, 1680, 1605, 1568, 1447, 1177, 1129 cm^−1^. HRMS-ESI (*m*/*z*): [M + H]^+^ calcd for C_11_H_12_O_3_, 193.0865; found: 193.0868.

#### 4.6.2. 1-(4-Ethylphenyl)-1-oxopropan-2-yl Formate (**2b**)

According to the general procedure, **1b** (1.0 mmol, 162.1 mg), CuBr_2_ (2.0 mmol, 446.7 mg), TMSCF_3_ (2.0 mmol, 284.4 mg), and H_2_O (1.0 mmol, 18.0 mg) were added to DMF (3.0 mL) to produce **2b** (165.4 mg, 80% yield), which was isolated as a colorless oil after purification through silica gel chromatography (petroleum ether:ethyl acetate = 90:1). ^1^H NMR (500 MHz, CDCl_3_) δ 8.12 (s, 1H), 7.88 (d, *J* = 8.3 Hz, 2H), 7.31 (d, *J* = 8.1 Hz, 2H), 6.11 (q, *J* = 7.0 Hz, 1H), 2.71 (q, *J* = 7.6 Hz, 2H), 1.57 (d, *J* = 7.0 Hz, 3H), 1.26 (t, *J* = 7.6 Hz, 3H). ^13^C NMR (126 MHz, CDCl_3_) δ 195.4, 160.1, 151.0, 131.8, 128.8, 128.4, 71.0, 29.0, 17.4, 15.1. IR (KBr): 3480, 3033, 2969, 2359, 1728, 1694, 1606, 1175, 971 cm^−1^. HRMS-ESI (*m*/*z*): [M + H]^+^ calcd for C_12_H_14_O_3_, 207.1021; found: 207.1024.

#### 4.6.3. 1-(4-Bromophenyl)-1-oxopropan-2-yl Formate (**2c**)

According to the general procedure, **1c** (1.0 mmol, 212.0 mg), CuBr_2_ (2.0 mmol, 446.7 mg), TMSCF_3_ (2.0 mmol, 284.4 mg), and H_2_O (1.0 mmol, 18.0 mg) were added to DMF (3.0 mL) to produce **2c** (215.1 mg, 84% yield), which was isolated as a colorless oil after purification through silica gel chromatography (petroleum ether:ethyl acetate = 130:1). ^1^H NMR (500 MHz, CDCl_3_) δ 8.12 (s, 1H), 7.89 (d, *J* = 8.6 Hz, 2H), 7.47 (d, *J* = 8.6 Hz, 2H), 6.04 (q, *J* = 7.0 Hz, 1H), 1.57 (d, *J* = 7.0 Hz, 3H). ^13^C NMR (126 MHz, CDCl_3_) δ 194.8, 160.0, 140.3, 132.4, 129.9, 129.2, 70.9, 17.1. IR (KBr): 3486, 3091, 2988, 2937, 1725, 1696, 1585, 1567, 1446, 1170 cm^−1^. The NMR data were in agreement with the reported results [72].

#### 4.6.4. 1-(4-Chlorophenyl)-1-oxopropan-2-yl Formate (**2d**)

According to the general procedure, **1d** (1.0 mmol, 168.0 mg), CuBr_2_ (2.0 mmol, 446.7 mg), TMSCF_3_ (2.0 mmol, 284.4 mg), and H_2_O (1.0 mmol, 18.0 mg) were added to DMF (3.0 mL) to produce **2d** (175.0 mg, 83% yield), which was isolated as a colorless oil after purification through silica gel chromatography (petroleum ether:ethyl acetate = 160:1). ^1^H NMR (500 MHz, CDCl_3_) δ 8.12 (s, 1H), 7.89 (d, *J* = 8.6 Hz, 2H), 7.47 (d, *J* = 8.6 Hz, 2H), 6.04 (q, *J* = 7.0 Hz, 1H), 1.57 (d, *J* = 7.0 Hz, 3H). ^13^C NMR (126 MHz, CDCl_3_) δ 193.9, 159.0, 131.8, 131.2, 128.9, 128.0, 69.8, 16.1. IR (KBr): 3093, 3067, 2992, 2939, 2362, 1723, 1696, 1588, 1165, 1091 cm^−1^. HRMS-ESI (*m*/*z*): [M + H]^+^ calcd for C_10_H_9_ClO_3_, 213.0318; found: 213.0318.

#### 4.6.5. 1-(4-Fluorophenyl)-1-oxopropan-2-yl Formate (**2e**)

According to the general procedure, **1e** (1.0 mmol,152.1 mg), CuBr_2_ (2.0 mmol, 446.7 mg), TMSCF_3_ (2.0 mmol, 284.4 mg), and H_2_O (1.0 mmol, 18.0 mg) were added to DMF (3.0 mL) to produce **2e** (154.3 mg, 79% yield), which was isolated as a colorless oil after purification through silica gel chromatography (petroleum ether:ethyl acetate = 100:1). ^1^H NMR (500 MHz, CDCl_3_) δ 8.12 (s, 1H), 8.05–7.95 (m, 2H), 7.17 (t, *J* = 8.6 Hz, 2H), 6.06 (q, *J* = 7.0 Hz, 1H), 1.58 (d, *J* = 7.0 Hz, 3H). ^13^C NMR (126 MHz, CDCl_3_) δ 194.3, 166.1(d,*^1^J*(C,F) = 252.0 Hz), 160.0, 131.2 (d, *^3^J*(C,F) = 12.6 Hz), 130.5 (d, *^4^J*(C,F) = 3.0 Hz), 116.1 (d, *^2^J*(C,F) = 25.2 Hz), 70.9, 17.2. IR (KBr): 3077, 2992, 2733, 2457, 1727, 1599, 1508, 1230, 1160, 971 cm^−1^. HRMS-ESI (*m*/*z*): [M + H]^+^ calcd for C_10_H_9_FO_3_, 197.0614; found: 197.0618.

#### 4.6.6. 1-(4-(Trifluoromethyl)phenyl)-1-oxopropan-2-yl Formate (**2f**)

According to the general procedure, **1f** (1.0 mmol, 202.1 mg), CuBr_2_ (2.0 mmol, 446.7 mg), TMSCF_3_ (2.0 mmol, 284.4 mg), and H_2_O (1.0 mmol, 18.0 mg) were added to DMF (3.0 mL) to produce **2f** (190.5 mg, 77% yield), which was isolated as a colorless oil after purification through silica gel chromatography (petroleum ether:ethyl acetate = 100:1). ^1^H NMR (500 MHz, CDCl_3_) δ 8.12 (s, 1H), 8.05 (d, *J* = 8.1 Hz, 2H), 7.77 (d, *J* = 8.1 Hz, 2H), 6.07 (q, *J* = 7.0 Hz, 1H), 1.59 (d, *J* = 7.0 Hz, 3H). ^13^C NMR (126 MHz, CDCl_3_) δ 195.2, 160.0, 137.0, 135.0 (q, *^2^J*(C,F) = 25.2 Hz), 128.8, 125.9 (q, *^3^J*(C,F) = 12.6 Hz), 123.4 (q, *^1^J*(C,F) = 277.2 Hz), 71.1, 16.9. IR (KBr): 3503, 2994, 2943, 2360, 1730, 1619, 1580, 1412, 1325, 1169 cm^−1^. HRMS-ESI (*m*/*z*): [M + H]^+^ calcd for C_11_H_9_F_3_O_3_, 247.0582; found: 247.0584.

#### 4.6.7. 1-(4-Hydroxyphenyl)-1-oxopropan-2-yl Formate (**2g**)

According to the general procedure, **1g** (1.0 mmol, 150.2 mg), CuBr_2_ (2.0 mmol, 446.7 mg), TMSCF_3_ (2.0 mmol, 284.4 mg), and H_2_O (1.0 mmol, 18.0 mg) were added to DMF (3.0 mL) to produce **2g** (136.5 mg, 70% yield), which was isolated as a white solid powder (m.p. = 66–67 °C) after purification through silica gel chromatography (petroleum ether:ethyl acetate = 190:1). ^1^H NMR (400 MHz, CDCl_3_) δ 8.15 (s, 1H), 8.03 (s, 1H), 7.75 (d, *J* = 8.8 Hz, 2H), 6.80 (d, *J* = 8.8 Hz, 2H), 5.99 (q, *J* = 7.0 Hz, 1H), 1.48 (d, *J* = 7.1 Hz, 3H). ^13^C NMR (101 MHz, CDCl_3_) δ 194.6, 161.1, 159.8, 130.4, 124.8, 114.9, 70.1, 16.6. IR (KBr): 3336, 2917, 2360, 2342, 1683, 1606, 1203, 1017, 853 cm^−1^. HRMS-ESI (*m*/*z*): [M + H]^+^ calcd for C_10_H_10_O_4_, 195.0657; found: 195.0655.

#### 4.6.8. 1-Oxo-1-phenylbutan-2-yl Formate (**2h**)

According to the general procedure, **1h** (1.0 mmol, 148.1 mg), CuBr_2_ (2.0 mmol, 446.7 mg), TMSCF_3_ (2.0 mmol, 284.4 mg), and H_2_O (1.0 mmol, 18.0 mg) were added to DMF (3.0 mL) to produce **2h** (147.5 mg, 77% yield), which was isolated as a colorless oil after purification through silica gel chromatography (petroleum ether:ethyl acetate = 60:1). ^1^H NMR (500 MHz, CDCl_3_) δ 8.17 (s, 1H), 7.94 (d, *J* = 7.3 Hz, 2H), 7.60 (t, *J* = 7.4 Hz, 1H), 7.48 (t, *J* = 7.7 Hz, 2H), 5.98 (dd, *J* = 7.9, 4.2 Hz, 1H), 2.07–1.84 (m, 2H), 1.03 (t, *J* = 7.4 Hz, 3H). ^13^C NMR (126 MHz, CDCl_3_) δ 195.5, 160.3, 134.6, 133.7, 128.9, 128.4, 75.9, 24.8, 9.7. IR (KBr): 3064, 2975, 2939, 2359, 2342, 1730, 1697, 1597, 1580, 1449, 1223, 1174 cm^−1^. HRMS-ESI (*m*/*z*): [M + H]^+^ calcd for C_11_H_12_O_3_, 193.0865; found: 193.0865.

#### 4.6.9. 1-Oxo-1-phenylpentan-2-yl Formate (**2i**)

According to the general procedure, **1i** (1.0 mmol, 162.1 mg), CuBr_2_ (2.0 mmol, 446.7 mg), TMSCF_3_ (2.0 mmol, 284.4 mg), and H_2_O (1.0 mmol, 18.0 mg) were added to DMF (3.0 mL) to produce **2i** (156.4 mg, 76% yield), which was isolated as a colorless oil after purification through silica gel chromatography (petroleum ether:ethyl acetate = 65:1). ^1^H NMR (500 MHz, CDCl_3_) δ 8.16 (s, 1H), 7.95 (d, *J* = 7.3 Hz, 2H), 7.60 (t, *J* = 7.4 Hz, 1H), 7.49 (t, *J* = 7.7 Hz, 2H), 6.03 (dd, *J* = 7.5, 5.3 Hz, 1H), 1.91–1.84 (m, 2H), 1.57–1.43 (m, 2H), 0.95 (t, *J* = 7.4 Hz, 3H). ^13^C NMR (126 MHz, CDCl_3_) δ 195.7, 160.3, 134.5, 133.7, 128.9, 128.4, 74.7, 33.4, 18.7, 13.6. IR (KBr): 3063, 2963, 2936, 2341, 1730, 1696, 1597, 1580, 1449, 1173 cm^−1^. HRMS-ESI (*m*/*z*): [M + H]^+^ calcd for C_12_H_14_O_3_, 207.1021; found: 207.1022.

#### 4.6.10. 3-Methyl-1-oxo-1-phenylbutan-2-yl Formate (**2j**)

According to the general procedure, **1j** (1.0 mmol, 162.1 mg), CuBr_2_ (2.0 mmol, 446.7 mg), TMSCF_3_ (2.0 mmol, 284.4 mg), and H_2_O (1.0 mmol, 18.0 mg) were added to DMF (3.0 mL) to produce **2j** (136.0 mg, 66% yield), which was isolated as a colorless oil after purification through silica gel chromatography (petroleum ether:ethyl acetate = 90:1). ^1^H NMR (500 MHz, CDCl_3_) δ 8.20 (s, 1H), 7.95 (d, *J* = 7.4 Hz, 2H), 7.60 (t, *J* = 7.4 Hz, 1H), 7.49 (t, *J* = 7.7 Hz, 2H), 5.90 (d, *J* = 4.3 Hz, 1H), 2.39–2.29 (m, 1H), 1.07 (d, *J* = 6.8 Hz, 3H), 0.94 (d, *J* = 6.8 Hz, 3H). ^13^C NMR (126 MHz, CDCl_3_) δ 195.7, 160.5, 135.3, 133.6, 128.9, 128.4, 78.9, 30.2, 19.5, 16.7. IR (KBr): 2963, 2360, 2341, 1730, 1696, 1260, 1094, 1019, 799 cm^−1^. HRMS-ESI (*m*/*z*): [M + H]^+^ calcd for C_12_H_14_O_3_, 207.1021; found: 207.1023.

#### 4.6.11. 1-Oxo-1-phenylhexan-2-yl Formate (**2k**)

According to the general procedure, **1k** (1.0 mmol, 176.1 mg), CuBr_2_ (2.0 mmol, 446.7 mg), TMSCF_3_ (2.0 mmol, 284.4 mg), and H_2_O (1.0 mmol, 18.0 mg) were added to DMF (3.0 mL) to produce **2k** (165.8 mg, 75% yield), which was isolated as a colorless oil after purification through silica gel chromatography (petroleum ether:ethyl acetate = 85:1). ^1^H NMR (500 MHz, CDCl_3_) δ 8.16 (s, 1H), 7.95 (d, *J* = 7.8 Hz, 2H), 7.60 (t, *J* = 7.4 Hz, 1H), 7.49 (t, *J* = 7.6 Hz, 2H), 6.01 (dd, *J* = 8.6, 4.1 Hz, 1H), 1.97–1.79 (m, 2H), 1.47–1.42 (m, 2H), 1.40–1.33 (m, 2H), 0.89 (t, *J* = 7.2 Hz, 3H). ^13^C NMR (126 MHz, CDCl_3_) δ 195.6, 160.3, 134.5, 133.7, 128.8, 128.4, 74.8, 31.1, 27.4, 22.2, 13.8. IR (KBr): 3064, 2959, 2932, 2872, 1728, 1697, 1598, 1581, 1449, 1171 cm^−1^. HRMS-ESI (*m*/*z*): [M + H]^+^ calcd for C_13_H_16_O_3_, 221.1178; found: 221.1176.

#### 4.6.12. 2-Methyl-1-oxo-1-phenylpropan-2-yl Formate (**2l**)

According to the general procedure, **1l** (1.0 mmol, 148.1 mg), CuBr_2_ (2.0 mmol, 446.7 mg), TMSCF_3_ (2.0 mmol, 284.4 mg), and H_2_O (1.0 mmol, 18.0 mg) were added to DMF (3.0 mL) to produce **2l** (134.6 mg, 70% yield), which was isolated as a colorless oil after purification through silica gel chromatography (petroleum ether:ethyl acetate = 110:1). ^1^H NMR (500 MHz, CDCl_3_) δ 8.01 (d, *J* = 7.1 Hz, 2H), 7.86 (s, 1H), 7.51 (t, *J* = 7.4 Hz, 1H), 7.41 (t, *J* = 7.8 Hz, 2H), 1.76 (s, 6H). ^13^C NMR (126 MHz, CDCl_3_) δ 198.5, 160.0, 134.1, 132.7, 128.8, 128.4, 84.9, 25.4. IR (KBr): 3063, 2995, 2943, 2353, 1970, 1722, 1685, 1598, 1467, 1385, 1280, 1137, 713 cm^−1^. HRMS-ESI (*m*/*z*): [M + H]^+^ calcd for C_11_H_12_O_3_, 193.0865; found: 193.0864.

#### 4.6.13. 2-Oxo-1,2-diphenylethyl Formate (**2m**)

According to the general procedure, **1m** (1.0 mmol, 196.1 mg), CuBr_2_ (2.0 mmol, 446.7 mg), TMSCF_3_ (2.0 mmol, 284.4 mg), and H_2_O (1.0 mmol, 18.0 mg) were added to DMF (3.0 mL) to produce **2m** (170.9 mg, 71% yield), which was isolated as a colorless oil after purification through silica gel chromatography (petroleum ether:ethyl acetate = 95:1). ^1^H NMR (500 MHz, CDCl_3_) δ 8.21 (s, 1H), 7.93 (d, *J* = 7.3 Hz, 2H), 7.52–7.45 (m, 3H), 7.42–7.33 (m, 5H), 7.00 (s, 1H). ^13^C NMR (126 MHz, CDCl_3_) δ 192.7, 160.0, 134.3, 133.7, 133.1, 129. 6, 129.2, 128.8, 128.7, 77.1. IR (KBr): 2917, 2849, 2360, 1733, 1697, 1472, 1261, 1151, 803 cm^−1^. The NMR data were in agreement with the reported results [73].

#### 4.6.14. 2-Oxo-2-(o-tolyl)ethyl Formate (**2n**)

According to the general procedure, **1n** (1.0 mmol, 134.1 mg), CuBr_2_ (2.0 mmol, 446.7 mg), TMSCF_3_ (2.0 mmol, 284.4 mg), and H_2_O (1.0 mmol, 18.0 mg) were added to DMF (3.0 mL) to produce **2n** (89.7 mg, 50% yield), which was isolated as a colorless oil after purification through silica gel chromatography (petroleum ether:ethyl acetate = 100:1). ^1^H NMR (400 MHz, CDCl_3_) δ 8.16 (s, 1H), 7.57–7.50 (m, 1H), 7.41–7.32 (m, 1H), 7.23 (d, *J* = 7.5 Hz, 2H), 5.21 (s, 2H), 2.45 (s, 3H). ^13^C NMR (101 MHz, CDCl_3_) δ 193.4, 159.0, 138.2, 133.1, 131.3, 127.0, 124.7, 65.5, 28.7, 20.1. IR (KBr): 2934, 2361, 2341, 1733, 1699, 1457, 1161, 934, 823 cm^−1^. HRMS-ESI (*m*/*z*): [M + H]^+^ calcd for C_10_H_10_O_3_, 179.0708; found: 179.0708.

#### 4.6.15. 2-Oxo-2-(m-tolyl)ethyl Formate (**2o**)

According to the general procedure, **1o** (1.0 mmol, 134.1 mg), CuBr_2_ (2.0 mmol, 446.7 mg), TMSCF_3_ (2.0 mmol, 284.4 mg), and H_2_O (1.0 mmol, 18.0 mg) were added to DMF (3.0 mL) to produce **2o** (93.1 mg, 52% yield), which was isolated as a colorless oil after purification through silica gel chromatography (petroleum ether:ethyl acetate = 90:1). ^1^H NMR (400 MHz, CDCl_3_) δ 8.15 (s, 1H), 7.63 (s, 1H), 7.61 (d, *J* = 8.0 Hz, 1H), 7.33 (d, *J* = 8.0 Hz, 1H), 7.28 (d, *J* = 8.0 Hz, 1H), 5.33 (s, 2H), 2.31 (s, 3H). ^13^C NMR (101 MHz, CDCl_3_) δ 190.3, 159.0, 137.8, 133.8, 132.9, 127.7, 127.2, 123.9, 64.4, 20.3. IR (KBr): 3439, 3005, 2363, 1726, 1694, 1607, 1427, 1190, 795 cm^−1^. HRMS-ESI (*m*/*z*): [M + H]^+^ calcd for C_10_H_10_O_3_, 179.0708; found: 179.0708.

#### 4.6.16. 2-Oxo-2-(p-tolyl)ethyl Formate (**2p**)

According to the general procedure, **1p** (1.0 mmol, 134.1 mg), CuBr_2_ (2.0 mmol, 446.7 mg), TMSCF_3_ (2.0 mmol, 284.4 mg), and H_2_O (1.0 mmol, 18.0 mg) were added to DMF (3.0 mL) to produce **2p** (98.1 mg, 55% yield), which was isolated as a colorless oil after purification through silica gel chromatography (petroleum ether:ethyl acetate = 110:1). ^1^H NMR (400 MHz, CDCl_3_) δ 8.18 (s, 1H), 7.75 (d, *J* = 8.3 Hz, 2H), 7.22 (d, *J* = 8.0 Hz, 2H), 5.35 (s, 2H), 2.36 (s, 3H). ^13^C NMR (101 MHz, CDCl_3_) δ 189.6, 159.1, 144.1, 130.4, 128.6, 126.9, 64.2, 20.8. IR (KBr): 3440, 2918, 2360, 1727, 1607, 1422, 1282, 1158, 815 cm^−1^. The NMR data were in agreement with the reported results [74].

#### 4.6.17. 1-(5-Methylfuran-2-yl)-1-oxopropan-2-yl Formate (**2q**)

According to the general procedure, **1q** (1.0 mmol, 138.1 mg), CuBr_2_ (2.0 mmol, 446.7 mg), TMSCF_3_ (2.0 mmol, 284.4 mg), and H_2_O (1.0 mmol, 18.0 mg) were added to DMF (3.0 mL) to produce **2q** (119.4 mg, 66% yield), which was isolated as a colorless oil after purification through silica gel chromatography (petroleum ether:ethyl acetate = 100:1).^1^H NMR (500 MHz, CDCl_3_) δ 8.12 (s, 1H), 7.25 (d, *J* = 3.6 Hz, 1H), 6.22 (d, *J* = 3.5 Hz, 1H), 5.86 (q, *J* = 6.9 Hz, 1H), 2.42 (s, 3H), 1.58 (d, *J* = 7.0 Hz, 3H). ^13^C NMR (126 MHz, CDCl_3_) δ 183.8, 160.0, 158.8, 148.9, 120.9, 109.4, 71.0, 17.4, 14.1. IR (KBr): 3734, 2939, 2360, 2341, 1716, 1669, 1514, 1209, 1168, 1129 cm^−1^. HRMS-ESI (*m*/*z*): [M + H]^+^ calcd for C_9_H_10_O_4_, 183.0657; found: 183.0655.

#### 4.6.18. 1-Oxo-1-(thiophen-2-yl)propan-2-yl Formate (**2r**)

According to the general procedure, **1r** (1.0 mmol, 140.3 mg), CuBr_2_ (2.0 mmol, 446.7 mg), TMSCF_3_ (2.0 mmol, 284.4 mg), and H_2_O (1.0 mmol, 18.0 mg) were added to DMF (3.0 mL) to produce **2r** (121.1 mg, 66% yield), which was isolated as a colorless oil after purification through silica gel chromatography (petroleum ether:ethyl acetate = 110:1). ^1^H NMR (500 MHz, CDCl_3_) δ 8.13 (s, 1H), 7.82 (d, *J* = 3.0 Hz, 1H), 7.72 (d, *J* = 4.9 Hz, 1H), 7.19–7.16 (m, 1H), 5.90 (q, *J* = 6.9 Hz, 1H), 1.63 (d, *J* = 7.1 Hz, 3H). ^13^C NMR (126 MHz, CDCl_3_) δ 188.7, 159.9, 140.3, 134.8, 132.8, 128.3, 71.8, 17.8. IR (KBr): 3101, 2992, 2359, 1726, 1675, 1517, 1414, 1242, 1175 cm^−1^. HRMS-ESI (*m*/*z*): [M + H]^+^ calcd for C_8_H_8_O_3_S, 185.0272; found: 185.0273.

#### 4.6.19. 1-Oxo-2,3-dihydro-1H-inden-2-yl Formate (**2s**)

According to the general procedure, **1s** (1.0 mmol, 132.1 mg), CuBr_2_ (2.0 mmol, 446.7 mg), TMSCF_3_ (2.0 mmol, 284.4 mg), and H_2_O (1.0 mmol, 18.0 mg) were added to DMF (3.0 mL) to produce **2s** (97.2 mg, 55% yield), which was isolated as a colorless oil after purification through silica gel chromatography (petroleum ether:ethyl acetate = 150:1). ^1^H NMR (400 MHz, CDCl_3_) δ 8.15 (s, 1H), 7.72 (d, *J* = 7.7 Hz, 1H), 7.58 (t, *J* = 7.3 Hz, 1H), 7.42–7.32 (m, 2H), 5.51–5.43 (m, 1H), 3.62 (dd, *J* = 17.0, 8.1 Hz, 1H), 3.01 (dd, *J* = 17.0, 4.9 Hz, 1H). ^13^C NMR (101 MHz, CDCl_3_) δ 198.7, 159.1, 149.2, 135.1, 133.2, 127.3, 125.6, 123.5, 72.4, 32.2. IR (KBr): 3431, 2931, 2360, 1715, 1608, 1466, 1155, 997, 885 cm^−1^. The NMR data were in agreement with the reported results [75].

#### 4.6.20. 1-Oxo-1,2,3,4-tetrahydronaphthalen-2-yl Formate (**2t**)

According to the general procedure, **1t** (1.0 mmol, 146.1 mg), CuBr_2_ (2.0 mmol, 446.7 mg), TMSCF_3_ (2.0 mmol, 284.4 mg), and H_2_O (1.0 mmol, 18.0 mg) were added to DMF (3.0 mL) to produce **2t** (95.2 mg, 50% yield), which was isolated as a colorless oil after purification through silica gel chromatography (petroleum ether:ethyl acetate = 110:1). ^1^H NMR (400 MHz, CDCl_3_) δ 8.18 (s, 1H), 7.94 (d, *J* = 7.8 Hz, 1H), 7.43 (t, *J* = 7.5 Hz, 1H), 7.28–7.17 (m, 2H), 5.56 (dd, *J* = 13.4, 5.2 Hz, 1H), 3.20–2.97 (m, 2H), 2.40–2.16 (m, 2H). ^13^C NMR (101 MHz, CDCl_3_) δ 191.1, 158.9, 142.0, 133.1, 130.3, 127.7, 126.8, 126.0, 73.1, 28.0, 26.8. The NMR data were in agreement with the reported results [75].

#### 4.6.21. 1-Oxo-1-(p-tolyl)propan-2-yl Acetate (**3a**)

According to the general procedure, **1a** (1.0 mmol, 148.5 mg), CuBr_2_ (2.0 mmol, 446.7 mg), TMSCF_3_ (2.0 mmol, 284.4 mg), and H_2_O (1.0 mmol, 18.0 mg) were added to DMA (3.0 mL) to produce **3a** (182.0 mg, 88% yield), which was isolated as a colorless oil after purification through silica gel chromatography (petroleum ether:ethyl acetate = 65:1). ^1^H NMR (500 MHz, CDCl_3_) δ 7.84 (d, *J* = 8.2 Hz, 2H), 7.27 (d, *J* = 8.0 Hz, 2H), 5.95 (q, *J* = 7.0 Hz, 1H), 2.41 (s, 3H), 2.14 (s, 3H), 1.52 (d, *J* = 7.1 Hz, 3H).^13^C NMR (126 MHz, CDCl_3_) δ 196.4, 170.4, 144.5, 131.8, 129.5, 128.6, 71.4, 21.7, 20.7, 17.3. The NMR data were in agreement with the reported results [76].

#### 4.6.22. 1-(4-Bromophenyl)-1-oxopropan-2-yl Benzoate (**3c**)

According to the general procedure, **1c** (1.0 mmol, 212.0 mg), CuBr_2_ (2.0 mmol, 446.7 mg), TMSCF_3_ (2.0 mmol, 284.4 mg), and H_2_O (1.0 mmol, 18.0 mg) were added to DMA (3.0 mL) to produce **3c** (215.2 mg, 80% yield), which was isolated as a colorless oil after purification through silica gel chromatography (petroleum ether:ethyl acetate = 160:1). ^1^H NMR (500 MHz, CDCl_3_) δ 7.80 (d, *J* = 8.6 Hz, 2H), 7.62 (d, *J* = 8.6 Hz, 2H), 5.88 (q, *J* = 7.0 Hz, 1H), 2.14 (s, 3H), 1.51 (d, *J* = 7.0 Hz, 3H). ^13^C NMR (126 MHz, CDCl_3_) δ 196.0, 170.4, 133.2, 132.1, 129.9, 128.8, 71.3, 20.7, 17.0. The NMR data were in agreement with the reported results [76].

#### 4.6.23. 1-Oxo-1-phenylpentan-2-yl Acetate (**3i**)

According to the general procedure, **1i** (1.0 mmol, 162.1 mg), CuBr_2_ (2.0 mmol, 446.7 mg), TMSCF_3_ (2.0 mmol, 284.4 mg), and H_2_O (1.0 mmol, 18.0 mg) were added to DMA (3.0 mL) to produce **3i** (164.5 mg, 75% yield), which was isolated as a colorless oil after purification through silica gel chromatography (petroleum ether:ethyl acetate = 35:1). ^1^H NMR (500 MHz, CDCl_3_) δ 7.94 (d, *J* = 7.2 Hz, 2H), 7.59 (t, *J* = 7.4 Hz, 1H), 7.48 (t, *J* = 7.8 Hz, 2H), 5.88 (dd, *J* = 7.7, 5.1 Hz, 1H), 2.16 (s, 3H), 1.89–1.79 (m, 2H), 1.55–1.39 (m, 2H), 0.95 (t, *J* = 7.4 Hz, 3H). ^13^C NMR (126 MHz, CDCl_3_) δ 196.7, 170.7, 134.9, 133.5, 128.8, 128.4, 75.2, 33.4, 20.7, 18.9, 13.7. The NMR data were in agreement with the reported results [76].

#### 4.6.24. 1-Oxo-1-phenylhexan-2-yl Acetate (**3k**)

According to the general procedure, **1k** (1.0 mmol, 176.1 mg), CuBr_2_ (2.0 mmol, 446.7 mg), TMSCF_3_ (2.0 mmol, 284.4 mg), and H_2_O (1.0 mmol, 18.0 mg) were added to DMA (3.0 mL) to produce **3k** (174.1 mg, 74% yield), which was isolated as a colorless oil after purification through silica gel chromatography (petroleum ether:ethyl acetate = 85:1). ^1^H NMR (500 MHz, CDCl_3_) δ 7.94 (d, *J* = 7.0 Hz, 2H), 7.59 (t, *J* = 7.4 Hz, 1H), 7.48 (t, *J* = 7.8 Hz, 2H), 5.86 (dd, *J* = 8.6, 4.2 Hz, 1H), 2.16 (s, 3H), 2.03–1.62 (m, 2H), 1.50–1.20 (m, 4H), 0.89 (t, *J* = 7.2 Hz, 3H). ^13^C NMR (126 MHz, CDCl_3_) δ 196.7, 170.7, 134.9, 133.5, 128.8, 128.4, 75.4, 31.1, 27.7, 22.3, 20.7, 13.8. IR (KBr): 2962, 2933, 2874, 2364, 1740, 1696, 1597, 1448, 1375, 1230, 697 cm^−1^. The NMR data were in agreement with the reported results [77].

#### 4.6.25. 2-Oxo-1,2-diphenylethyl Acetate (**3m**)

According to the general procedure, **1m** (1.0 mmol, 196.1 mg), CuBr_2_ (2.0 mmol, 446.7 mg), TMSCF_3_ (2.0 mmol, 284.4 mg), and H_2_O (1.0 mmol, 18.0 mg) were added to DMA (3.0 mL) to produce **3m** (178.2 mg, 70% yield), which was isolated as a white solid powder (m.p. = 72–73 °C) after purification through silica gel chromatography (petroleum ether:ethyl acetate = 110:1). ^1^H NMR (500 MHz, CDCl_3_) δ 7.95–7.91 (m, 2H), 7.52–7.46 (m, 3H), 7.41–7.33 (m, 5H), 6.86 (s, 1H), 2.20 (s, 3H). ^13^C NMR (126 MHz, CDCl_3_) δ 193.8, 170.5, 134.7, 133.7, 133.5, 129.3, 129.2, 128.8, 128.7, 128.7, 77.7, 20.8. The NMR data were in agreement with the reported results [77].

#### 4.6.26. 1-(5-Methylfuran-2-yl)-1-oxopropan-2-yl Acetate (**3q**)

According to the general procedure, **1q** (1.0 mmol, 138.1 mg), CuBr_2_ (2.0 mmol, 446.7 mg), TMSCF_3_ (2.0 mmol, 284.4 mg), and H_2_O (1.0 mmol, 18.0 mg) were added to DMA (3.0 mL) to produce **3q** (116.9 mg, 60% yield), which was isolated as a colorless oil after purification through silica gel chromatography (petroleum ether:ethyl acetate = 150:1). ^1^H NMR (500 MHz, CDCl_3_) δ 7.22 (d, *J* = 3.5 Hz, 1H), 6.19 (d, *J* = 3.4 Hz, 1H), 5.70 (q, *J* = 7.0 Hz, 1H), 2.41 (s, 3H), 2.14 (s, 3H), 1.53 (d, *J* = 7.0 Hz, 3H). ^13^C NMR (126 MHz, CDCl_3_) δ 184.8, 170.3, 158.5, 149.1, 120.6, 109.3, 71.4, 20.7, 17.3, 14.1. IR (KBr): 3122, 2992, 2360, 2341, 1742, 1682, 1515, 1372, 1238, 1040 cm^−1^. HRMS-ESI (*m*/*z*): [M + H]^+^ calcd for C_10_H_12_O_4_, 197.0814; found: 197.0812.

#### 4.6.27. 1-Oxo-1-(thiophen-2-yl)propan-2-yl Acetate (**3r**)

According to the general procedure, **1r** (1.0 mmol, 140.1 mg), CuBr_2_ (2.0 mmol, 446.7 mg), TMSCF_3_ (2.0 mmol, 284.4 mg), and H_2_O (1.0 mmol, 18.0 mg) were added to DMA (3.0 mL) to produce **3r** (119.6 mg, 60% yield), which was isolated as a colorless oil after purification through silica gel chromatography (petroleum ether:ethyl acetate = 100:1). ^1^H NMR (500 MHz, CDCl_3_) δ 7.81 (d, *J* = 4.6 Hz, 1H), 7.70 (d, *J* = 5.7 Hz, 1H), 7.19–7.13 (m, 1H), 5.74 (q, *J* = 7.0 Hz, 1H), 2.16 (s, 3H), 1.58 (d, *J* = 7.1 Hz, 3H). ^13^C NMR (126 MHz, CDCl_3_) δ 189.7, 170.3, 140.5, 134.4, 132.6, 128.2, 72.4, 20.8, 17.7. IR (KBr): 3091, 2964, 2928, 2328, 1741, 1669, 1517, 1448, 1413, 1235, 1080, 798 cm^−1^. The NMR data were in agreement with the reported results [76].

#### 4.6.28. 2-Oxo-2-phenylethyl Acetate (**3u**)

According to the general procedure, **1u** (1.0 mmol, 120.2 mg), CuBr_2_ (2.0 mmol, 446.7 mg), TMSCF_3_ (2.0 mmol, 284.4 mg), and H_2_O (1.0 mmol, 18.0 mg) were added to DMA (3.0 mL) to produce **3u** (137.6 mg, 77% yield), which was isolated as a colorless oil after purification through silica gel chromatography (petroleum ether:ethyl acetate = 90:1). ^1^H NMR (500 MHz, CDCl_3_) δ 7.91 (d, *J* = 7.3 Hz, 2H), 7.60 (t, *J* = 7.4 Hz, 1H), 7.48 (t, *J* = 7.7 Hz, 2H), 5.34 (s, 2H), 2.22 (s, 3H). ^13^C NMR (126 MHz, CDCl_3_) δ 192.2, 170.4, 134.2, 133.9, 128.9, 127.8, 66.0, 20.5. The NMR data were in agreement with the reported results [78].

#### 4.6.29. 1-Oxo-1-(p-tolyl)propan-2-yl Benzoate (**4a**)

According to the general procedure, the combination of **1a** (1.0 mmol, 148.1 mg), CuBr_2_ (2.0 mmol, 446.7 mg), TMSCF_3_ (2.0 mmol, 284.4 mg), H_2_O (1.0 mmol, 18.0 mg), and N,N-dimethylbenzamide (3 g) produced **4a** (185.5 mg, 70% yield), which was isolated as a white solid powder (m.p. = 67–68 °C) after purification through silica gel chromatography (petroleum ether:ethyl acetate = 190:1). ^1^H NMR (400 MHz, CDCl_3_) δ 7.98 (d, *J* = 7.0 Hz, 2H), 7.79 (d, *J* = 8.3 Hz, 2H), 7.43 (t, *J* = 7.5 Hz, 1H), 7.30 (t, *J* = 7.8 Hz, 2H), 7.14 (d, *J* = 8.0 Hz, 2H), 6.07 (q, *J* = 7.0 Hz, 1H), 2.26 (s, 3H), 1.54 (d, *J* = 7.0 Hz, 3H). ^13^C NMR (101 MHz, CDCl_3_) δ 195.1, 164.9, 143.4, 132.2, 130.8, 128.8, 128.5, 128.4, 127.6, 127.3, 70.8, 20.6, 16.2. The NMR data were in agreement with the reported results [79].

Crystal Data for C_17_H_16_O_3_ (M =268.30 g/mol): monoclinic, space group P2_1_/c (no. 14), *a* = 20.4570(9) Å, *b* = 9.1734(4) Å, *c* = 16.0710(7) Å, *β* = 109.656(5)°, *V* = 2840.1(2) Å^3^, *Z* = 8, *T* = 123.15 K, μ (MoKα) = 0.085 mm^−1^, *Dcalc* = 1.255 g/cm^3^, 29584 reflections measured (4.228° ≤ 2Θ ≤ 52.744°), with 5809 unique (*R*_int_ = 0.0506, R_sigma_ = 0.0337) which were used in all of the calculations. The final *R*_1_ was 0.0443 (I > 2σ(I)) and the *wR*_2_ was 0.1143 (all data).

#### 4.6.30. 2-Oxopentan-3-yl Benzoate (**4v**)

According to the general procedure, the combination of **1v** (1.0 mmol, 86.1 mg), CuBr_2_ (2.0 mmol, 446.7 mg), TMSCF_3_ (2.0 mmol, 284.4 mg), H_2_O (1.0 mmol, 18.0 mg), and N,N-dimethylbenzamide (3 g) produced **4v** (113.0 mg, 55% yield), which was isolated as a colorless oil after purification through silica gel chromatography (petroleum ether:ethyl acetate = 200:1). ^1^H NMR (400 MHz, CDCl_3_) δ 8.02 (d, *J* = 7.0 Hz, 2H), 7.52 (t, *J* = 7.5 Hz, 1H), 7.39 (t, *J* = 7.7 Hz, 2H), 5.11 (dd, *J* = 7.7, 4.8 Hz, 1H), 2.14 (s, 3H), 1.99–1.78 (m, 2H), 0.99 (t, *J* = 7.4 Hz, 3H). ^13^C NMR (101 MHz, CDCl_3_) δ 204.5, 165.1, 132.4, 128.7, 128.4, 127.5, 79.1, 25.3, 22.9, 8.6. IR (KBr): 3065, 2973, 2361, 1717, 1602, 1452, 1271, 1177, 1110, 711 cm^−1^. HRMS-ESI (*m*/*z*): [M + H]^+^ calcd for C_12_H_14_O_3_, 207.1021; found: 207.1022.

#### 4.6.31. 2-Oxohexan-3-yl Benzoate (**4w**)

According to the general procedure, the combination of **1w** (1.0 mmol, 100.1 mg), CuBr_2_ (2.0 mmol, 446.7 mg), TMSCF_3_ (2.0 mmol, 284.4 mg), H_2_O (1.0 mmol, 18.0 mg), and N,N-dimethylbenzamide (3 g) produced **4w** (102.8 mg, 50% yield) which was isolated as a colorless oil after purification through silica gel chromatography (petroleum ether:ethyl acetate = 200:1).^1^H NMR (400 MHz, CDCl_3_) δ 7.99 (d, *J* = 7.1 Hz, 2H), 7.48 (t, *J* = 7.4 Hz, 1H), 7.36 (t, *J* = 7.7 Hz, 2H), 5.14 (t, *J* = 6.4 Hz, 1H), 2.11 (s, 3H), 1.82–1.73 (m, 2H), 1.48–1.35 (m, 2H), 0.88 (t, *J* = 7.4 Hz, 3H). ^13^C NMR (101 MHz, CDCl_3_) δ 204.5, 165.0, 132.4, 128.7, 128.4, 127.5, 78.0, 31.5, 25.1, 17.6, 12.7. IR (KBr): 2962, 2875, 2361, 1719, 1602, 1452, 1277, 1113, 1071, 1027, 713 cm^−1^. HRMS-ESI (*m*/*z*): [M + H]^+^ calcd for C_13_H_16_O_3_, 221.1178; found: 221.1176.

#### 4.6.32. 1-Oxo-1-(p-tolyl)propan-2-yl Propionate (**5a**)

According to the general procedure, the combination of **1a** (1.0 mmol, 148.5 mg), CuBr_2_ (2.0 mmol, 446.7 mg), TMSCF_3_ (2.0 mmol, 284.4 mg), and H_2_O (1.0 mmol, 18.0 mg) N,N-dimethylpropionamide (3.0 mL) produced **5a** (175.7 mg, 80% yield), which was isolated as a colorless oil after purification through silica gel chromatography (petroleum ether:ethyl acetate = 50:1). ^1^H NMR (400 MHz, CDCl_3_) δ 7.75 (d, *J* = 8.2 Hz, 2H), 7.17 (d, *J* = 8.0 Hz, 2H), 5.87 (q, *J* = 7.0 Hz, 1H), 2.38–2.33 (m, 2H), 2.31 (s, 3H), 1.42 (d, *J* = 7.1 Hz, 3H), 1.06 (t, *J* = 7.6 Hz, 3H). ^13^C NMR (101 MHz, CDCl_3_) δ 195.5, 172.8, 143.4, 130.8, 128.4, 127.5, 70.1, 26.3, 20.6, 16.2, 7.9. IR (KBr): 3034, 2985, 2940, 2359, 2330, 1738, 1694, 1606, 1572, 1183 cm^−1^. HRMS-ESI (*m*/*z*): [M + H]^+^ calcd for C_13_H_16_O_3_, 221.1178; found: 221.1176.

## Figures and Tables

**Table 1:** ^1^H NMR experiments with TMSCF_3_, H_2_O, and DMF ^a^.

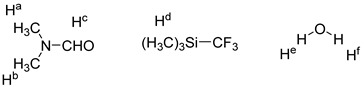						
Chemical Shift	H^a^	H^b^	H^c^	H^d^	H^e^	H^f^
DMF	2.48	2.60	7.62	-	-	-
TMSCF_3_	-	-	-	0.27	-	-
DMF + TMSCF_3_ ^b^	2.76	2.84	7.89	0.15		
H_2_O	-	-	-	-	4.79	4.75
H_2_O + TMSCF_3_ ^c^	-	-	-	0.27	4.81	4.81

^a^ The experiments were carried out using CDCl_3_. ^b^ TMSCF_3_ (0.1 mol), DMF (0.1 mol), ^c^ TMSCF_3_ (0.1 mol), and H_2_O (0.1 mol).

**Table 2:** Optimization of reaction conditions ^a^.

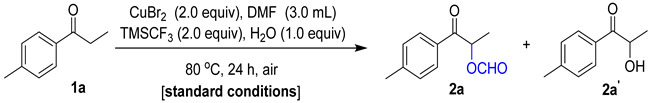			
Entry	Conditions	2a (%) ^b^	2a′ (%) ^b^
1	Standard conditions	95	- ^c^
2	Without TMSCF_3_	65	23
3	CuCl_2_ instead of CuBr_2_	41	- ^c^
4	CuI instead of CuBr_2_	77	16
5	CuBr instead of CuBr_2_	45	- ^c^
6	CuCl instead of CuBr_2_	21	- ^c^
7	Without CuBr_2_	- ^c^	- ^c^
8	1.0 mmol CuBr_2_	44	- ^c^
9	3.0 mmol CuBr_2_	84	- ^c^
10	Without H_2_O	35	- ^c^
11	With Ar protection	93	- ^c^

^a^ Reaction conditions: **1a** (1.0 mmol), metal catalyst (2.0 mmol), TMSCF_3_ (2.0 mmol), H_2_O (1.0 mmol), and DMF (3.0 mL), 80 °C, 24 h. ^b^ Isolated yields. ^c^ Not detected.

## Data Availability

Data supporting the findings of this study are included in the Article and its Supplementary Information. Data are also available upon request.

## Supplementary Materials

The following supporting information can be downloaded at https://www.mdpi.com/article/10.3390/molecules30051114/s1, Screening of reaction conditions, DFT calculation results, characterization data for the products. CCDC 2082077 contains the supplementary crystallographic data for this paper. The data can be obtained free of charge via www.ccdc.cam.ac.uk/data_request/cif (accessed on 20 January 2025), by emailing data_request@ccdc.cam.ac.uk, or by contacting The Cam-bridge Crystallographic Data Centre, 12 Union Road, Cam-bridge CB2 1EZ, UK; Fax: +44-1223-336033. References [80,81] are cited in the Supplementary Materials.

## References

[1] Tietze L.F. (1996). Domino reactions in organic synthesis. Chem. Rev..

[2] Nicolaou K.C., Edmonds D.J., Bulger P.G. (2006). Cascade reactions in total synthesis. Angew. Chem. Int. Ed..

[3] Grondal C., Jeanty M., Enders D. (2010). Organocatalytic cascade reactions as a new tool in total synthesis. Nat. Chem..

[4] Volla C.M.R., Atodiresei I., Rueping M. (2014). Catalytic C–C bond-forming multi-component cascade or domino reactions: Pushing the boundaries of complexity in asymmetric organocatalysis. Chem. Rev..

[5] Ardkhean R., Caputo D.F.J., Morrow S.M., Shi H., Xiong Y., Anderson E.A. (2016). Cascade polycyclizations in natural product synthesis. Chem. Soc. Rev..

[6] Chauhan P., Mahajan S., Enders D. (2017). Achieving Molecular Complexity via Stereoselective Multiple Domino Reactions Promoted by a Secondary Amine Organocatalyst. Acc. Chem. Res..

[7] Zhang M., Gong Y., Zhou W., Zhou Y., Liu X.-L. (2021). Recent advances of chromone-based reactants in the catalytic asymmetric domino annulation reaction. Org. Chem. Front..

[8] Okiko Miyata T.M. (2013). Masafumi Ueda, Umpolung reactions at the α-carbon position of carbonyl compounds. ARKIVOC.

[9] Zhu L., Wang D., Jia Z., Lin Q., Huang M., Luo S. (2018). Catalytic asymmetric oxidative enamine transformations. ACS Catal..

[10] Paul A., Seidel D. (2019). α-Functionalization of cyclic secondary amines: Lewis acid promoted addition of organometallics to transient imines. J. Am. Chem. Soc..

[11] Shen J., Yue X., Xu J., Li W. (2023). α-Amino radical-mediated difunctionalization of alkenes with polyhaloalkanes and N-heteroarenes. Org. Lett..

[12] Jia H., Tan Z., Zhang M. (2024). Reductive functionalization of pyridine-fused N-heteroarenes. Acc. Chem. Res..

[13] Wang F., Frankowski K.J. (2024). Electrochemical α-functionalization of N-aryl-activated tertiary amines. Adv. Synth. Catal..

[14] Erian A.W., Sherif S.M., Gaber H.M. (2003). The chemistry of α-haloketones and their utility in heterocyclic synthesis. Molecules.

[15] Evans R.W., Zbieg J.R., Zhu S., Li W., MacMillan D.W.C. (2013). Simple catalytic mechanism for the direct coupling of α-carbonyls with functionalized amines: A one-step synthesis of plavix. J. Am. Chem. Soc..

[16] Du J., Zhang X., Sun X., Wang L. (2015). Copper-catalyzed direct α-ketoesterification of propiophenones with acetophenones via C(sp3)–H oxidative cross-coupling. Chem. Commun..

[17] Guha S., Rajeshkumar V., Kotha S.S., Sekar G. (2015). A versatile and one-pot strategy to synthesize α-amino ketones from benzylic secondary alcohols using N-bromosuccinimide. Org. Lett..

[18] Liang S., Zeng C.-C., Tian H.-Y., Sun B.-G., Luo X.-G., Ren F.-z. (2016). Electrochemically oxidative α-C–H functionalization of ketones: A cascade synthesis of α-amino ketones mediated by NH_4_I. J. Org. Chem..

[19] Liu L., Feng S., Li C. (2016). Practical approach for quantitative green esterifications. ACS Sust. Chem. Eng..

[20] Chen C., Liu W., Zhou P., Liu H. (2017). I_2_/TBHP-mediated oxidative coupling of ketones and toluene derivatives: A facile method for the preparation of α-benzoyloxy ketones. RSC Adv..

[21] Tan L., Chen C., Liu W. (2017). α-Acetoxyarone synthesis via iodine-catalyzed and tert-butyl hydroperoxide-mediateded self-intermolecular oxidative coupling of aryl ketones. Beilstein J. Org. Chem..

[22] Rossi L.I., Krapacher C.R., Granados A.M. (2020). α-Amination reaction of different ketones mediated by carbohydrate Cu^2+^ complexes. Mol. Catal..

[23] Pogaku N., Krishna P.R., Prapurna Y.L. (2019). Iodine-mediated nucleophilic direct oxidative α-acetoxylation and α-alkoxylation of ketones. ChemistrySelect.

[24] Shibatomi K. (2023). Organocatalytic Synthesis of chiral halogenated compounds. Chem. Rec..

[25] Merritt E.A., Olofsson B. (2011). α-Functionalization of carbonyl compounds using hypervalent iodine reagents. Synthesis.

[26] Dong D.-Q., Hao S.-H., Wang Z.-L., Chen C. (2014). Hypervalent iodine: A powerful electrophile for asymmetric α-functionalization of carbonyl compounds. Org. Biomol. Chem..

[27] Yoshimura A., Zhdankin V.V. (2016). Advances in synthetic applications of hypervalent iodine compounds. Chem. Rev..

[28] Arava S., Kumar J.N., Maksymenko S., Iron M.A., Parida K.N., Fristrup P., Szpilman A.M. (2017). Enolonium species—Umpoled enolates. Angew. Chem. Int. Ed..

[29] Hari D.P., Caramenti P., Waser J. (2018). Cyclic hypervalent iodine reagents: Enabling tools for bond disconnection via reactivity umpolung. Acc. Chem. Res..

[30] Blom J., Reyes-Rodríguez G.J., Tobiesen H.N., Lamhauge J.N., Iversen M.V., Barløse C.L., Hammer N., Rusbjerg M., Jørgensen K.A. (2019). Umpolung strategy for α-functionalization of aldehydes for the addition of thiols and other nucleophiles. Angew. Chem. Int. Ed..

[31] Wang Z., Bi X., Liang Y., Liao P., Dong D. (2014). A copper-catalyzed formal O–H insertion reaction of α-diazo-1,3-dicarbonyl compounds to carboxylic acids with the assistance of isocyanide. Chem. Commun..

[32] Tan F., Liu X., Hao X., Tang Y., Lin L., Feng X. (2016). Asymmetric catalytic insertion of α-diazo carbonyl compounds into O–H bonds of carboxylic acids. ACS Catal..

[33] Yuan W., Eriksson L., Szabó K.J. (2016). Rhodium-catalyzed geminal oxyfluorination and oxytrifluoro-methylation of diazocarbonyl compounds. Angew. Chem. Int. Ed..

[34] Zhang T.-S., Hao W.-J., Wang N.-N., Li G., Jiang D.-F., Tu S.-J., Jiang B. (2016). Catalytic oxidative carbene coupling of α-diazo carbonyls for the synthesis of β-amino ketones via C(sp3)–H functionalization. Org. Lett..

[35] Miyoshi T., Miyakawa T., Ueda M., Miyata O. (2011). Nucleophilic α-arylation and α-alkylation of ketones by polarity inversion of N-alkoxyenamines: Entry to the umpolung reaction at the α-carbon position of carbonyl compounds. Angew. Chem. Int. Ed..

[36] Ren S., Song S., Ye L., Feng C., Loh T.-P. (2016). Copper-catalyzed oxyamination of electron-deficient alkenes with N-acyloxyamines. Chem. Commun..

[37] Xu Z., Chen H., Wang Z., Ying A., Zhang L. (2016). One-pot synthesis of benzene-fused medium-ring ketones: Gold catalysis-enabled enolate umpolung reactivity. J. Am. Chem. Soc..

[38] Carroll F.I., Blough B.E., Abraham P., Mills A.C., Holleman J.A., Wolckenhauer S.A., Decker A.M., Landavazo A., McElroy K.T., Navarro H.A. (2009). Synthesis and biological evaluation of bupropion analogues as potential pharmacotherapies for cocaine addiction. J. Med. Chem..

[39] Lukas R.J., Muresan A.Z., Damaj M.I., Blough B.E., Huang X., Navarro H.A., Mascarella S.W., Eaton J.B., Marxer-Miller S.K., Carroll F.I. (2010). Synthesis and characterization of in vitro and in vivo profiles of hydroxybupropion analogues: Aids to smoking cessation. J. Med. Chem..

[40] Chagnon F., Guay I., Bonin M.-A., Mitchell G., Bouarab K., Malouin F., Marsault É. (2014). Unraveling the structure–activity relationship of tomatidine, a steroid alkaloid with unique antibiotic properties against persistent forms of Staphylococcus aureus. Eur. J. Med. Chem..

[41] Talamas F.X., Abbot S.C., Anand S., Brameld K.A., Carter D.S., Chen J., Davis D., de Vicente J., Fung A.D., Gong L. (2014). Discovery of N-[4-[6-tert-butyl-5-methoxy-8-(6-methoxy-2-oxo-1H-pyridin-3-yl)-3-quinolyl]phenyl]methanesulfonamide (RG7109), a potent inhibitor of the hepatitis C virus NS5B polymerase. J. Med. Chem..

[42] Venkatesh Y., Rajesh Y., Karthik S., Chetan A.C., Mandal M., Jana A., Singh N.D.P. (2016). Photocaging of single and dual (similar or different) carboxylic and amino acids by acetyl carbazole and its application as dual drug delivery in cancer therapy. J. Org. Chem..

[43] Kanda Y., Nakamura H., Umemiya S., Puthukanoori R.K., Murthy Appala V.R., Gaddamanugu G.K., Paraselli B.R., Baran P.S. (2020). Two-phase synthesis of taxol. J. Am. Chem. Soc..

[44] Moriarty R.M., Prakash O., Duncan M.P., Vaid R.K., Musallam H.A. (1987). Hypervalent iodine oxidation of silyl enol ethers under Lewis-acid conditions in methanol. A general route to alpha.-methoxy ketones. J. Org. Chem..

[45] Valgimigli L., Brigati G., Pedulli G.F., DiLabio G.A., Mastragostino M., Arbizzani C., Pratt D.A. (2003). The effect of ring nitrogen atoms on the homolytic reactivity of phenolic compounds: Understanding the radical-scavenging ability of 5-pyrimidinols. Chem.—Eur. J..

[46] Shindo M., Yoshimura Y., Hayashi M., Soejima H., Yoshikawa T., Matsumoto K., Shishido K. (2007). Synthesis of multisubstituted furans, pyrroles, and thiophenes via ynolates. Org. Lett..

[47] Khade V.V., Bhowmick A., Thube A.S., Bhat R.G. (2023). Direct access to strained fused dihalo-aziridino quinoxalinones via C3-alkylation followed by tandem cyclization. J. Org. Chem..

[48] Zhao H., To K.K.W., Lam H., Zhou X., Chan J.F.-W., Peng Z., Lee A.C.Y., Cai J., Chan W.-M., Ip J.D. (2021). Cross-linking peptide and repurposed drugs inhibit both entry pathways of SARS-CoV-2. Nat. Commun..

[49] Zeng Z., Liao C., Yu L. (2024). Molecules for COVID-19 treatment. Chin. Chem. Lett..

[50] Tripathi N., Tripathi N., Goshisht M.K. (2022). COVID-19: Inflammatory responses, structure-based drug design and potential therapeutics. Mol. Divers..

[51] Prakash G.K.S., Panja C., Vaghoo H., Surampudi V., Kultyshev R., Mandal M., Rasul G., Mathew T., Olah G.A. (2006). Facile synthesis of TMS-protected trifluoromethylated alcohols using trifluoromethyltrimethylsilane (TMSCF_3_) and various nucleophilic catalysts in DMF. J. Org. Chem..

[52] Cui B., Sun H., Xu Y., Duan L., Li Y.-M. (2017). MgCl_2_-catalyzed trifluoromethylation of carbonyl compounds using (trifluoromethyl)trimethylsilane as the trifluoromethylating agent. Tetrahedron.

[53] Becke A.D. (1988). A multicenter numerical integration scheme for polyatomic molecules. J. Chem. Phys..

[54] Lu T., Chen F. (2012). Multiwfn: A multifunctional wavefunction analyzer. J. Comput. Chem..

[55] Lu T. (2024). A comprehensive electron wavefunction analysis toolbox for chemists, Multiwfn. J. Chem. Phys..

[56] Parr R.G. (1980). Density Functional Theory of Atoms and Molecules.

[57] Cui B., Zheng Y., Sun H., Shang H., Du M., Shang Y., Yavuz C.T. (2024). Catalytic enantioselective intramolecular hydroamination of alkenes using chiral aprotic cyclic urea ligand on manganese (II). Nat. Commun..

[58] Lee C., Yang W., Parr R.G. (1988). Development of the colle-salvetti correlation-energy formula into a functional of the electron density. Phys. Rev. B.

[59] Becke A.D. (1993). Density-functional thermochemistry. III. The role of exact exchange. J. Chem. Phys..

[60] Becke A.D. (1988). Density-functional exchange-energy approximation with correct asymptotic behavior. Phys. Rev. A.

[61] Grimme S., Antony J., Ehrlich S., Krieg H. (2010). A consistent and accurate ab initio parametrization of density functional dispersion correction (DFT-D) for the 94 elements H-Pu. J. Chem. Phys..

[62] Grimme S., Ehrlich S., Goerigk L. (2011). Effect of the damping function in dispersion corrected density functional theory. J. Comput. Chem..

[63] Hariharan P.C., Pople J.A. (1973). The influence of polarization functions on molecular orbital hydrogenation energies. Theoret. Chim. Acta..

[64] Ditchfield R., Hehre W.J., Pople J.A. (1971). Self-consistent molecular-orbital methods. IX. An extended gaussian-type basis for molecular-orbital studies of organic molecules. J. Chem. Phys..

[65] Marenich A.V., Cramer C.J., Truhlar D.G. (2009). Universal solvation model based on solute electron density and on a continuum model of the solvent defined by the bulk dielectric constant and atomic surface tensions. J. Phys. Chem. B.

[66] Chai J.-D., Head-Gordon M. (2008). Long-range corrected hybrid density functionals with damped atom–atom dispersion corrections. Phys. Chem. Chem. Phys..

[67] Weigend F., Ahlrichs R. (2005). Balanced basis sets of split valence, triple zeta valence and quadruple zeta valence quality for H to Rn: Design and assessment of accuracy. Phys. Chem. Chem. Phys..

[68] Weigend F. (2006). Accurate coulomb-fitting basis sets for H to Rn. Phys. Chem. Chem. Phys..

[69] Merrick J.P., Moran D., Radom L. (2007). An evaluation of harmonic vibrational frequency scale factors. J. Phys. Chem. A.

[70] Lu T., Chen Q. (2021). Shermo: A general code for calculating molecular thermochemistry properties. Comput. Theor. Chem..

[71] Humphrey W., Dalke A., Schulten K. (1996). VMD: Visual molecular dynamics. J. Mol. Graph..

[72] Houghton T.J., Tanaka K.S.E., Kang T., Dietrich E., Lafontaine Y., Delorme D., Ferreira S.S., Viens F., Arhin F.F., Sarmiento I. (2008). Linking bisphosphonates to the free amino groups in fluoroquinolones: Preparation of osteotropic prodrugs for the prevention of osteomyelitis. J. Med. Chem..

[73] Ramanjaneyulu B.T., Vidyacharan S., Yim S.J., Kim D.P. (2019). Fast-synthesis of α-phosphonyloxy ketones as drug scaffolds in a capillary microreactor. Eur. J. Org. Chem..

[74] Zhu Y.-P., Gao Q.-H., Lian M., Yuan J.-J., Liu M.-C., Zhao Q., Yang Y., Wu A.-X. (2011). A sustainable byproduct catalyzed domino strategy: Facile synthesis of α-formyloxy and acetoxy ketones via iodination/nucleophilic substitution/hydrolyzation/oxidation sequences. Chem. Commun..

[75] Lee J.C., Jin Y.S., Choi J.-H. (2001). Synthesis of α-acetoxy and formyloxy ketones by thallium (III) promoted α-oxidation. Chem. Commun..

[76] Hokamp T., Wirth T. (2020). Hypervalent iodine(III)-catalysed enantioselective α-acetoxylation of ketones. Chem.—Eur. J..

[77] Zhang Z., Li P. (2020). Sulfur-mediated difunctionalization of internal and terminal alkynes for the synthesis of α-acetoxy ketones. Tetrahedron Lett..

[78] Chen M., Zhang W., Ren Z.-H., Gao W.-Y., Wang Y.-Y., Guan Z.-H. (2017). PhI(OAc)_2_-promoted umpolung acetoxylation of enamides for the synthesis of α-acetoxy ketones. Sci. China. Chem..

[79] Zhu M., Wei W., Yang D., Cui H., Cui H., Sun X., Wang H. (2016). NBS/DBU mediated one-pot synthesis of α-acyloxyketones from benzylic secondary alcohols and carboxylic acids. Org. Biomol. Chem..

[80] Dolomanov O.V., Bourhis L.J., Gildea R.J., Howard J.A.K., Puschmann H. (2009). OLEX2: A complete structure solution, refinement and analysis program. J. Appl. Crystallogr..

[81] Sheldrick G. (2015). Crystal structure refinement with SHELXL. Acta Crystallogr. Sect. C.

