# Supplementary material for: Computational and Experimental Studies on the α-Functionalization of Ketones Using Domino Reactions: A Strategy to Increase Chemoselectivity at the α-Carbon of Ketones
Source: Molecules. 2025 Feb 28;30(5):1114. doi: 10.3390/molecules30051114 (PMC11901711; doi:10.3390/molecules30051114)
Supplement: Supplementary file 1 [file molecules-30-01114-s001.zip › molecules-3463707-supplementary.pdf]

Supporting Information

Computational and Experimental Studies on α-Functionalization of Ketones by Domino Reactions: Exploration of a Strategy to Increase Chemoselectivity at the α-Carbon of Ketones

Hui Sun [1,*], Li-Heng Yang [1], Meng-Yun Fu [1] and Bin Cui [1,*]

[1]Manganese Catalysis and Asymmetric Synthesis Laboratory, Hebei University of Science and Technology, Shijiazhuang 050018, sunhui@hebust.edu.cn(H,S)

*Correspondence: cuibin1989@hebust.edu.cn(B.C.)

Contents

S1. Computational Details S4

S2. The condensed fukui functions and RDG of DMF, H_2O, and their complexes with $TMSCF_3$ S12

S3. Comparison Experiments of Trimethylsilyl Derivatives S14

S4. General Procedure for Control Experiments Processes S15

S5. ORTEP Drawing and Crystal Data for Compound **4a** S15

S6. Rectification of $TMSCF_3$ S16

S7. Reference S17

S8. NMR and IR Spectra S17

S1. Computational Details

Optimized Cartesian coordinates calculated

1a

C	1.697379	0.367023	0.000001
O	2.155622	1.494245	0.000004
C	0.217586	0.153289	0.000001
C	-0.617781	1.280773	-0.000001
C	-0.373197	-1.116553	0.000002
C	-1.998140	1.143031	-0.000002
H	-0.153393	2.269505	-0.000002
C	-1.760032	-1.250226	0.000001
H	0.243779	-2.017424	0.000003
C	-2.596930	-0.127836	-0.000001
H	-2.632587	2.034277	-0.000004
H	-2.204221	-2.249457	0.000002
C	2.598704	-0.843650	-0.000001
H	2.333521	-1.461978	-0.875322
H	2.333524	-1.461980	0.875319
C	4.073116	-0.500101	-0.000003
H	4.345333	0.092134	0.886490
H	4.684246	-1.414923	-0.000005
H	4.345331	0.092136	-0.886495
C	-4.088187	-0.265903	0.000000
H	-4.397857	-1.320271	-0.000006
H	-4.528880	0.222467	0.884286
H	-4.528883	0.222478	-0.884279

$CuBr_2$

Br	0.000000	-1.802303	-0.421976
Cu	0.000000	0.000000	1.018563
Br	0.000000	1.802303	-0.421976

TS1

C	4.703806	0.268329	0.227930
C	4.706032	-1.073183	-0.176445
C	3.483284	-1.762569	-0.174628
C	2.306836	-1.134778	0.211267
C	2.307925	0.213746	0.601750
C	3.527458	0.905996	0.608627
C	1.025112	0.843463	0.991788
C	0.851986	2.238241	0.946536
O	0.049086	0.074514	1.304952
C	5.966801	-1.753766	-0.611674
C	-0.364667	2.928779	1.428873
Cu	-1.558503	-0.326752	0.418342
Br	-3.620252	-1.282055	0.252124
H	5.645773	0.823154	0.249997
H	3.457878	-2.811301	-0.484134
H	1.363897	-1.685475	0.206830
H	3.570262	1.947438	0.934897
H	1.700862	2.856810	0.642391
H	5.956269	-1.934281	-1.699808
H	6.854249	-1.148154	-0.380305
H	6.077825	-2.736386	-0.127325
H	-1.183618	2.232840	1.656785
H	-0.123327	3.491302	2.350290
H	-0.717839	3.683337	0.706327
H	0.106265	1.670981	-0.811554
Br	-0.922378	1.066166	-1.684697

A

C	4.629292	0.785543	-0.124696
C	4.942357	-0.575451	-0.019614
C	3.881154	-1.488972	0.098345
C	2.564210	-1.057276	0.110390
C	2.253918	0.310462	0.010423
C	3.310324	1.226940	-0.108267
C	0.831228	0.713112	0.023221
C	0.459812	2.091382	0.076768
O	-0.039053	-0.197869	-0.001750
C	6.360163	-1.053424	-0.029467
C	-0.942564	2.540365	0.085413
Cu	-1.924145	-0.267006	0.007766
Br	-4.156805	-0.523813	-0.036406
H	5.437076	1.515510	-0.223356
H	4.100145	-2.557260	0.181735
H	1.747778	-1.776045	0.200867
H	3.117620	2.296988	-0.201041
H	1.249364	2.840197	0.152485
H	7.067498	-0.223484	-0.164155
H	6.606536	-1.566652	0.914504
H	6.523179	-1.783698	-0.838441
H	-1.554071	2.005472	-0.664803
H	-1.413086	2.332136	1.066657
H	-1.020592	3.620317	-0.096551

HBr

Br	0.000000	0.000000	0.039555
H	0.000000	0.000000	-1.384421

B

C	1.490356	-1.123835	-0.472722
C	2.370734	-0.147529	0.007835
C	1.829911	1.081295	0.418785
C	0.463215	1.319049	0.354728
C	-0.417544	0.326845	-0.099595
C	0.115733	-0.896735	-0.521201
C	-1.874012	0.656845	-0.189453
C	-2.887972	-0.341596	0.036618
O	-2.218310	1.822249	-0.428482
C	3.845289	-0.400096	0.092945
C	-2.738174	-1.701165	0.594275
H	1.889215	-2.080952	-0.820509
H	2.498240	1.864200	0.789397
H	0.054111	2.285424	0.658870
H	-0.537100	-1.675045	-0.921979
H	-3.899818	0.014820	-0.186912
H	4.130581	-1.316147	-0.443460
H	4.419827	0.441440	-0.324508
H	4.162720	-0.513058	1.143259

H	-1.784768	-1.851683	1.119161
H	-3.567594	-1.920549	1.286293
H	-2.804147	-2.464909	-0.205054

	**C**		
C	4.474723	0.943697	-0.022369
C	4.891576	-0.324303	-0.450080
C	3.967164	-1.382763	-0.403631
C	2.675901	-1.180859	0.052566
C	2.260755	0.099381	0.463517
C	3.176649	1.162171	0.422723
C	0.095691	1.463260	0.986101
C	6.279695	-0.555047	-0.954878
C	0.373308	2.706131	0.238991
H	5.184840	1.774396	-0.035683
H	4.275399	-2.379039	-0.731890
H	1.962826	-2.007077	0.087881
H	2.892499	2.155783	0.770341
H	-0.528747	1.548186	1.887782
H	6.266109	-0.748799	-2.040592
H	6.928229	0.312678	-0.772532
H	6.730680	-1.440409	-0.480407
H	1.021931	3.349461	0.863583
H	-0.555921	3.265759	0.066631
H	0.880074	2.526235	-0.718479
C	0.902880	0.253123	0.974356
O	0.233137	-0.765261	1.340177
Cu	-1.262113	-0.044490	0.339605
Br	-2.359157	-2.049896	-0.125348
Br	-2.638322	1.476936	-0.654948

	**TS2**		
C	4.086856	0.568670	-0.107607
C	4.238798	-0.772840	-0.480271
C	3.159986	-1.646106	-0.263696
C	1.979449	-1.194161	0.302169
C	1.822974	0.162139	0.646217
C	2.897487	1.039146	0.439133
C	-0.144018	1.843248	1.075110
C	5.506081	-1.269750	-1.099875
C	0.501092	3.094606	0.643645
H	4.921799	1.260930	-0.242978
H	3.255572	-2.699112	-0.542075
H	1.151055	-1.886669	0.469341
H	2.837339	2.083343	0.742080
H	-0.988856	1.951670	1.767642
H	5.349965	-1.493476	-2.168612
H	6.313144	-0.527973	-1.024190
H	5.839713	-2.205151	-0.624630
H	1.165642	3.389892	1.481284
H	-0.237300	3.894148	0.511330
H	1.116682	2.991237	-0.257902
C	0.539849	0.542743	1.266237
O	-0.097078	-0.313171	1.942230
Cu	-1.254311	-0.152811	0.322451
Br	-2.373462	-2.128233	-0.117817
Br	-1.658093	1.727429	-1.011513

	**D**		
C	-0.640158	1.175900	0.069424
O	-0.891234	2.230620	-0.476160
C	0.741285	0.623447	0.090828
C	1.099056	-0.546074	0.774164
C	1.738905	1.327954	-0.601747
C	2.416950	-0.994072	0.762470
H	0.354527	-1.126961	1.321357
C	3.049806	0.876599	-0.611391
H	1.458723	2.240103	-1.132977
C	3.414959	-0.295096	0.072303
H	2.677938	-1.908595	1.301688
H	3.812639	1.438579	-1.157876
C	-1.760803	0.401046	0.757235
H	-1.398654	-0.088569	1.669719
C	-2.982875	1.235836	1.026594
H	-3.354060	1.706955	0.106724
H	-3.781130	0.628452	1.474501
H	-2.718175	2.035227	1.737509
C	4.833597	-0.772521	0.058105
H	5.181466	-0.940432	-0.973962
H	5.505409	-0.018802	0.500189
H	4.951873	-1.709853	0.619121
Br	-2.200715	-1.094262	-0.458418

	**CuBr**		
Br	0.000000	0.000000	1.029727
Cu	0.000000	0.000000	-1.242774

	**TS3**		
C	1.480642	-1.964208	-0.900738
C	2.458865	-2.161357	0.086596
C	2.233964	-1.608208	1.353278
C	1.082462	-0.872431	1.619476
C	0.121864	-0.661728	0.624768
C	0.332284	-1.225645	-0.641244
C	3.711220	-2.923829	-0.220583
C	-1.009554	0.259318	0.955514
C	-1.556145	1.116122	-0.161841
O	-1.325826	0.488994	2.105662
C	-2.546466	2.181494	0.145671
O	0.007796	2.230462	-0.053836
C	0.936577	1.879933	-0.820276
N	2.213655	2.051186	-0.547433
C	3.236787	1.645787	-1.485742
C	2.649812	2.632345	0.703349
Br	-3.164294	-0.953418	-0.462857
H	1.624119	-2.403407	-1.892217
H	2.978370	-1.751391	2.141679
H	0.920062	-0.431263	2.605502
H	-0.426146	-1.112342	-1.418760
H	3.489137	-3.859344	-0.756934
H	4.376890	-2.332473	-0.871960
H	4.269751	-3.169865	0.693528
H	-1.363768	0.847478	-1.198077
H	-3.563498	1.788913	0.026676
H	-2.421189	2.516159	1.183725
H	-2.414812	3.026179	-0.543316
H	0.727184	1.395532	-1.789323
H	2.773388	1.188914	-2.369228
H	3.906663	0.913243	-1.009986
H	3.832706	2.516691	-1.799153
H	1.780644	2.809531	1.346239
H	3.346323	1.942504	1.203102

H	3.169263	3.585072	0.516571

	**TS3s**		
C	2.487699	-0.128269	-1.225653
C	3.385665	-0.015662	-0.151070
C	2.863889	0.313576	1.105125
C	1.499132	0.526975	1.282348
C	0.609716	0.400268	0.211241
C	1.124556	0.068877	-1.051079
C	4.852433	-0.243484	-0.355924
C	-0.830903	0.703078	0.471192
C	-1.712338	1.177846	-0.696256
O	-1.193053	1.146596	1.566274
C	-3.191709	0.981910	-0.582349
Br	-1.545386	-1.801246	0.067241
H	2.869486	-0.387372	-2.217585
H	3.541142	0.410328	1.958657
H	1.098199	0.797340	2.261402
H	0.455663	-0.067293	-1.903024
H	5.263651	0.460148	-1.098088
H	5.415083	-0.120348	0.580241
H	5.045415	-1.258343	-0.740378
H	-1.316825	0.998118	-1.700926
H	-3.414313	-0.065667	-0.815540
H	-3.525295	1.185319	0.445653
H	-3.717700	1.640474	-1.287528
O	-1.54541	2.717329	-0.514313
H	-0.64362	3.013524	-0.771351
H	-1.603514	2.815299	0.473889

	**E**		
C	-4.485741	-0.943930	-0.689857
C	-4.833882	0.369936	-0.333209
C	-3.834624	1.200136	0.189414
C	-2.532571	0.739403	0.363244
C	-2.194328	-0.573507	0.008198
C	-3.188827	-1.406460	-0.528564
C	-0.820289	-1.118872	0.136883
C	0.178632	-0.350116	0.997663
O	-0.483918	-2.149140	-0.411503
C	-6.237801	0.857814	-0.510329
C	-0.151053	-0.412217	2.473481
O	1.478508	-0.926805	0.845114
C	2.059790	-0.767926	-0.304977
N	3.261036	-1.247011	-0.484598
C	4.075333	-1.671422	0.634918
C	3.930003	-1.020379	-1.747291
H	-5.250760	-1.608082	-1.102073
H	-4.081520	2.229250	0.463429
H	-1.780975	1.422122	0.764916
H	-2.920229	-2.426146	-0.813527
H	0.263927	0.699507	0.656815
H	-6.565525	0.743445	-1.556052
H	-6.936609	0.270005	0.107111
H	-6.338083	1.915318	-0.229482
H	0.616242	0.134841	3.039485
H	-1.126348	0.049179	2.674366
H	-0.178895	-1.455857	2.820945
H	1.499287	-0.414874	-1.171276
H	3.445536	-2.09888	1.423013
H	4.789586	-2.42886	0.287602
H	4.628496	-0.807581	1.040316
H	4.491797	-1.921344	-2.029901
H	3.192898	-0.7942	-2.527069
H	4.62115	-0.167799	-1.651737
Br	2.430235	2.016624	-0.183172

	**2a′**		
C	1.438820	0.629182	-0.096672
O	1.827968	1.778933	-0.048621
C	-0.016325	0.311334	-0.054926
C	-0.531286	-0.971352	-0.285830
C	-0.914786	1.361774	0.188104
C	-1.906136	-1.190222	-0.274791
H	0.143865	-1.809069	-0.463189
C	-2.283446	1.136728	0.205653
H	-0.511188	2.362133	0.360192
C	-2.806266	-0.146167	-0.026605
H	-2.291980	-2.196274	-0.461241
H	-2.967683	1.967750	0.400017
C	2.435265	-0.522615	-0.233926
H	2.212922	-0.994511	-1.214392
C	3.868018	-0.039605	-0.232711
H	4.102305	0.471044	0.712947
H	4.550150	-0.896103	-0.350125
H	4.052255	0.660022	-1.060287
C	-4.284468	-0.383148	0.005010
H	-4.679215	-0.248187	1.025860
H	-4.815817	0.335524	-0.638566
H	-4.539495	-1.400127	-0.324350
O	2.175092	-1.424170	0.821098
H	2.723061	-2.205749	0.673479

	**TS4**		
C	-4.099872	-0.649717	-1.192735
C	-4.642360	-0.188761	0.014866
C	-3.770490	0.030640	1.093331
C	-2.409096	-0.213807	0.978824
C	-1.874668	-0.679005	-0.233790
C	-2.736136	-0.881319	-1.320527
C	-0.432022	-0.919015	-0.431462
C	0.453417	-1.172309	0.789401
O	0.046994	-0.968120	-1.557433
C	-6.108522	0.074024	0.159722
C	0.015899	-2.409636	1.552638
O	1.794176	-1.408582	0.375971
C	2.637268	-0.368836	0.291906
N	3.947383	-0.721677	0.261010
C	4.346993	-1.858526	-0.539491
C	4.905735	0.356846	0.340565
H	-4.761258	-0.826193	-2.045194
H	-4.171479	0.404186	2.039514
H	-1.762788	-0.013684	1.835172
H	-2.318866	-1.231613	-2.266980
H	0.417503	-0.276831	1.433347
H	-6.659448	-0.184889	-0.755015
H	-6.531500	-0.505309	0.996062
H	-6.29411	1.137098	0.384336
H	0.700602	-2.570501	2.397038
H	-1.002853	-2.299698	1.943947
H	0.046883	-3.292554	0.896885
H	2.415781	0.483601	0.951475

H	3.605844	-2.661918	-0.450451
H	4.459774	-1.592662	-1.607247
H	5.313256	-2.234399	-0.173639
H	5.084527	0.830141	-0.642903
H	4.547833	1.127877	1.037717
H	5.863656	-0.031994	0.716045
Br	0.568716	2.48996	0.102656
O	2.231211	0.369506	-1.160641
H	1.528623	-0.233241	-1.536248
H	1.685366	1.209805	-0.856552

	**F**		
O	2.749517	0.826180	-1.034615
Br	1.844382	-2.174299	-0.631355
N	2.490569	0.302274	1.236064
C	2.127101	1.236041	0.097229
C	1.756619	0.585069	2.482425
C	3.949120	0.256902	1.446568
O	0.753753	1.171194	0.056279
C	0.068109	2.202656	-0.632219
C	0.147177	2.081269	-2.147094
C	-1.391458	2.204893	-0.179467
C	-2.147737	0.931250	-0.014748
O	-1.931553	3.281846	-0.017127
C	-3.485831	1.033227	0.404218
C	-4.272955	-0.097099	0.558844
C	-3.755490	-1.377646	0.298049
C	-2.423173	-1.474751	-0.118838
C	-1.623765	-0.344826	-0.274325
C	-4.613265	-2.593192	0.464914
H	2.456121	-0.110722	-1.203455
H	2.196342	-0.650533	0.856234
H	2.488258	2.246871	0.363345
H	0.683525	0.468797	2.305152
H	1.978328	1.611257	2.805690
H	2.091732	-0.12522	3.247893
H	4.292005	1.234205	1.81132
H	4.438601	0.014412	0.498115
H	4.167713	-0.518322	2.190293
H	0.475401	3.179874	-0.318524
H	-0.461492	2.872105	-2.610088
H	1.184163	2.195705	-2.488878
H	-0.229194	1.104985	-2.486269
H	-3.893098	2.027055	0.602969
H	-5.311297	0.005742	0.887418
H	-1.991017	-2.458647	-0.321938
H	-0.584763	-0.481508	-0.577935
H	-5.489533	-2.550053	-0.202425
H	-4.057576	-3.514829	0.242698
H	-5.002013	-2.663129	1.493754

	**TS5**		
O	-3.032093	-0.006712	-0.352534
Br	-1.446978	2.477809	-0.692451
N	-2.633538	-0.839240	1.606028
C	-2.265553	-1.116024	0.206327
C	-1.614195	-0.106094	2.359092
C	-3.206549	-1.964912	2.335357
O	-0.940504	-1.062578	-0.014701
C	-0.453616	-1.727143	-1.182415
C	-0.740731	-0.955099	-2.457786
C	1.038438	-2.006702	-1.007590
C	1.940927	-1.005977	-0.382105
O	1.460993	-3.065965	-1.428225
C	3.249598	-1.418056	-0.077173
C	4.158557	-0.535888	0.485688
C	3.799375	0.796454	0.753744
C	2.498541	1.203637	0.435894
C	1.574175	0.321244	-0.117668
C	4.786733	1.740974	1.365411
H	-3.347218	0.016775	0.794373
H	-2.423433	0.862052	-0.543145
H	-2.729169	-2.050399	-0.148654
H	-1.293468	0.775621	1.785445
H	-0.741076	-0.740922	2.573579
H	-2.067223	0.225312	3.302364
H	-2.439305	-2.725021	2.556869
H	-4.011556	-2.419841	1.74229
H	-3.626642	-1.594784	3.279529
H	-0.926282	-2.722327	-1.237591
H	-0.275375	-1.470333	-3.3108
H	-1.82426	-0.910066	-2.642396
H	-0.357186	0.073426	-2.400268
H	3.535405	-2.450017	-0.292882
H	5.170388	-0.877182	0.723014
H	2.195052	2.237257	0.624972
H	0.572796	0.696906	-0.342565
H	5.713794	1.782891	0.77149
H	4.379254	2.758461	1.444571
H	5.073125	1.407099	2.376414

	**G**		
C	3.896518	1.084154	0.556117
C	4.409075	0.189051	-0.397803
C	3.526440	-0.725966	-0.984191
C	2.179292	-0.757362	-0.631822
C	1.674822	0.136321	0.322588
C	2.555117	1.060586	0.906890
C	0.244156	0.173930	0.740782
C	-0.676573	-0.967534	0.304311
C	5.860274	0.217566	-0.765166
C	-0.388219	-2.220016	1.115137
O	-2.023093	-0.635560	0.512677
H	4.567574	1.809163	1.025851
H	3.902224	-1.427172	-1.734240
H	1.525745	-1.483698	-1.118913
H	2.157596	1.760531	1.645358
H	-0.508364	-1.171279	-0.766401
H	6.168385	1.226594	-1.082391
H	6.487963	-0.048088	0.101434
H	6.086346	-0.485971	-1.578484
H	-1.056992	-3.028890	0.786669
H	0.651269	-2.552740	0.991503
H	-0.569771	-2.026737	2.183397
O	-2.507049	-0.299231	-1.672534
H	-2.312527	0.402101	-2.305649
N	-3.891202	0.564194	0.06243
C	-4.768795	-0.583774	0.115677
H	-5.045132	-0.967289	-0.887974
H	-4.285097	-1.397029	0.672734
H	-5.697337	-0.311116	0.640985
C	-4.479723	1.68441	-0.630143

H	-4.720273	1.47185	-1.694296
H	-5.416674	1.979815	-0.13263
H	-3.797511	2.548926	-0.604388
C	-2.565781	0.274192	-0.382169
H	-1.99867	1.224661	-0.382078
O	-0.182353	1.076499	1.432514

H

C	-2.953814	-0.451133	-1.303607
C	-3.710561	-0.047191	-0.199656
C	-3.025036	0.306475	0.974666
C	-1.639701	0.260438	1.039493
C	-0.893454	-0.149230	-0.074170
C	-1.561234	-0.504648	-1.246407
C	0.591610	-0.228838	0.004230
C	1.249946	-1.000084	1.154862
O	1.166094	-0.762891	-1.100174
C	-5.206786	0.009425	-0.253886
C	0.549410	-2.309379	1.460155
O	2.542722	-1.219563	0.617251
C	2.492778	-1.199220	-0.772041
O	3.443351	-0.390723	-1.296594
H	-3.462936	-0.731826	-2.229803
H	-3.593309	0.625955	1.852816
H	-1.131902	0.553794	1.962068
H	-0.988939	-0.828031	-2.117360
H	1.351919	-0.390417	2.061964
H	-5.573201	1.022205	-0.020809
H	-5.585606	-0.272703	-1.246211
H	-5.654714	-0.669748	0.489745
H	1.190049	-2.903477	2.128010
H	-0.410068	-2.13401	1.96593
H	0.363075	-2.890056	0.544041
H	2.619338	-2.198548	-1.218575
H	3.342099	0.480832	-0.877975
Br	1.284512	1.748622	0.178299

$HNMe_2$

N	0.000000	0.556996	-0.152392
H	0.000000	1.327722	0.513364
C	-1.204689	-0.217930	0.019974
H	-2.090400	0.431407	-0.063047
H	-1.281915	-0.982214	-0.772732
H	-1.269403	-0.754958	0.992622
C	1.204689	-0.217930	0.019974
H	1.281915	-0.982214	-0.772732
H	2.090400	0.431407	-0.063047
H	1.269403	-0.754958	0.992622

TS7

C	3.768611	0.075183	-1.007963
C	4.086121	-0.710188	0.112807
C	3.098400	-0.894865	1.087182
C	1.833390	-0.326454	0.950862
C	1.521799	0.454251	-0.169614
C	2.513103	0.648791	-1.143673
C	0.196343	1.109206	-0.383183
C	-0.900641	0.909937	0.663506
C	5.441687	-1.332654	0.246812
C	-0.791895	1.980745	1.732574
O	-2.170905	1.036725	0.069354
H	4.525247	0.235472	-1.781740
H	3.323821	-1.498095	1.970950
H	1.094835	-0.497888	1.736300
H	2.268433	1.263539	-2.012834
H	-0.785445	-0.084506	1.119606
H	5.560115	-1.843177	1.212797
H	5.613695	-2.071767	-0.553046
H	6.236383	-0.575212	0.154762
H	-1.571498	1.833891	2.494792
H	0.190376	1.946746	2.226023
H	-0.923703	2.976130	1.282226
O	-1.579107	-0.848121	-1.160046
H	-2.352624	-1.598963	-0.425948
N	-3.411305	-1.05856	-0.03065
C	-3.682576	-0.831924	1.379925
H	-4.190472	-1.715947	1.790612
H	-2.745985	-0.683014	1.931407
H	-4.327534	0.049566	1.522536
C	-4.587993	-1.462935	-0.788506
H	-4.98759	-2.399686	-0.375298
H	-5.374141	-0.691192	-0.748314
H	-4.303565	-1.635075	-1.836134
C	-2.543646	-0.00741	-0.775598
H	-3.169764	0.455887	-1.566656
O	0.010304	1.845535	-1.329093

TS6

C	-3.383265	-0.374352	-1.185395
C	-3.856796	0.368826	-0.089366
C	-2.981149	0.620246	0.976726
C	-1.680186	0.136629	0.966014
C	-1.218134	-0.607853	-0.131991
C	-2.083536	-0.848191	-1.215577
C	0.147410	-1.094060	-0.197346
C	1.060591	-1.187725	1.010221
O	0.662723	-1.469841	-1.258960
C	-5.265100	0.868049	-0.068253
C	0.565745	-2.107391	2.104450
O	2.257483	-1.748804	0.471231
C	2.493920	-1.538524	-0.820271
O	2.948723	-0.443256	-1.274592
H	-4.052029	-0.575250	-2.026391
H	-3.327775	1.211886	1.827538
H	-1.018816	0.370697	1.801755
H	-1.722425	-1.419014	-2.073474
H	1.253874	-0.160865	1.373044
H	-5.516313	1.378881	-1.010796
H	-5.968727	0.024786	0.033141
H	-5.439302	1.560001	0.766866
H	1.349801	-2.203912	2.868466
H	-0.330764	-1.695097	2.584341
H	0.332364	-3.103263	1.700967
H	2.789771	-2.44032	-1.373299
H	2.646698	0.435577	-0.743423
Br	1.97872	2.014205	0.076416

2a

C	0.892165	-1.319804	0.101712
O	1.058701	-2.492585	-0.159206
C	-0.462826	-0.707462	0.097557
C	-0.758211	0.518771	0.707932

C	-1.501818	-1.415113	-0.526855
C	-2.056600	1.020463	0.687534
H	0.013192	1.089965	1.228041
C	-2.790935	-0.904738	-0.554372
H	-1.272962	-2.375376	-0.994437
C	-3.093218	0.325835	0.051834
H	-2.271461	1.974376	1.176613
H	-3.585637	-1.466113	-1.054010
C	2.094033	-0.430324	0.429253
H	1.953767	-0.030125	1.450020
C	3.409126	-1.160101	0.338344
H	3.563781	-1.562426	-0.672964
H	4.233809	-0.474116	0.581638
H	3.429438	-1.995971	1.051061
C	-4.486449	0.871902	0.009568
H	-4.793589	1.077628	-1.029039
H	-5.208094	0.145190	0.415652
H	-4.573911	1.805217	0.582823
O	2.060856	0.653901	-0.506565
C	2.370014	1.87006	-0.054548
O	2.410805	2.825942	-0.772197
H	2.57708	1.896753	1.040586

H_2O			
O	0.000000	0.000000	0.120943
H	0.000000	0.752445	-0.483771
H	0.000000	-0.752445	-0.483771

$TMSCF_3$			
Si	-0.856793	0.000045	-0.000201
C	1.083447	0.000149	0.000320
C	-1.364645	-0.126081	1.791989
H	-0.983411	0.728215	2.373982
H	-2.463796	-0.132096	1.877423
H	-0.983361	-1.053520	2.248502
C	-1.362370	-1.489829	-1.005485
H	-2.461359	-1.563356	-1.052776
H	-0.981908	-1.420603	-2.037179
H	-0.978770	-2.420124	-0.556735
C	-1.363403	1.615301	-0.787621
H	-0.980809	2.474217	-0.213333
H	-0.982468	1.691086	-1.818672
H	-2.462459	1.693744	-0.824637
F	1.604029	1.033115	0.697802
F	1.604306	-1.120178	0.546214
F	1.604473	0.087570	-1.242792

DMF			
C	-0.855705	-0.641033	-0.000124
O	-1.944799	-0.094815	-0.000226
H	-0.743823	-1.750513	-0.000158
N	0.342573	-0.018272	0.000041
C	0.422205	1.419324	0.000105
H	0.957769	1.783200	0.893312
H	0.957953	1.783272	-0.892962
H	-0.597002	1.825517	0.000015
C	1.576798	-0.757788	0.000149
H	1.364362	-1.836254	0.000104
H	2.180759	-0.520892	-0.892381
H	2.180575	-0.520932	0.892814

DMF and $TMSCF_3$ complex			
C	0.521025	-0.610682	1.537040
C	-2.728781	-0.152142	-0.070698
O	-2.045701	0.862344	-0.082090
N	-4.071589	-0.192784	0.002544
C	-4.787150	-1.442624	0.013432
C	-4.843896	1.021454	0.076734
Si	1.189672	0.197128	-0.009522
C	1.110390	2.061315	0.032884
C	3.086158	-0.231256	0.019518
C	0.582427	-0.539917	-1.615118
H	-2.278643	-1.169110	-0.122791
H	-4.076721	-2.279065	-0.043804
H	-5.477067	-1.508622	-0.844558
H	-5.379162	-1.548372	0.937988
H	-5.436155	1.050449	1.006514
H	-5.538784	1.093878	-0.776487
H	-4.157711	1.876980	0.058101
H	1.612488	2.503932	-0.842244
H	1.584854	2.461630	0.943127
H	0.053417	2.368923	0.023737
H	1.206504	-0.198283	-2.456867
H	-0.452946	-0.219054	-1.802104
H	0.612972	-1.640840	-1.590256
H	-0.516971	-0.290256	1.708964
H	0.542559	-1.709373	1.459506
H	1.12059	-0.31728	2.414043
F	3.761796	0.293242	-1.027867
F	3.713044	0.222865	1.128309
F	3.319723	-1.562938	-0.018596

H_2O and $TMSCF_3$ complex,			
C	-0.565824	-0.000008	-0.104710
H	-3.357899	-0.749021	0.349306
F	-0.993133	-0.000107	-1.379802
F	-1.133591	-1.080143	0.484569
F	-1.133590	1.080218	0.484403
Si	1.371823	0.000004	0.048182
C	1.938037	1.556792	-0.811201
H	3.037183	1.633997	-0.774473
H	1.632810	1.558643	-1.869834
H	1.518088	2.453221	-0.327601
C	1.728417	0.000154	1.879873
H	1.305293	-0.893029	2.366705
H	2.816625	0.000169	2.056219
H	1.305291	0.893415	2.366559
C	1.938034	-1.556926	-0.810945
H	3.037180	-1.634126	-0.774207
H	1.518087	-2.453275	-0.327193
H	1.632803	-1.558954	-1.869576
O	-3.897780	0.000009	0.069957
H	-3.357895	0.749053	0.349253

$TS3_{TMSCF3}$			
Si	3.018534	-0.995407	0.562244
C	1.526242	-0.540032	1.583989
H	1.758030	-0.552647	2.660967
H	1.162597	0.466327	1.323095
H	0.719298	-1.265964	1.390237
C	3.818371	-2.585187	1.124655
H	3.123852	-3.427236	0.968620

H	4.738770	-2.793794	0.556183
H	4.072409	-2.548404	2.196044
C	2.696730	-0.959213	-1.274179
H	2.348709	0.034947	-1.595754
H	3.607127	-1.206752	-1.843195
H	1.915809	-1.697422	-1.520445
C	4.316772	0.410364	0.888411
F	4.659946	0.520035	2.189276
F	5.469660	0.229099	0.209951
F	3.859222	1.631827	0.521068
C	-0.069607	2.153313	-1.176497
O	-1.034073	1.602059	-1.750103
H	0.743292	1.556399	-0.725532
N	0.071875	3.458363	-1.059286
C	-0.910111	4.378234	-1.590043
H	-1.298825	5.014478	-0.780300
H	-0.446306	5.022974	-2.352484
H	-1.734057	3.813767	-2.040444
C	1.224074	4.025626	-0.39248
H	1.892749	3.224687	-0.052673
H	1.773463	4.682338	-1.084396
H	0.901827	4.620592	0.475914
C	-0.955383	-0.337629	-1.658499
H	-0.233692	-0.276779	-0.850342
C	-0.504527	-0.545515	-3.062958
H	0.531141	-0.208895	-3.196884
H	-0.5672	-1.608405	-3.326379
H	-1.167834	0.007764	-3.740857
C	-2.432998	-0.432587	-1.366758
O	-3.200139	-0.623958	-2.290095
C	-2.933116	-0.15629	0.010613
C	-2.106773	0.201286	1.083957
C	-4.313688	-0.225347	0.2267
C	-2.649282	0.477904	2.332488
H	-1.026227	0.260942	0.961063
C	-4.853761	0.04663	1.479631
H	-4.958814	-0.49903	-0.610921
C	-4.032786	0.404842	2.556725
H	-1.984991	0.754242	3.15645
H	-5.935296	-0.018228	1.628795
C	-4.603672	0.709678	3.907615
H	-4.42005	1.761111	4.183983
H	-5.688274	0.534796	3.937047
H	-4.131849	0.089749	4.686671
Br	-0.850941	-2.783553	-0.76749

TS3$_{STMSCF3}$

C	-3.089806	-1.382483	-0.631556
H	0.415118	-2.467233	1.438340
F	-3.698223	-1.353082	-1.826272
F	-1.762803	-1.629783	-0.912285
F	-3.528179	-2.482722	0.000750
Si	-3.316319	0.251492	0.396813
C	-5.158445	0.441950	0.622622
H	-5.375343	1.348921	1.210764
H	-5.671267	0.535833	-0.347833
H	-5.584905	-0.420451	1.159464
C	-2.422016	-0.053525	2.006066
H	-1.368451	-0.324695	1.843467
H	-2.441330	0.854877	2.630253
H	-2.905654	-0.865289	2.572731
C	-2.564893	1.593649	-0.656482
H	-2.645269	2.566665	-0.145921
H	-1.500521	1.398028	-0.858410
H	-3.093478	1.671054	-1.620247
O	0.339636	-2.757925	0.503133
H	-0.489554	-2.371373	0.158500
Br	4.238632	-0.658147	-0.040243
C	1.804815	-1.811889	-0.119335
H	2.470784	-2.566388	0.293320
C	1.526582	-1.867022	-1.582687
H	0.651916	-1.265995	-1.862627
H	2.401167	-1.49931	-2.13364
H	1.363948	-2.912827	-1.878258
C	1.60735	-0.637833	0.828112
O	1.451079	-0.949566	2.007466
C	1.298994	0.746409	0.375128
C	0.729481	1.603548	1.327795
C	1.517556	1.244111	-0.918347
C	0.370889	2.905014	0.997622
H	0.571004	1.22612	2.339317
C	1.146646	2.542257	-1.245971
H	2.01833	0.634384	-1.667911
C	0.560149	3.396531	-0.300017
H	-0.074248	3.551805	1.75888
H	1.325653	2.909104	-2.260508
C	0.112979	4.773135	-0.679831
H	0.76798	5.215582	-1.444861
H	-0.905087	4.73842	-1.105628
H	0.080996	5.444106	0.1907

TS3$_{CuBr}$

C	0.910233	-0.466211	-0.094917
O	0.122037	-0.499502	-1.060130
C	2.167149	0.304036	-0.233282
C	3.064896	0.521620	0.824427
C	2.449366	0.876492	-1.479965
C	4.207348	1.283158	0.628626
H	2.890291	0.086854	1.809321
C	3.601403	1.631533	-1.671303
H	1.751198	0.717195	-2.303555
C	4.503736	1.848024	-0.622971
H	4.895696	1.440079	1.463736
H	3.806378	2.063392	-2.654641
C	0.438049	-0.902958	1.249743
H	1.131045	-0.816764	2.081076
C	-0.750426	-1.793706	1.425506
H	-1.060148	-2.249530	0.473991
H	-0.504892	-2.598745	2.129271
H	-1.596314	-1.228258	1.840085
C	5.751418	2.651557	-0.817571
H	5.780298	3.509260	-0.126231
H	6.645915	2.043385	-0.605508
H	5.831355	3.033149	-1.844923
Br	1.990286	-2.837861	0.424735
Br	-3.999214	-0.158057	-0.725688
Cu	-1.805546	-0.604764	-1.060371
C	-0.835498	1.561526	0.928978
O	-0.365552	0.735854	1.752861
H	-0.418731	1.673005	-0.085005
N	-1.829528	2.375243	1.20749
C	-2.529831	2.318928	2.471129

H	-2.574014	3.324261	2.915082
H	-3.556252	1.956115	2.304525
H	-2.007269	1.638618	3.152236
C	-2.339379	3.287289	0.207208
H	-1.713676	3.241157	-0.692983
H	-3.368485	3.001641	-0.059164
H	-2.335058	4.315248	0.598857

TS3s$_{CuBr}$			
C	-0.448612	0.399572	-0.197684
O	0.285712	0.216117	-1.185787
C	-1.705051	-0.385666	-0.082560
C	-2.648862	-0.208120	0.941045
C	-1.924243	-1.397451	-1.027367
C	-3.774060	-1.018491	1.007313
H	-2.529806	0.590059	1.675676
C	-3.054381	-2.203461	-0.956233
H	-1.186646	-1.547626	-1.817683
C	-4.003725	-2.027729	0.059053
H	-4.500862	-0.860557	1.808768
H	-3.202710	-2.989833	-1.701461
C	0.110126	1.062037	1.032030
H	-0.567276	1.205707	1.871797
C	1.321037	1.935598	0.982421
H	1.761919	1.966359	-0.024317
H	1.039146	2.955543	1.269547
H	2.077470	1.588689	1.700245
C	-5.241252	-2.867875	0.115395
H	-5.584712	-3.012580	1.150139
H	-6.063299	-2.377383	-0.434078
H	-5.082481	-3.852842	-0.346991
Br	-1.454213	2.806692	-0.216320
Br	3.663294	-1.21498	0.39329
Cu	2.194804	-0.184853	-1.055466
O	0.854318	-0.444582	1.803798
H	1.712353	-0.709559	1.359039
H	0.25365	-1.207923	1.719332

The 3D images of calculation structures by CYLView program.

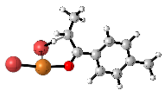
TS1

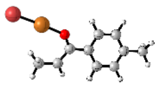
A

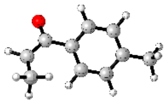
B

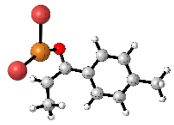
C

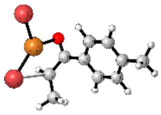
TS2

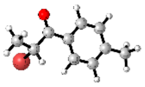
D

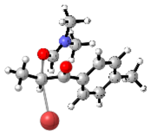
TS3

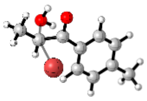
TS3s

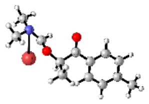
E

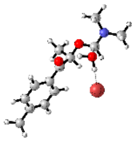
TS4

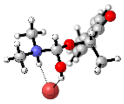
F

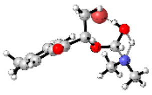
TS5

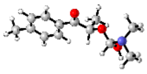
G

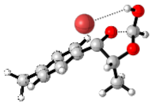
H

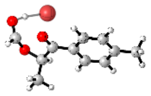
TS6

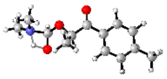
TS7

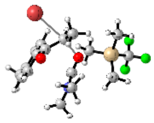
TS3$_{TMSCF3}$

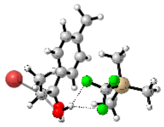
TS3s$_{TMSCF3}$

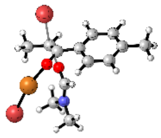
TS3$_{CuBr}$

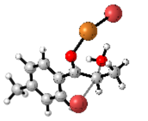
TS3s$_{CuBr}$

S2. The condensed Fukui functions and RDG of DMF, H_2O, and their complexes with $TMSCF_3$

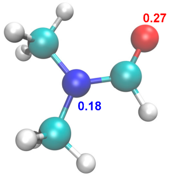

The condensed fukui functions
DMF

atom	N-1	N	f-(condensed fukui function)
Atom 1(C):	0.21329038	0.12848997	0.08480041
Atom 2(O):	-0.07896361	-0.34512971	0.2661661
Atom 3(H):	0.09304016	0.04284138	0.05019878
Atom 4(N):	0.15665833	-0.02844107	0.1850994
Atom 5(C):	0.0131058	-0.03468287	0.04778867
Atom 6(H):	0.10234211	0.04264516	0.05969695
Atom 7(H):	0.10234261	0.0426449	0.05969771
Atom 8(H):	0.0821212	0.03933969	0.04278151
Atom 9(C):	0.02018657	-0.02646449	0.04665106
Atom 10(H):	0.08590065	0.05259033	0.03331032
Atom 11(H):	0.10498825	0.04308307	0.06190518
Atom 12(H):	0.10498754	0.04308365	0.06190389

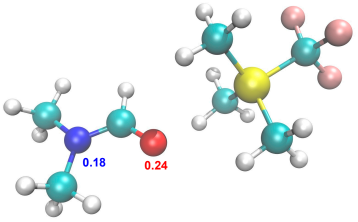

The condensed fukui functions
DMF and $TMSCF_3$ complex

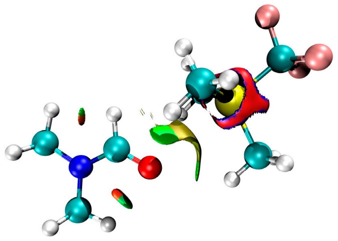

The RDG of DMF and $TMSCF_3$ complex

atom	N-1	N	f-(condensed fukui function)
Atom 1(C):	-0.18494526	-0.18404977	-0.00089549
Atom 2(C):	0.21435029	0.14118759	0.0731627
Atom 3(O):	-0.05566351	-0.29607638	0.24041287
Atom 4(N):	0.15651645	-0.02173067	0.17824712
Atom 5(C):	0.02112313	-0.02413408	0.04525721
Atom 6(C):	0.01374085	-0.03238394	0.04612479
Atom 7(Si):	0.3390656	0.34681792	-0.00775232
Atom 8(C):	-0.18193876	-0.18436331	0.00242455
Atom 9(C):	0.19078319	0.19711489	-0.0063317
Atom 10(C):	-0.18487612	-0.18430783	-0.00056829
Atom 11(H):	0.09068152	0.04796468	0.04271684
Atom 12(H):	0.08684547	0.05428388	0.03256159
Atom 13(H):	0.10552236	0.04521547	0.06030689
Atom 14(H):	0.10544144	0.04525009	0.06019135
Atom 15(H):	0.10277578	0.04485908	0.0579167
Atom 16(H):	0.10275585	0.0449025	0.05785335
Atom 17(H):	0.08195904	0.04096887	0.04099017
Atom 18(H):	0.0503744	0.03832827	0.01204613
Atom 19(H):	0.05000018	0.03830258	0.0116976
Atom 20(H):	0.02634872	0.03491316	-0.00856444
Atom 21(H):	0.05576805	0.03804287	0.01772518
Atom 22(H):	0.01490385	0.03352422	-0.01862037
Atom 23(H):	0.04175468	0.0385694	0.00318528
Atom 24(H):	0.01369481	0.03395092	-0.02025611

Atom 25(H):	0.04169516	0.0387748	0.00292036
Atom 26(H):	0.05565648	0.03803996	0.01761652
Atom 27(F):	-0.11674919	-0.13770394	0.02095475
Atom 28(F):	-0.1169481	-0.13758538	0.02063728
Atom 29(F):	-0.12063634	-0.13867585	0.01803951

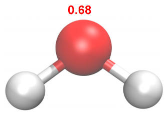

The condensed fukui functions
H_2O

atom	N-1	N	f-(condensed fukui function)
Atom 1 (O):	0.341656	-0.33732	0.678977
Atom 2 (H):	0.329172	0.16866	0.160512
Atom 3 (H):	0.329172	0.16866	0.160512

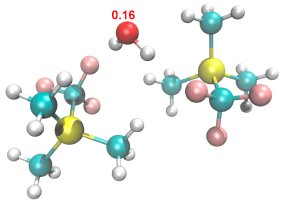

The condensed fukui functions
H_2O and 2TMSCF_3 complex

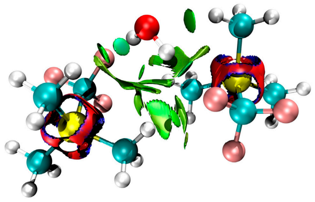

The RDG of H_2O and 2TMSCF_3 complex

atom	N-1	N	f-(condensed fukui function)
Atom 1(O):	-0.14761634	-0.30862058	0.16100424
Atom 2(H):	0.14940257	0.12608548	0.02331709
Atom 3(H):	0.14941636	0.12612159	0.02329477
Atom 4(F):	-0.07602399	-0.10265715	0.02663316
Atom 5(F):	-0.07613866	-0.10257716	0.0264385
Atom 6(C):	0.21953281	0.20660629	0.01292652
Atom 7(C):	0.21939939	0.20661957	0.01277982
Atom 8(F):	-0.08816286	-0.12322816	0.0350653
Atom 9(F):	-0.07908015	-0.12565172	0.04657157
Atom 10(Si):	0.38443065	0.35525024	0.02918041
Atom 11(C):	-0.15352158	-0.18343208	0.0299105
Atom 12(H):	0.06522951	0.0452533	0.01997621
Atom 13(H):	0.04727273	0.02836841	0.01890432
Atom 14(H):	0.06575539	0.04202619	0.0237292
Atom 15(C):	-0.1576829	-0.18203258	0.02434968
Atom 16(H):	0.02898711	0.02851668	0.00047043
Atom 17(H):	0.06943807	0.04606808	0.02336999
Atom 18(H):	0.05905281	0.04179189	0.01726092
Atom 19(C):	-0.15084266	-0.17928441	0.02844175
Atom 20(H):	0.06539753	0.04582429	0.01957324
Atom 21(H):	0.06242717	0.04186747	0.0205597
Atom 22(H):	0.06355039	0.04278878	0.02076161
Atom 23(F):	-0.07920828	-0.12564562	0.04643734
Atom 24(F):	-0.08829891	-0.12322728	0.03492837
Atom 25(Si):	0.3841225	0.3552754	0.0288471
Atom 26(C):	-0.1538057	-0.18339978	0.02959408
Atom 27(H):	0.0651142	0.04526547	0.01984873
Atom 28(H):	0.0471215	0.02840787	0.01871363
Atom 29(H):	0.06562136	0.04203502	0.02358634
Atom 30(C):	-0.15107029	-0.17929462	0.02822433
Atom 31(H):	0.06526998	0.0458183	0.01945168
Atom 32(H):	0.06232348	0.04185847	0.02046501
Atom 33(H):	0.06339787	0.04278403	0.02061384
Atom 34(C):	-0.15791861	-0.1820108	0.02409219
Atom 35(H):	0.0588682	0.04179263	0.01707557

Atom 36(H):	0.02889874	0.02856026	0.00033848
Atom 37(H):	0.06934064	0.04607623	0.02326441

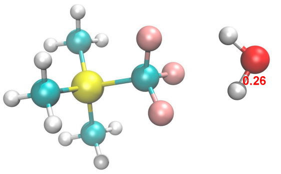

The condensed fukui functions H_2O and $TMSCF_3$ complex

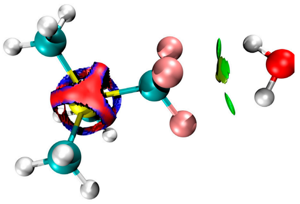

The RDG of H_2O and $TMSCF_3$ complex

atom	N-1	N	f-(condensed fukui function)
Atom 1(C):	0.28114852	0.2059938	0.07515472
Atom 2(H):	0.18381248	0.13854467	0.04526781
Atom 3(F):	-0.02566612	-0.12338436	0.09771824
Atom 4(F):	-0.03433178	-0.11399467	0.07966289
Atom 5(F):	-0.03433084	-0.11399431	0.07966347
Atom 6(Si):	0.40246523	0.3582857	0.04417953
Atom 7(C):	-0.15730758	-0.17902929	0.02172171
Atom 8(H):	0.07848076	0.04594198	0.03253878
Atom 9(H):	0.06089648	0.04264797	0.01824851
Atom 10(H):	0.0596373	0.04255709	0.01708021
Atom 11(C):	-0.15600572	-0.17906807	0.02306235
Atom 12(H):	0.05962821	0.04253562	0.01709259
Atom 13(H):	0.0795906	0.04599769	0.03359291
Atom 14(H):	0.05962823	0.04253551	0.01709272
Atom 15(C):	-0.15730698	-0.17902966	0.02172268
Atom 16(H):	0.07848104	0.04594199	0.03253905
Atom 17(H):	0.05963735	0.04255702	0.01708033
Atom 18(H):	0.0608966	0.04264785	0.01824875
Atom 19(O):	-0.08316551	-0.34623074	0.26306523
Atom 20(H):	0.18381174	0.13854421	0.04526753

S3. Comparison Experiments of Trimethylsilyl Derivatives

Table S1. Optimization of reaction conditions [a].

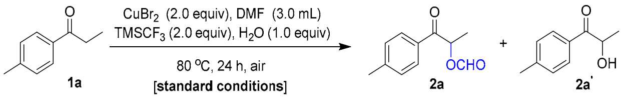

Entry	Conditions	2a(%) [b]	2a′ (%) [b]
1	$TMSCF_3$	95	- [c]
2	Without $TMSCF_3$	65	23
3	TMS[d] instead of $TMSCF_3$	70	8
4	TMSI[e] instead of $TMSCF_3$	65	14
5	TMCS[f] instead of $TMSCF_3$	66	16
6	TMSA[g] instead of $TMSCF_3$	71	15
7	TMSDEA[h] instead of $TMSCF_3$	69	20

[a] Reaction conditions: **1a** (1.0 mmol), metal catalyst (2.0 mmol), $TMSCF_3$ (2.0 mmol), H_2O (1.0 mmol), DMF (3 mL), 80 °C, 24 h. [b] Isolated yields. [c] Not detected. [d] TMS: Tetramethylsilane. [e] TMSI: Iodotrimethylsilane. [f] TMCS: Chlorotrimethylsilane. [g] TMSA: Azidotrimethylsilane. [h] TMSDEA: N-(Trimethylsilyl)diethylamine.

S4. General Procedure for Control Experiments Processes

(1) XRD studies

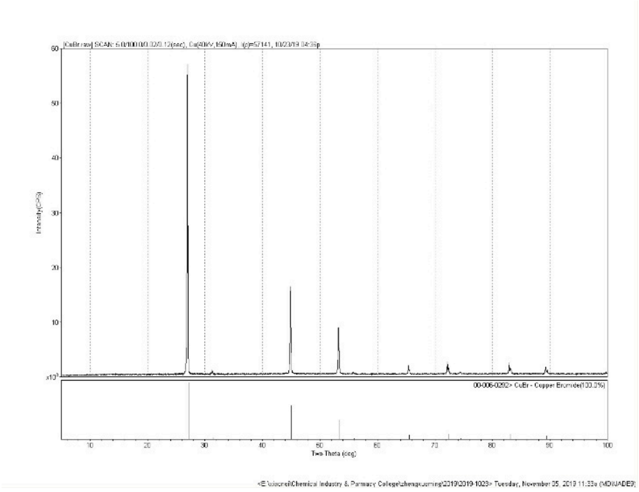

Figure S1. XRD studies of the white solid.

S5. ORTEP Drawing and Crystal Data for Compound 4a

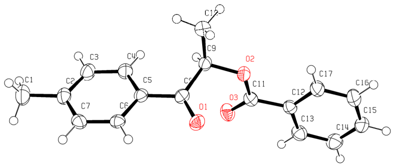

Figure S2. An ORTEP showing the crystal structure of **4a** with displacement ellipsoids drawn at the 50% probability level.

Solvent and method for the crystal growth: The crystal was grown using EtOAc and hexane (2:1) as a solvent by slow evaporation at room temperature. A needle shaped single crystal was mounted on a loop with applying small amount of a paraffin oil. A suitable crystal was selected and was transferred to a 'Rigaku Saturn 70 CCD' diffractometer. The crystal was kept at 123.15 K during data

collection. Using Olex2[1], the structure was solved with the ShelXT[2] structure solution program using Intrinsic Phasing and refined with the ShelXL refinement package using Least Squares minimisation.

Table S2. Crystal data and structure refinement for **4a.**

Identification code	**4a**
Empirical formula	$C_{17}H_{16}O_3$
Formula weight	268.30
Temperature/K	123.15
Crystal system	monoclinic
Space group	$P2_1/c$
a/Å	20.4570(9)
b/Å	9.1734(4)
c/Å	16.0710(7)
α/°	90
β/°	109.656(5)
γ/°	90
Volume/Å^3	2840.1(2)
Z	8
ρ_{calc}g/cm^3	1.255
μ/mm^{-1}	0.085
F(000)	1136.0
Crystal size/mm^3	0.2 × 0.18 × 0.16
Radiation	MoKα (λ = 0.71073)
Theta range for data collection/°	4.228 to 52.744
Index ranges	-25 ≤ h ≤ 25, -11 ≤ k ≤ 11, -20 ≤ l ≤ 20
Reflections collected	29584
Independent reflections	5809 [R_{int} = 0.0506, R_{sigma} = 0.0337]
Data/restraints/parameters	5809/0/365
Goodness-of-fit on F^2	1.023
Final R indexes [I>=2σ (I)]	R1 = 0.0443, wR2 = 0.1048
Final R indexes [all data]	R1 = 0.0570, wR2 = 0.1143
Largest diff. peak/hole / e Å^{-3}	0.21/-0.23

S6. Rectification of $TMSCF_3$

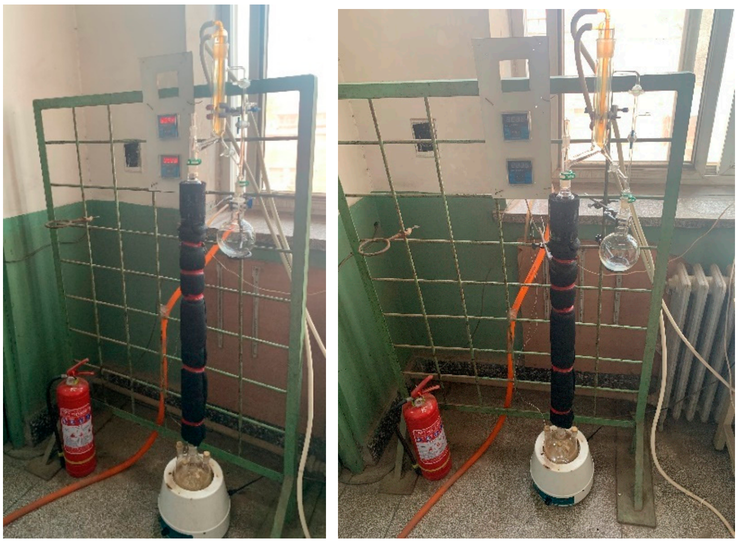

Figure S3. Rectification tower of $TMSCF_3$.

S7. Reference

1. Dolomanov, O.V.; Bourhis, L.J.; Gildea, R.J.; Howard, J.A.K.; Puschmann, H. OLEX2: A complete structure solution, refinement and analysis program. *J. Appl. Crystallogr*. **2009**, *42*, 339–341.
2. Sheldrick, G. Crystal structure refinement with SHELXL. *Acta Crystallogr. Sect. C* **2015**, *71*, 3–8.

S8. NMR and IR Spectra

^1H NMR (500 MHz, $CDCl_3$) of 2a

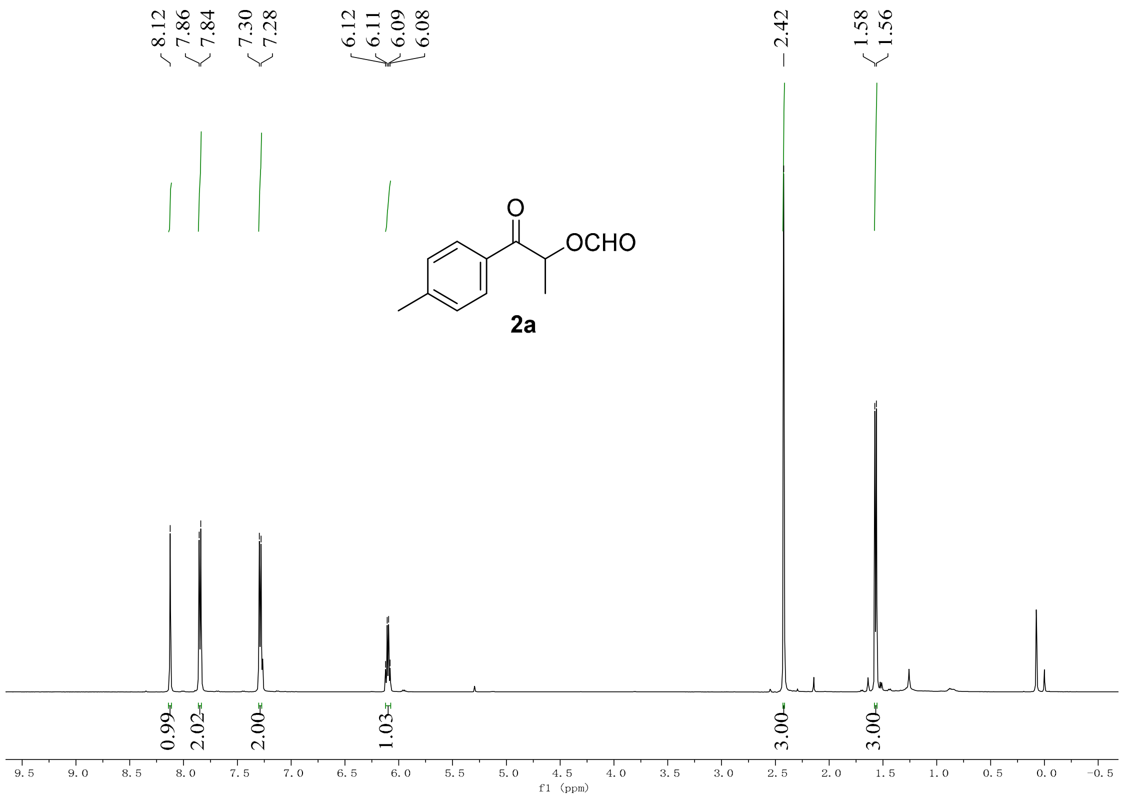

^{13}C NMR (126 MHz, $CDCl_3$) of 2a

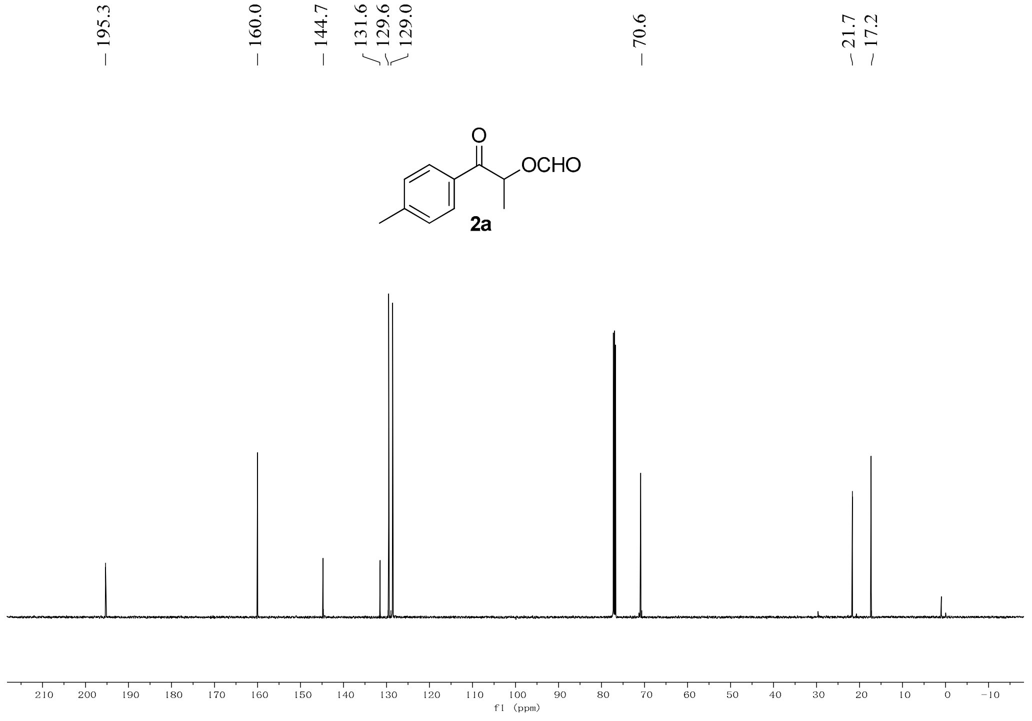

IR(KBr) of 2a

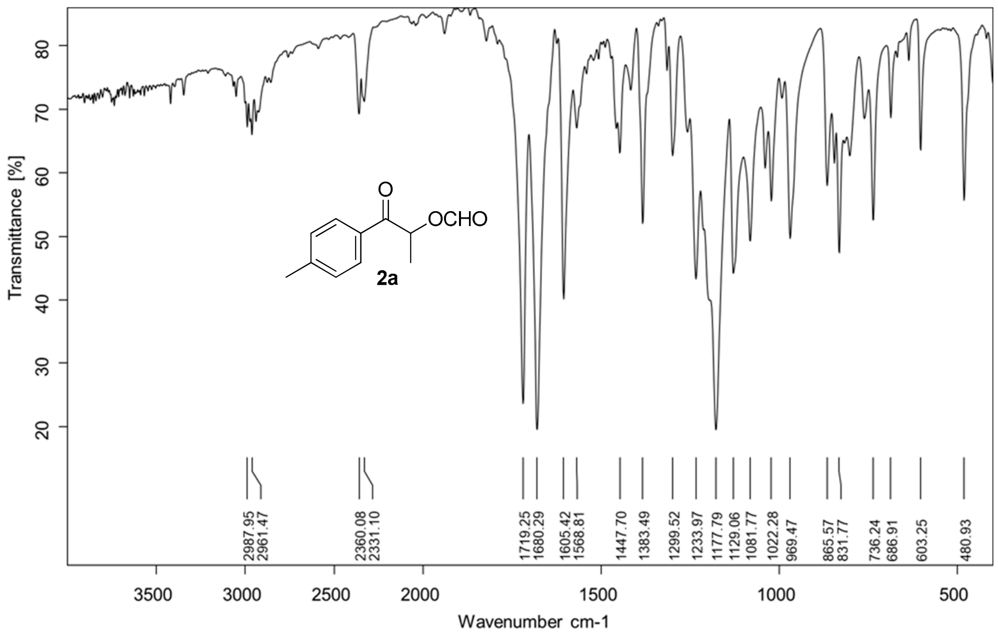

¹H NMR (500 MHz, CDCl₃) of 2b

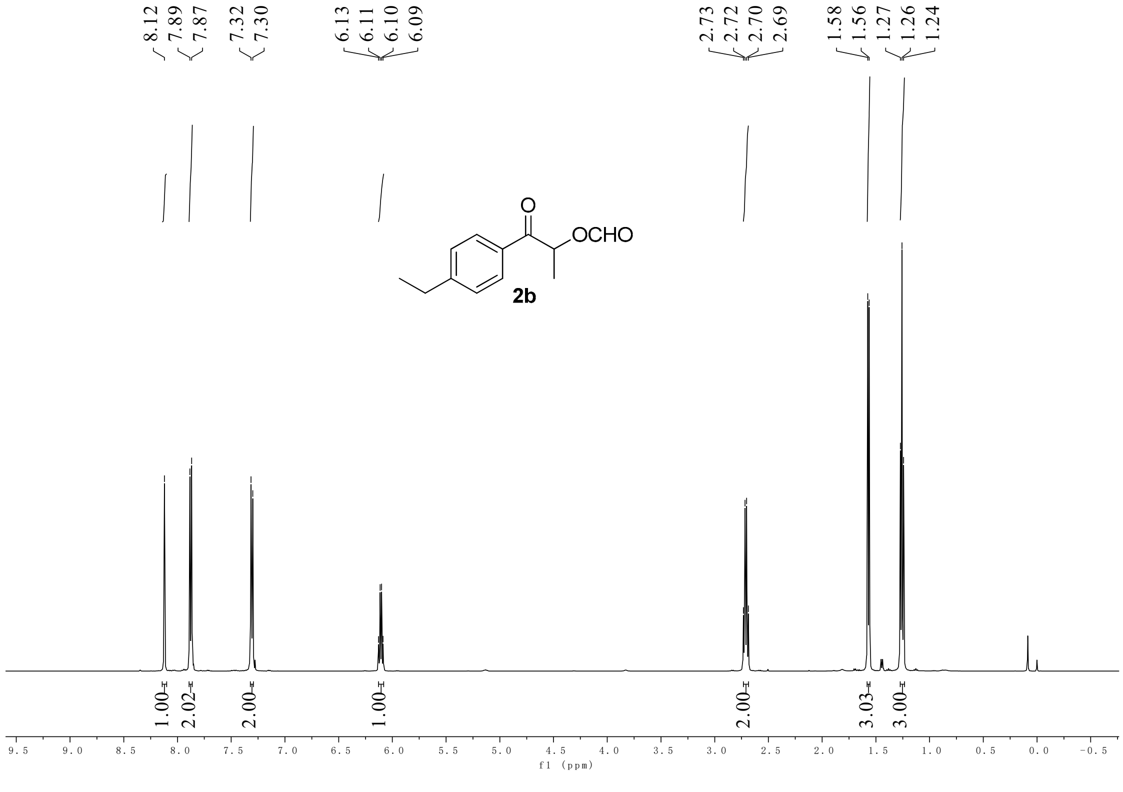

¹³C NMR (126 MHz, CDCl₃) of 2b

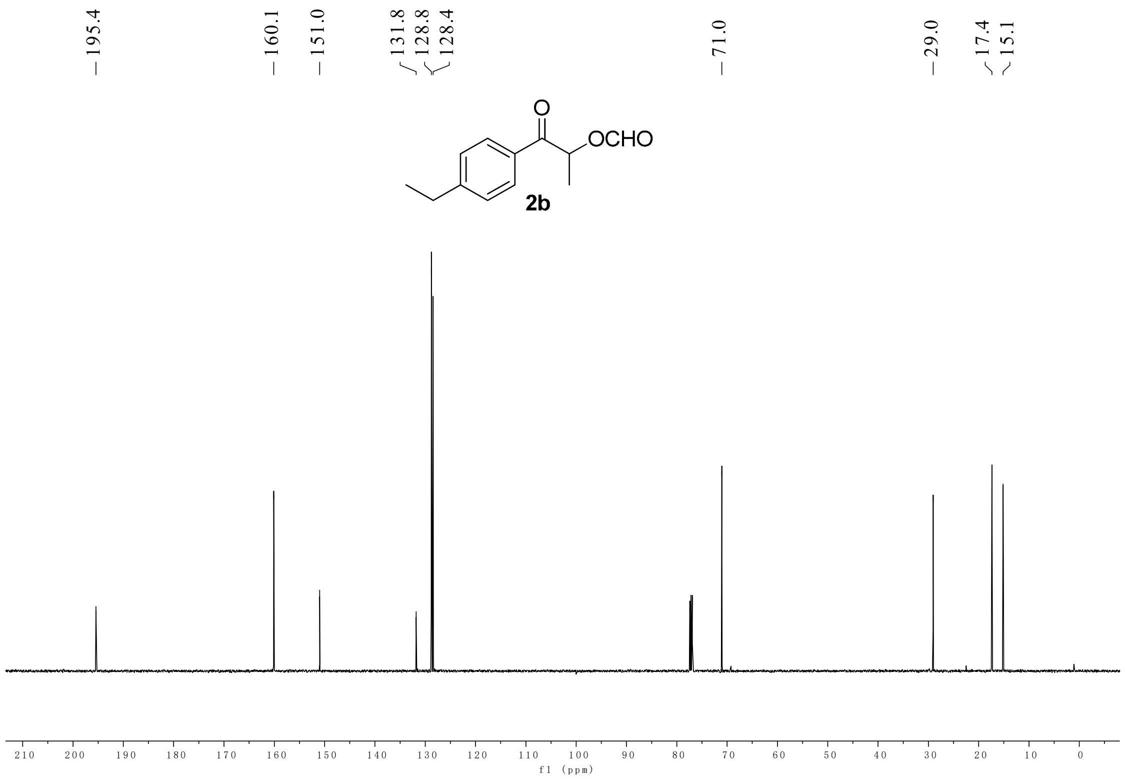

IR(KBr) of 2b

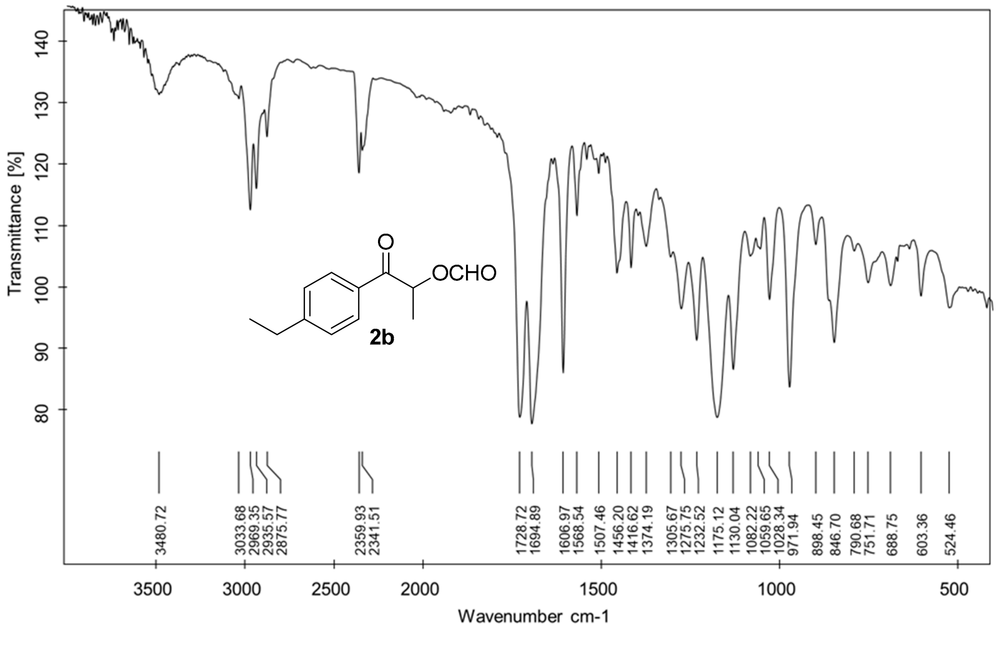

^1H NMR (500 MHz, $CDCl_3$) of 2c

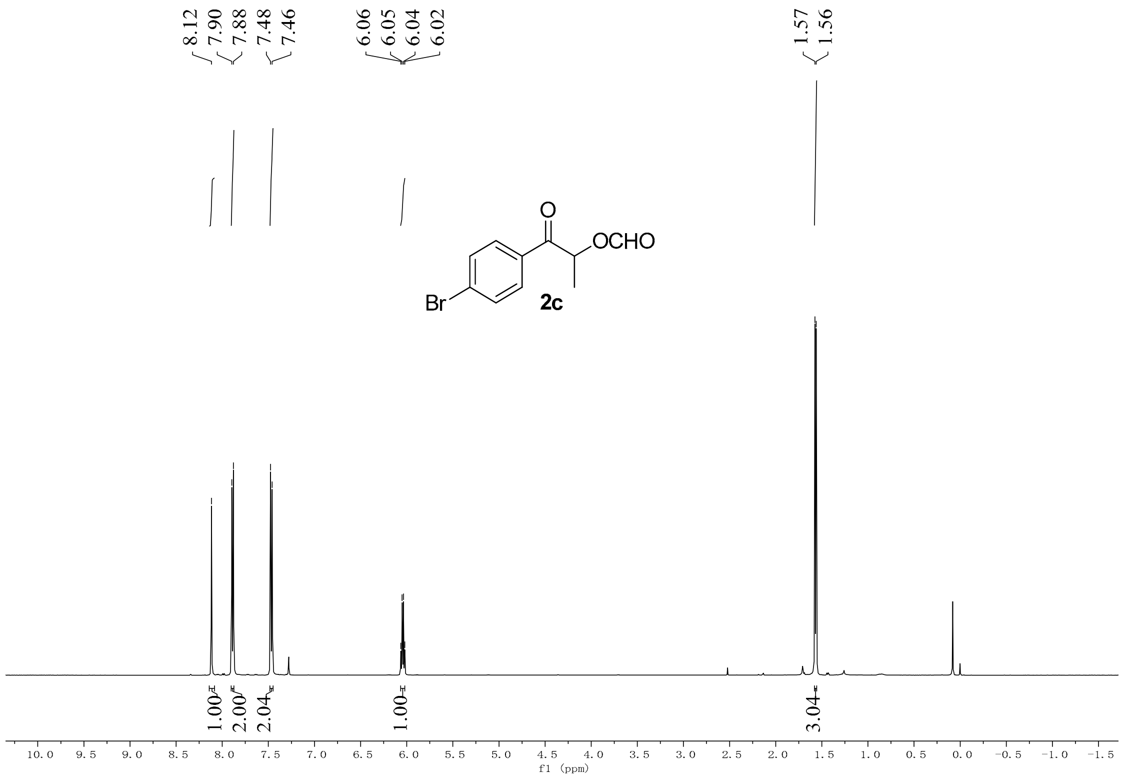

^{13}C NMR (126 MHz, $CDCl_3$) of 2c

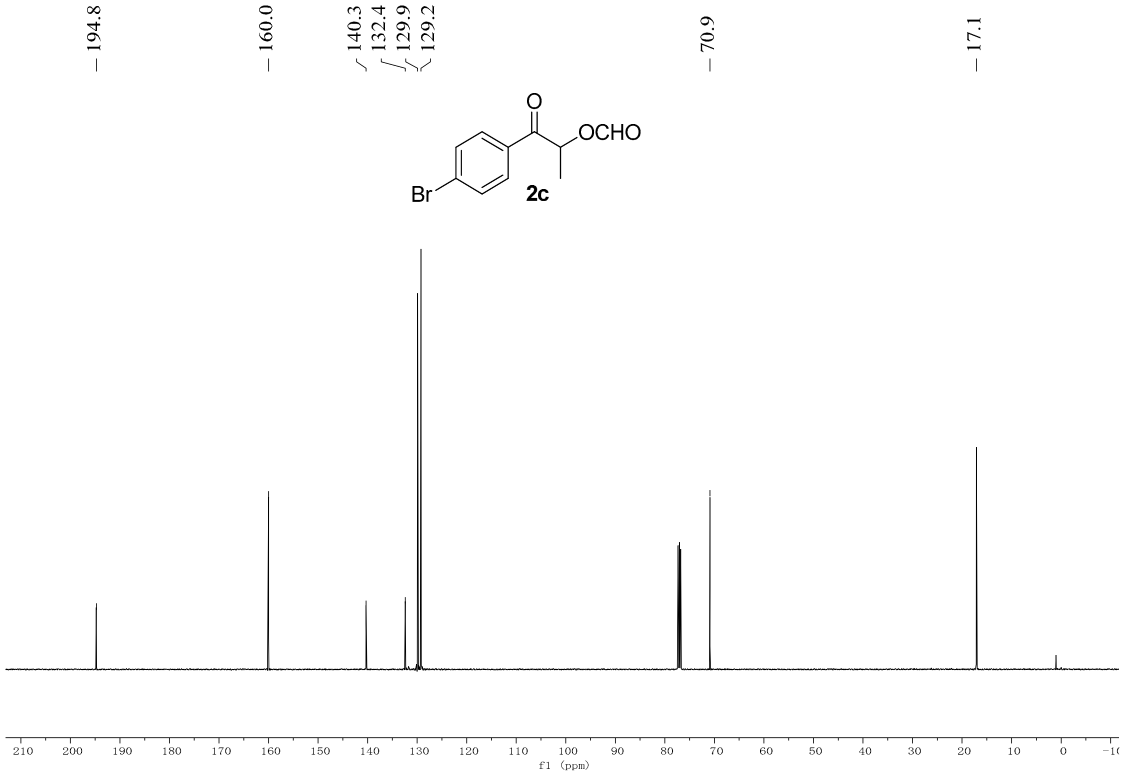

^{1}H NMR (500 MHz, $CDCl_3$) of 2d

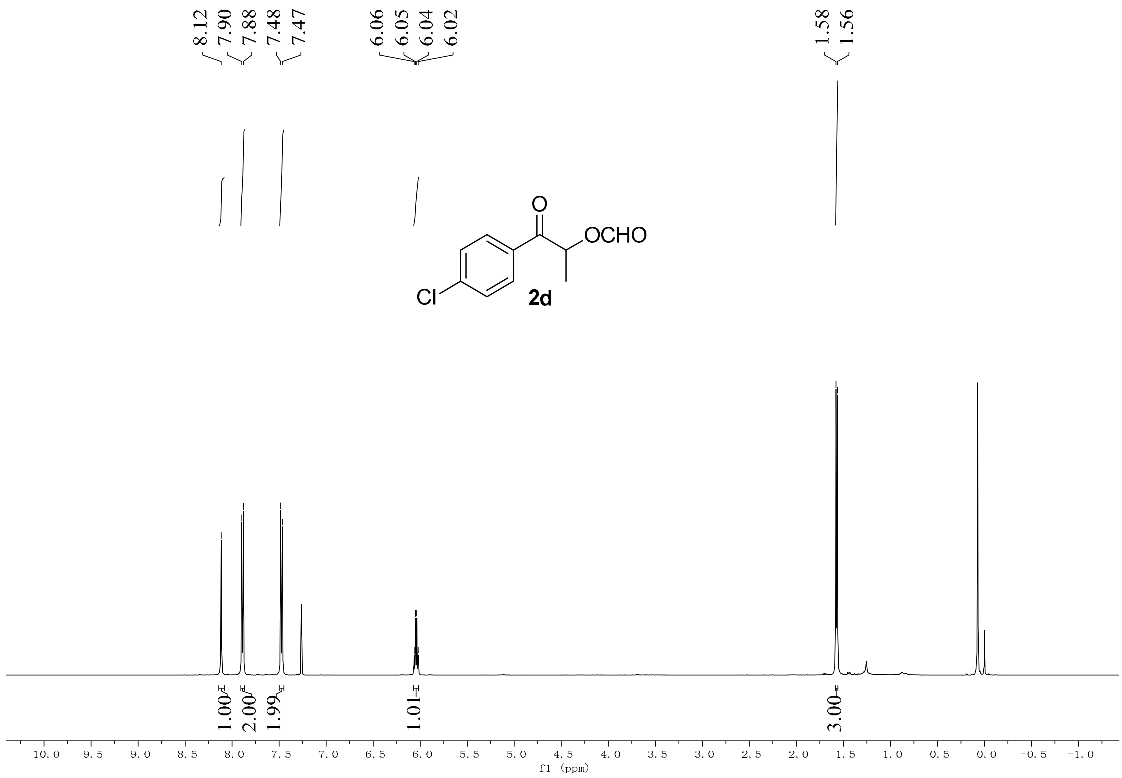

^{13}C NMR (126 MHz, $CDCl_3$) of 2d

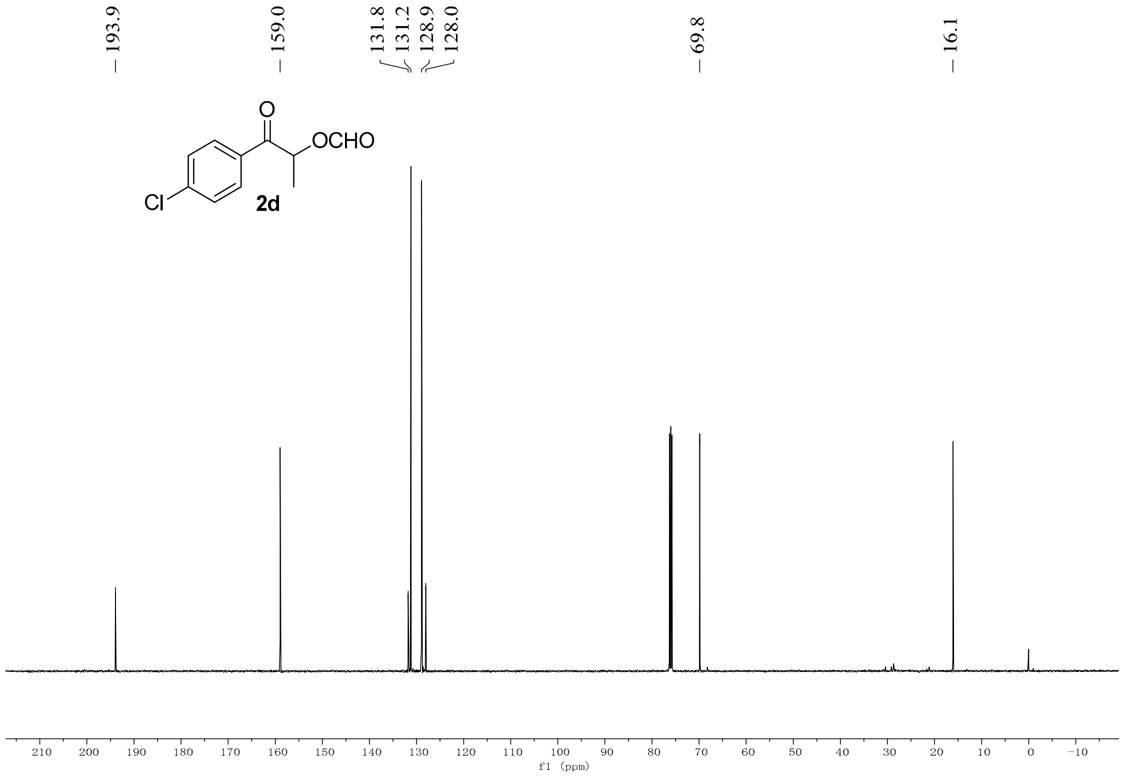

IR(KBr) of 2d

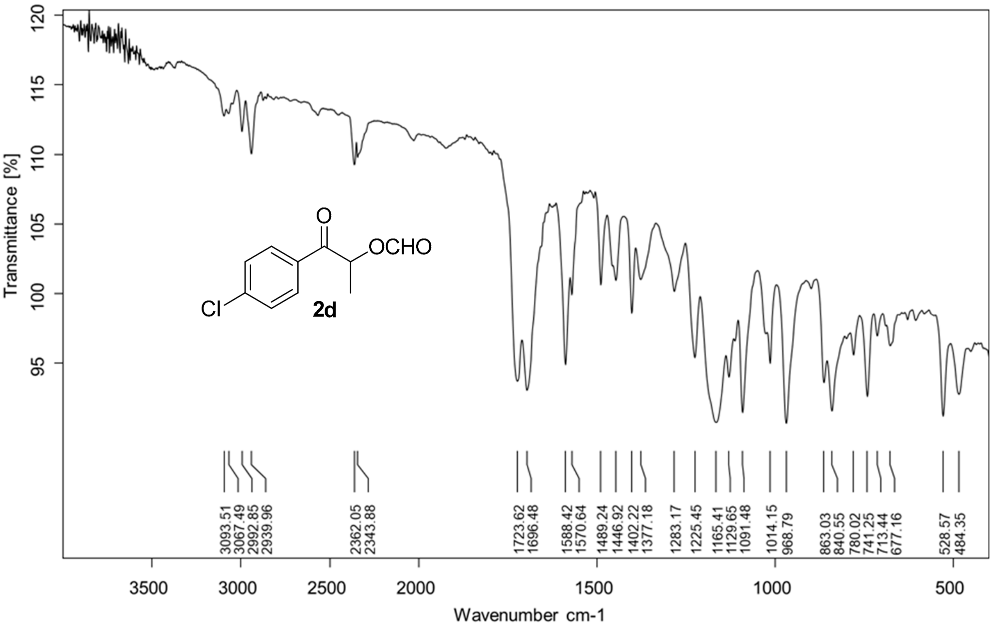

^{1}H NMR (500 MHz, $CDCl_3$) of 2e

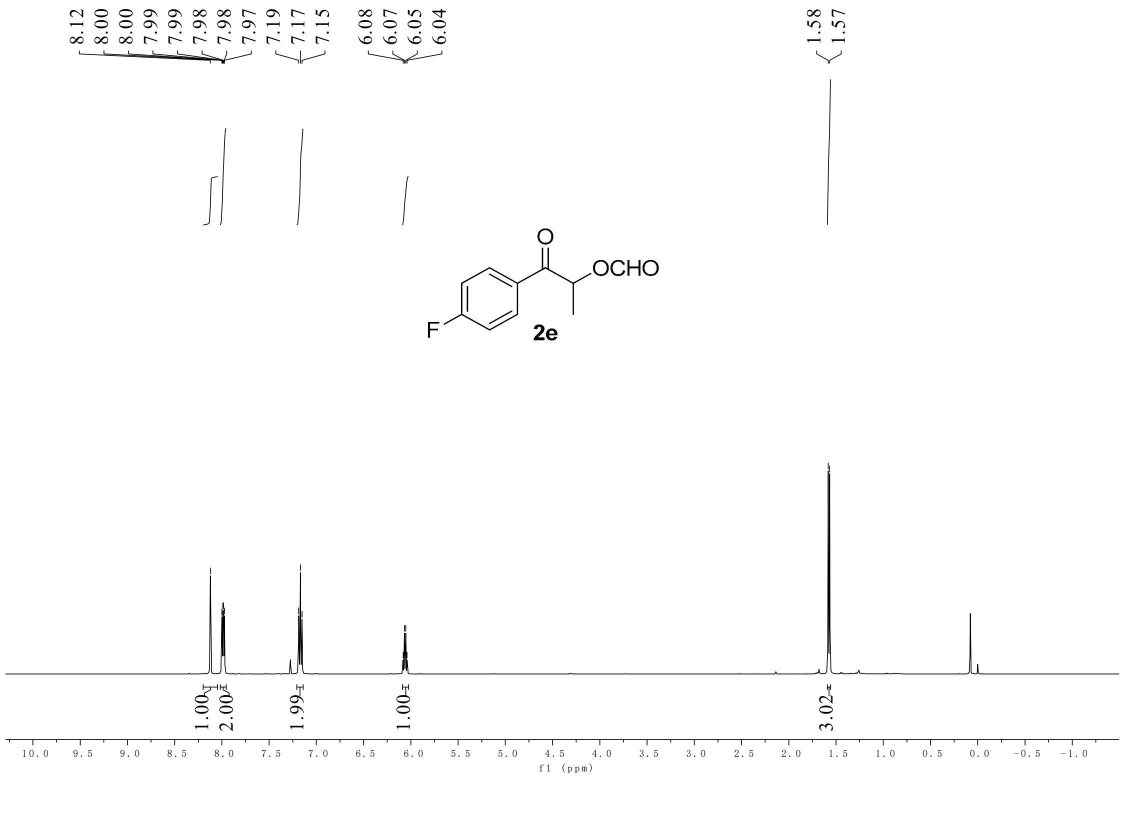

^{13}C NMR (126 MHz, $CDCl_3$) of 2e

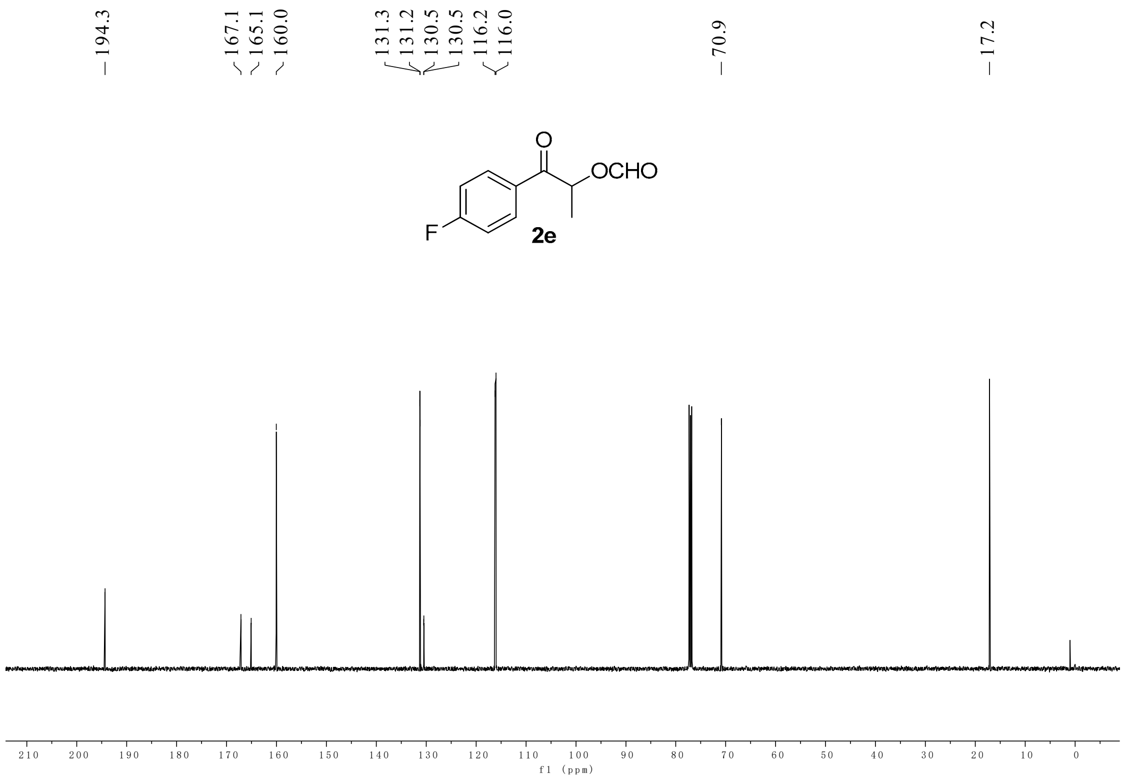

IR(KBr) of 2e

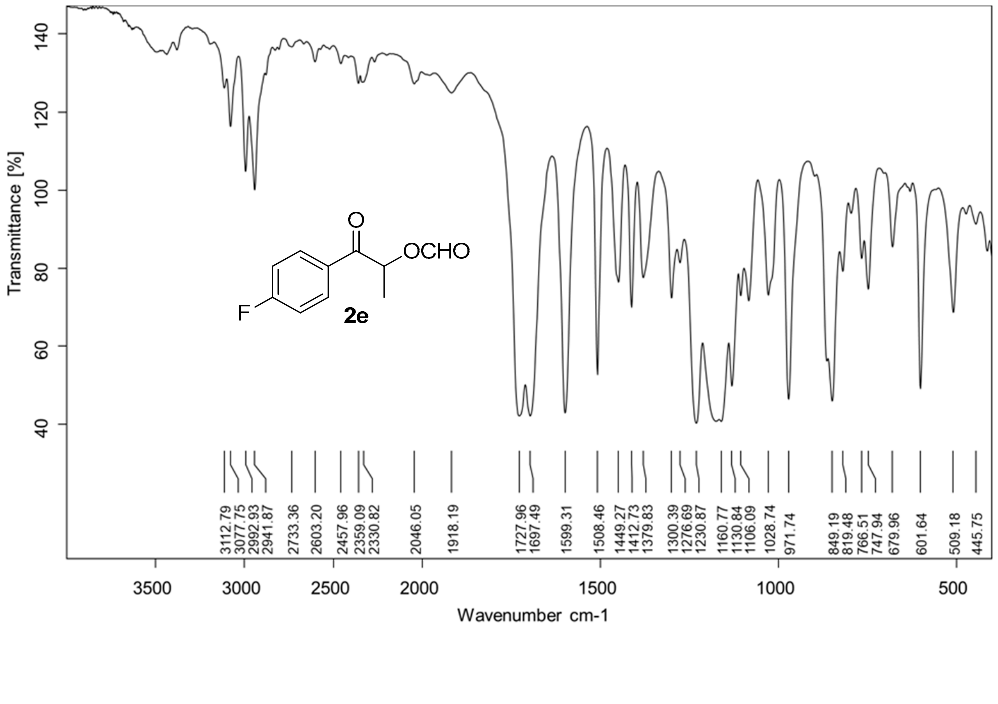

^1H NMR (500 MHz, $CDCl_3$) of 2f

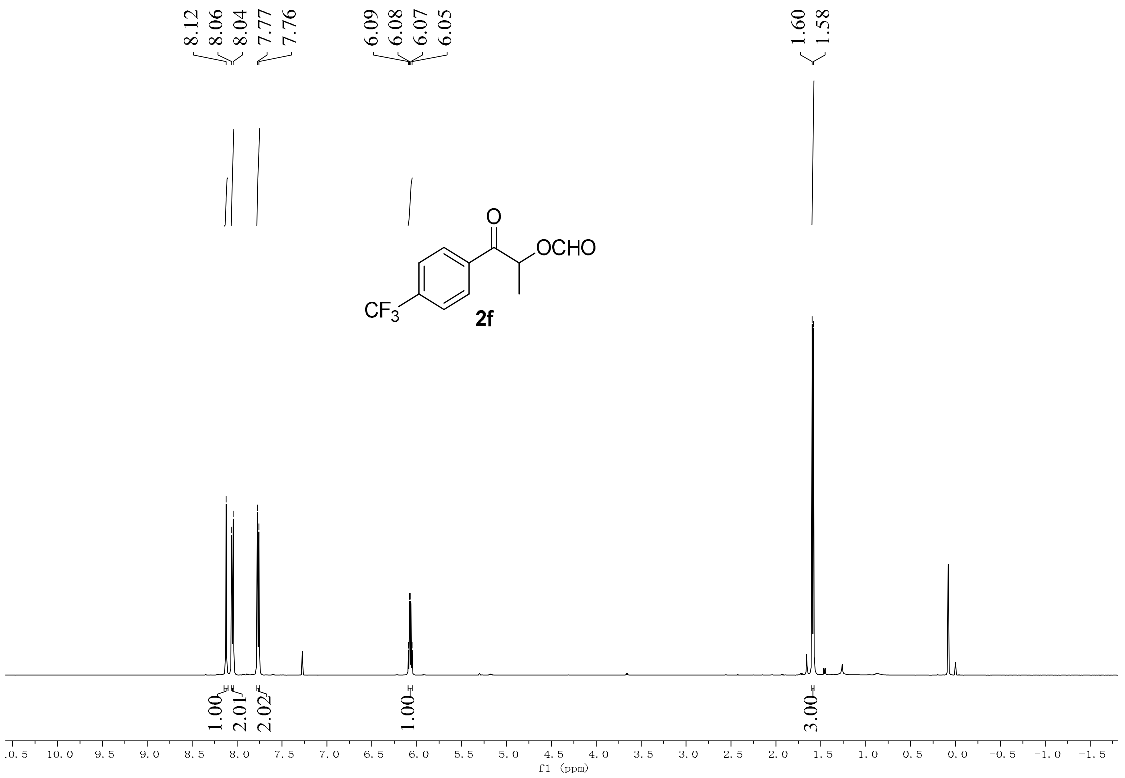

^{13}C NMR (126 MHz, $CDCl_3$) of 2f

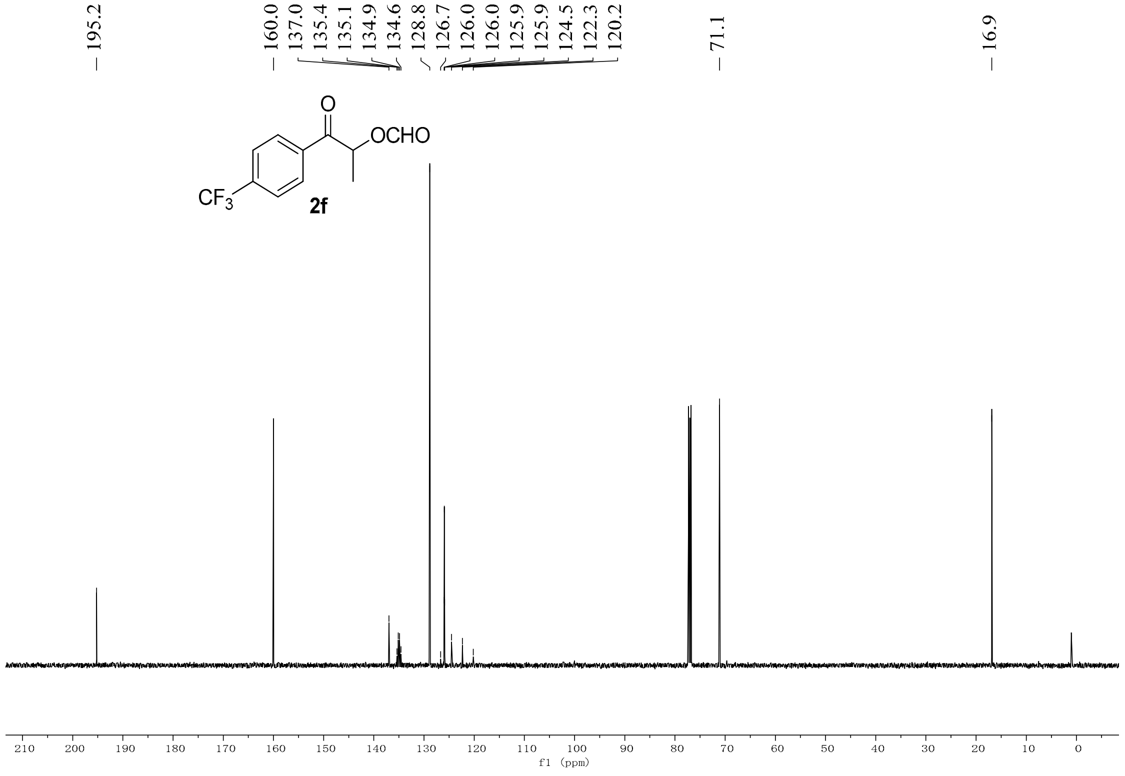

IR(KBr) of 2f

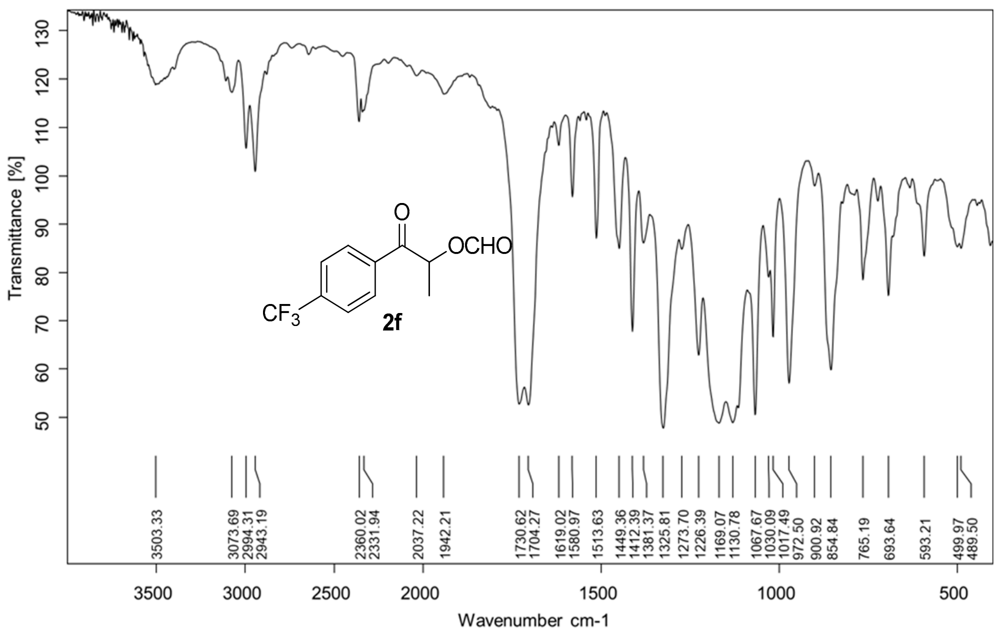

^1H NMR (400 MHz, $CDCl_3$) of 2g

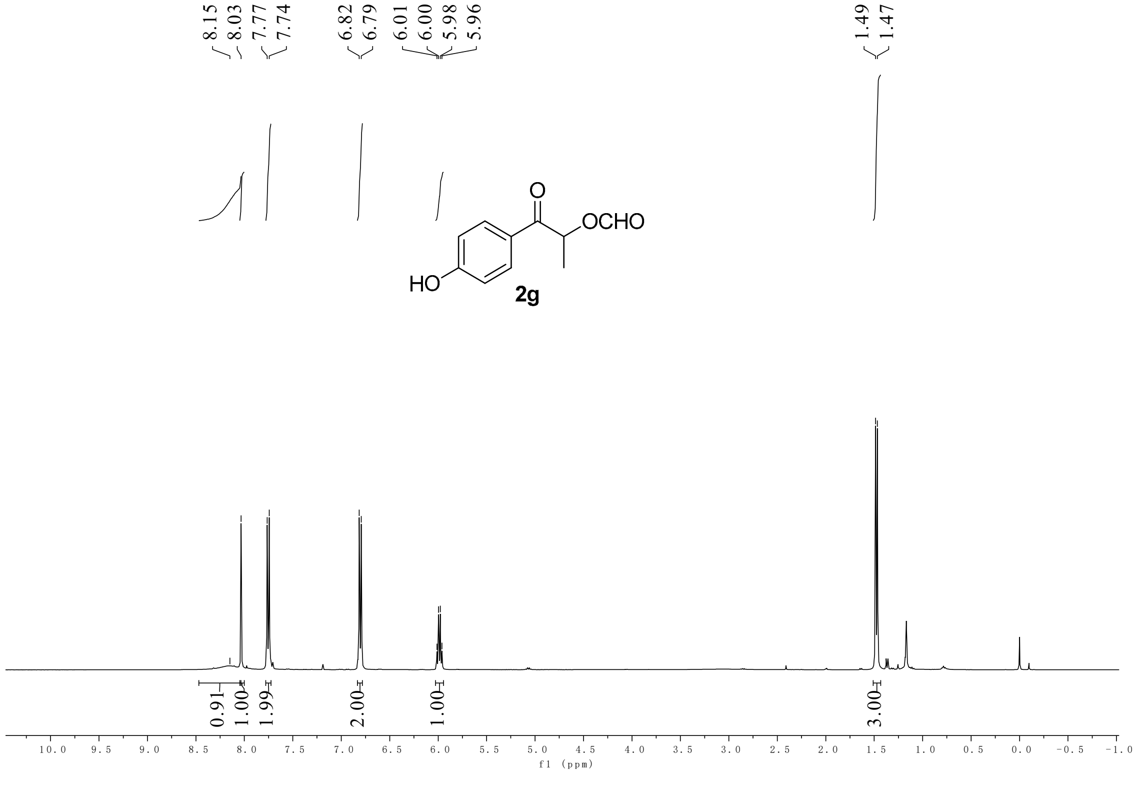

^{13}C NMR (101 MHz, $CDCl_3$) of 2g

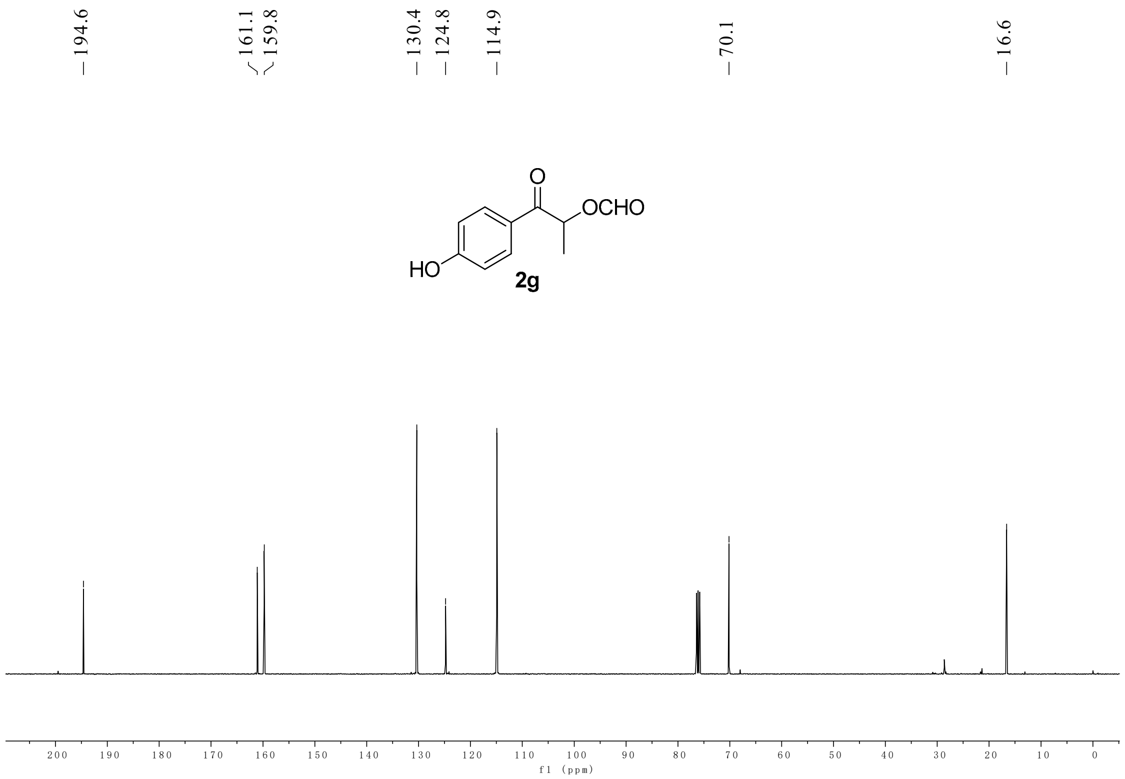

IR(KBr) of 2g

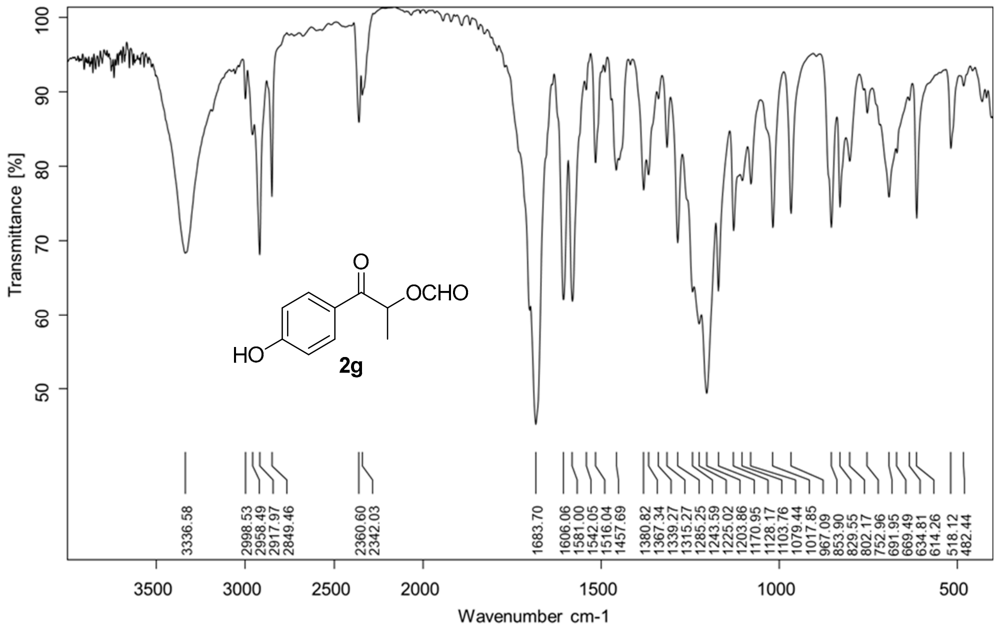

^{1}H NMR (500 MHz, $CDCl_3$) of 2h

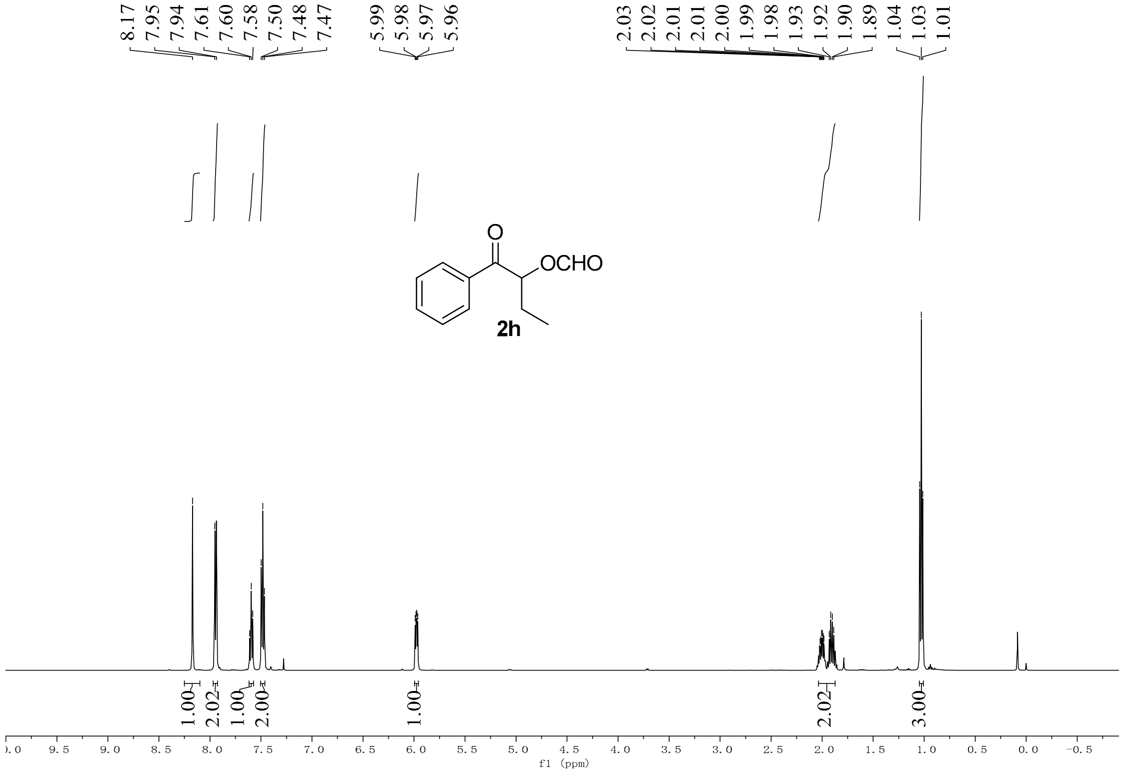

^{13}C NMR (126 MHz, $CDCl_3$) of 2h

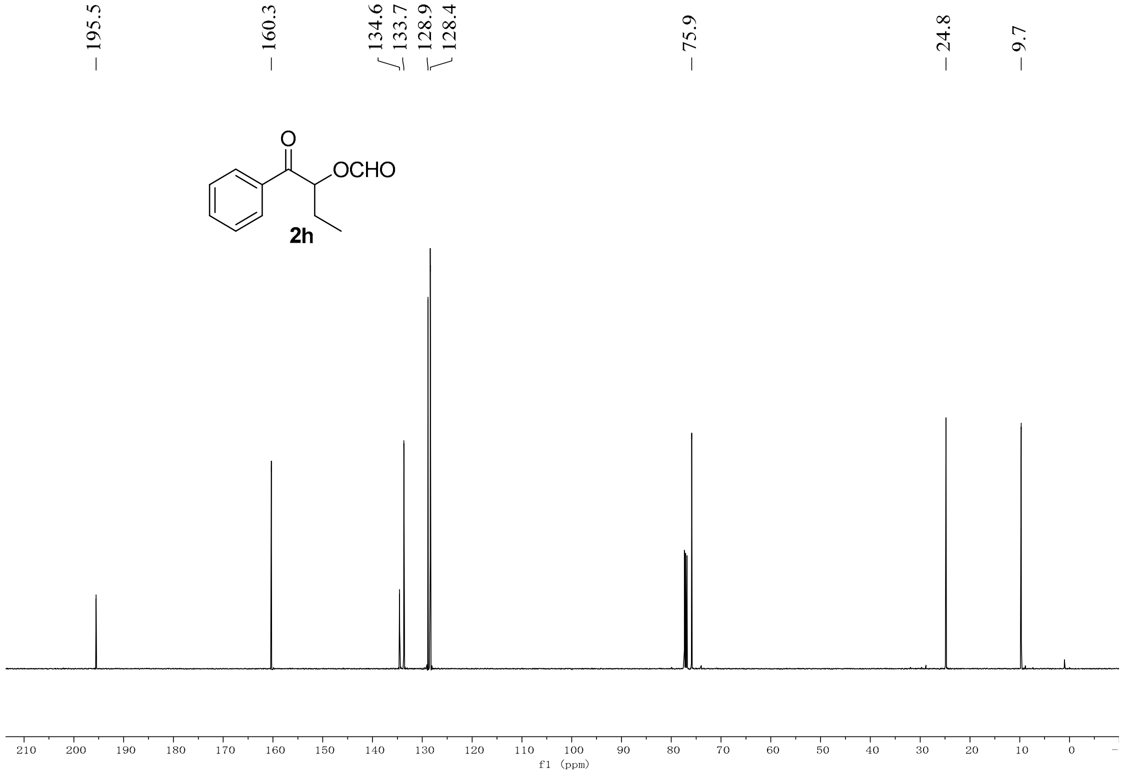

IR(KBr) of 2h

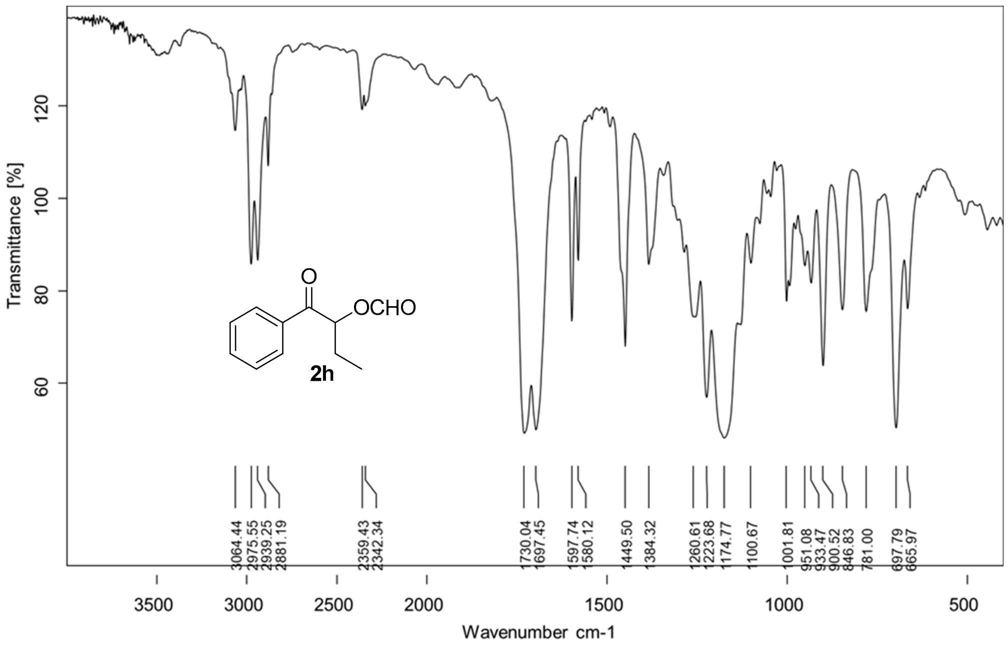

^{1}H NMR (500 MHz, $CDCl_3$) of 2i

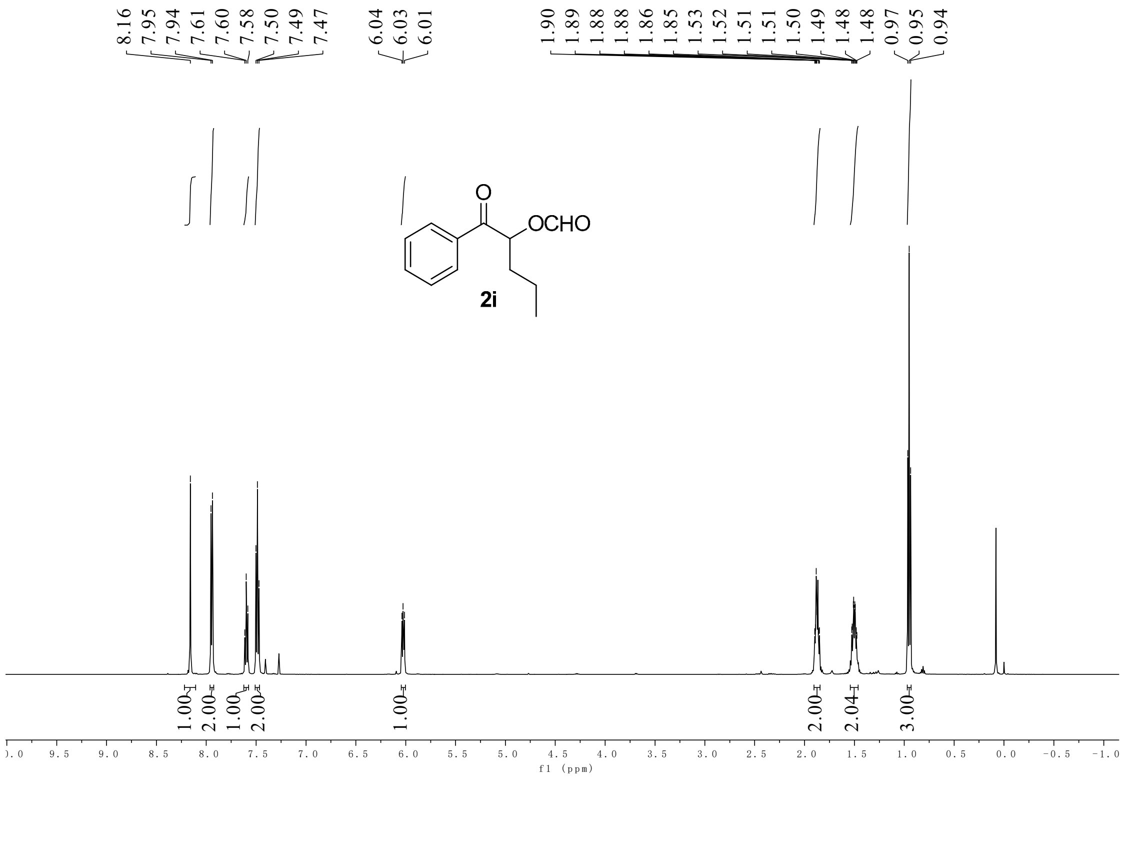

^{13}C NMR (126 MHz, $CDCl_3$) of 2i

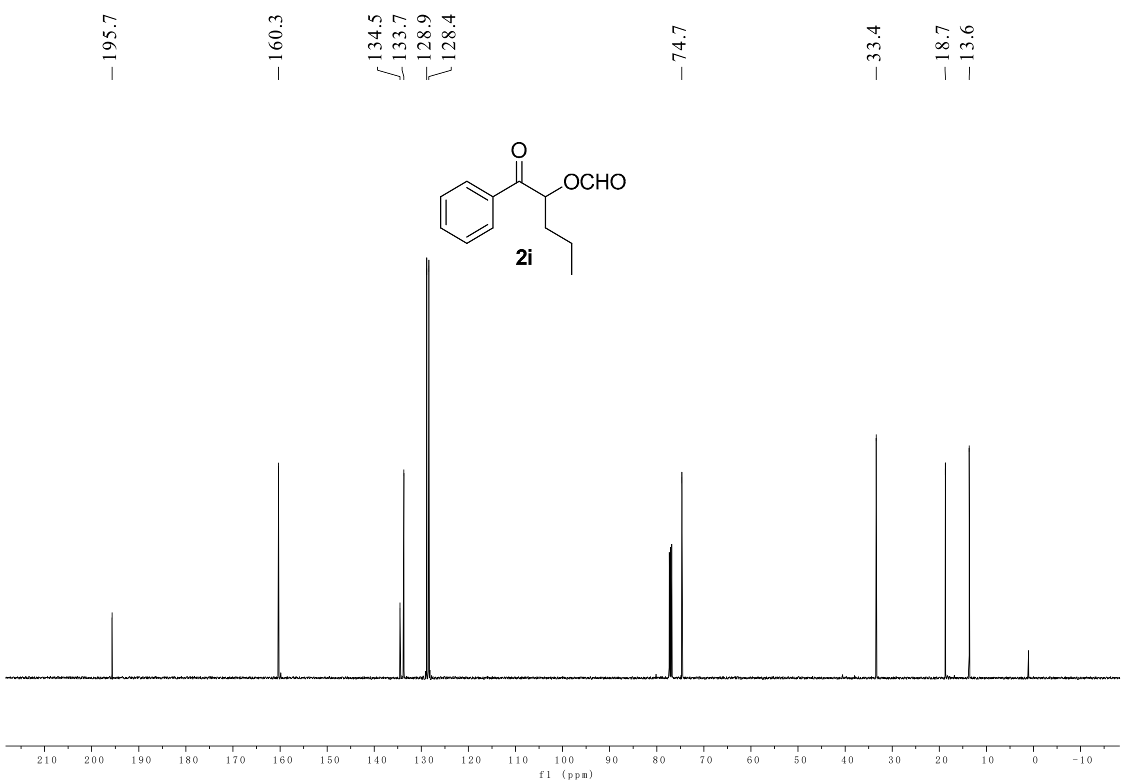

IR(KBr) of 2i

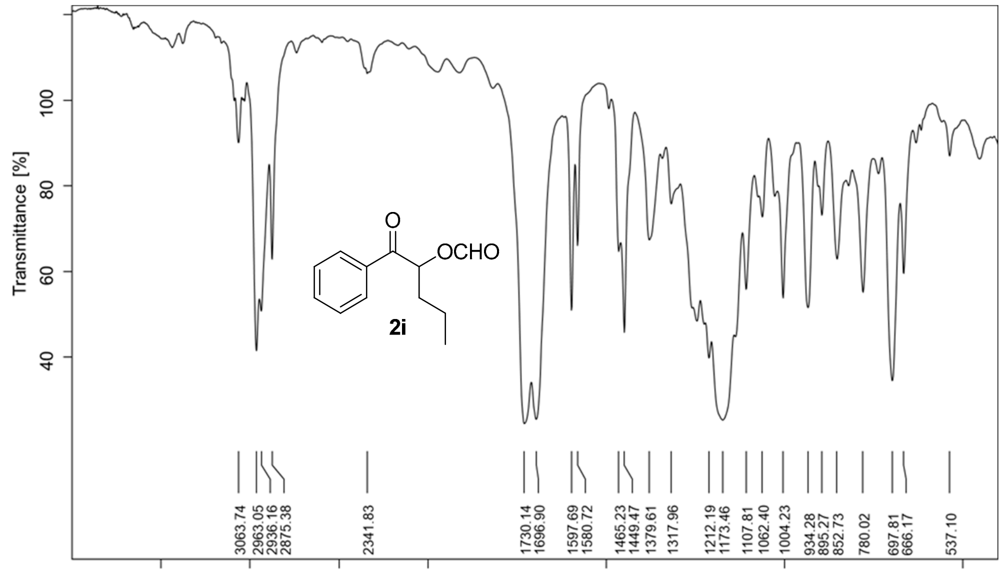

^{1}H NMR (500 MHz, $CDCl_3$) of 2j

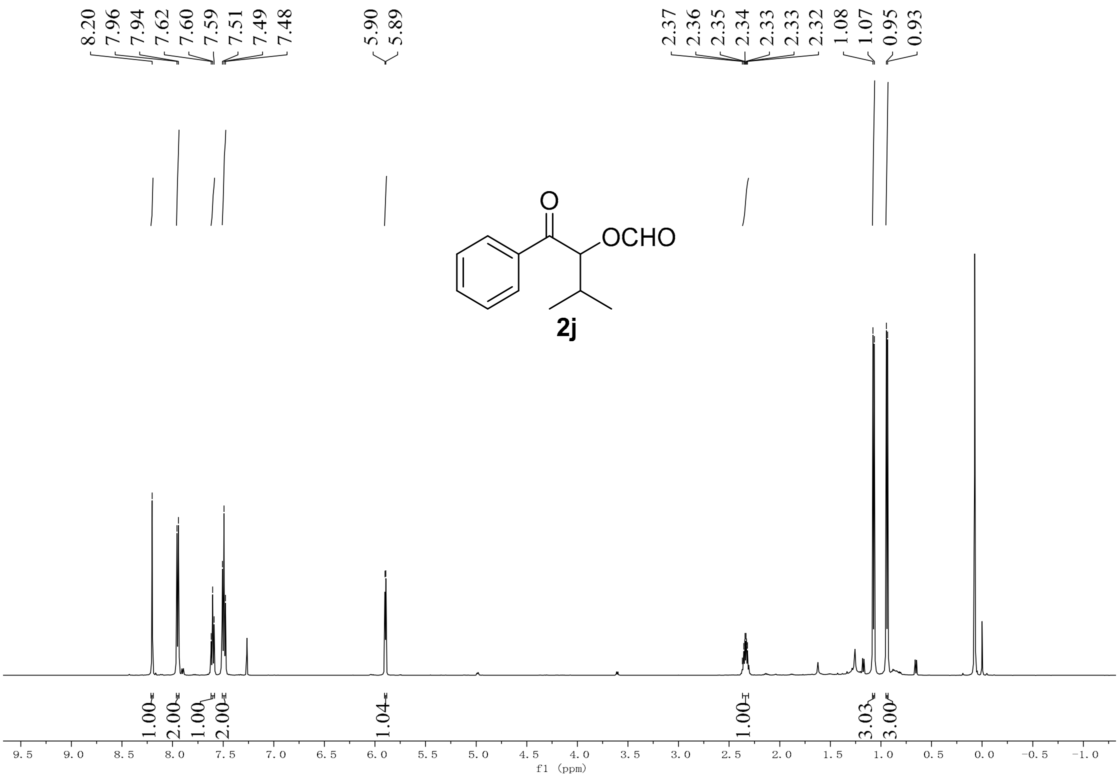

^{13}C NMR (126 MHz, $CDCl_3$) of 2j

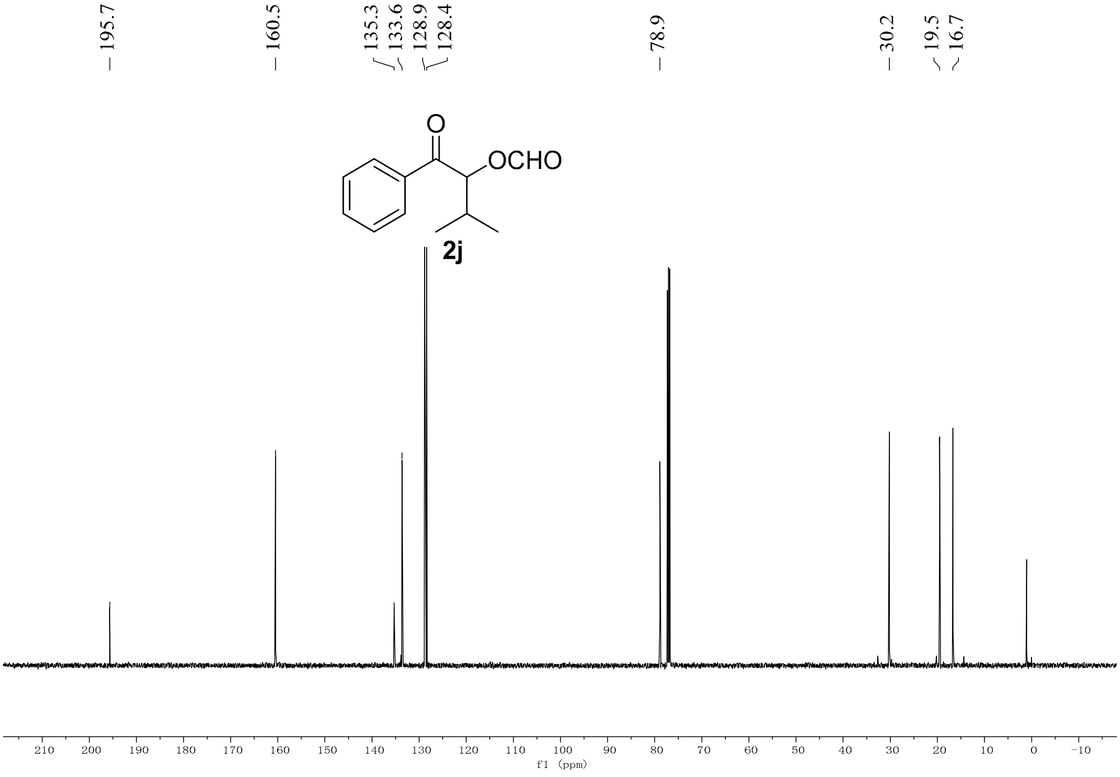

IR(KBr) of 2j

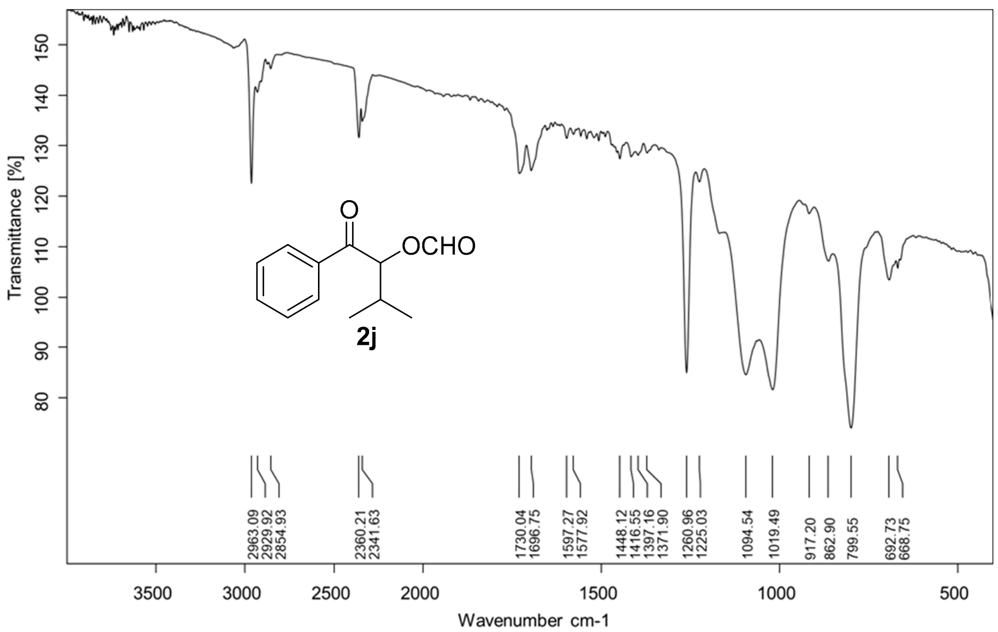

^{1}H NMR (500 MHz, $CDCl_3$) of 2k

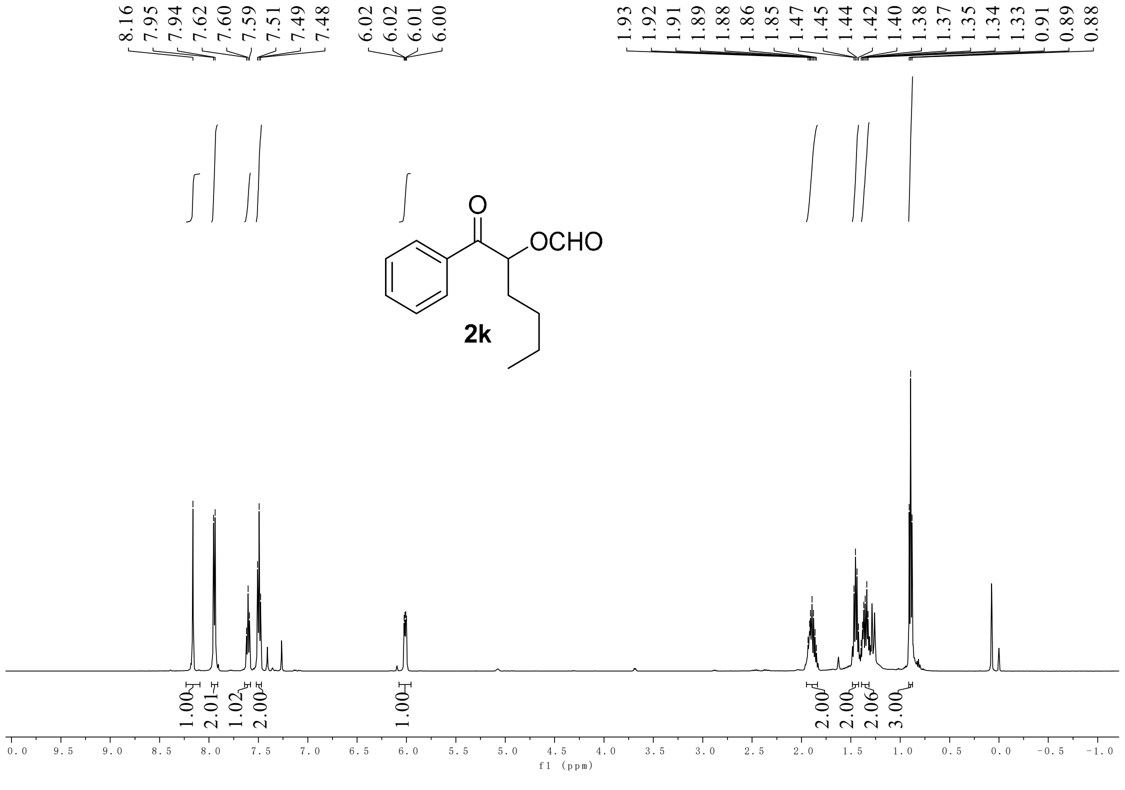

^{13}C NMR (126 MHz, $CDCl_3$) of 2k

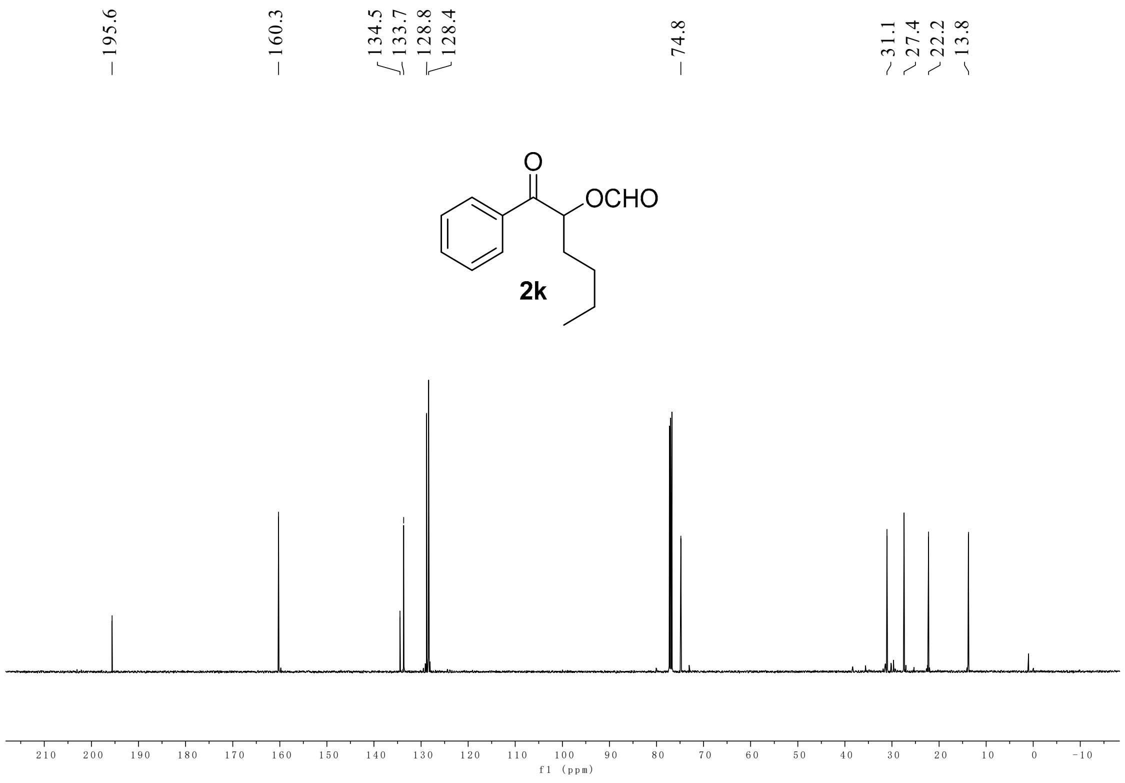

IR(KBr) of 2k

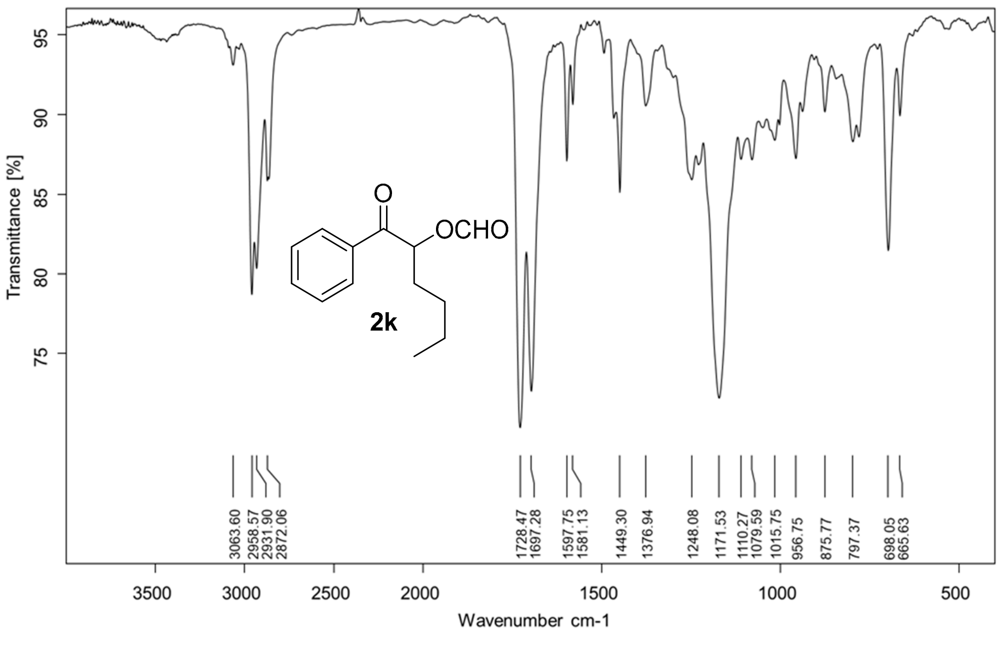

^{1}H NMR (500 MHz, $CDCl_3$) of 2l

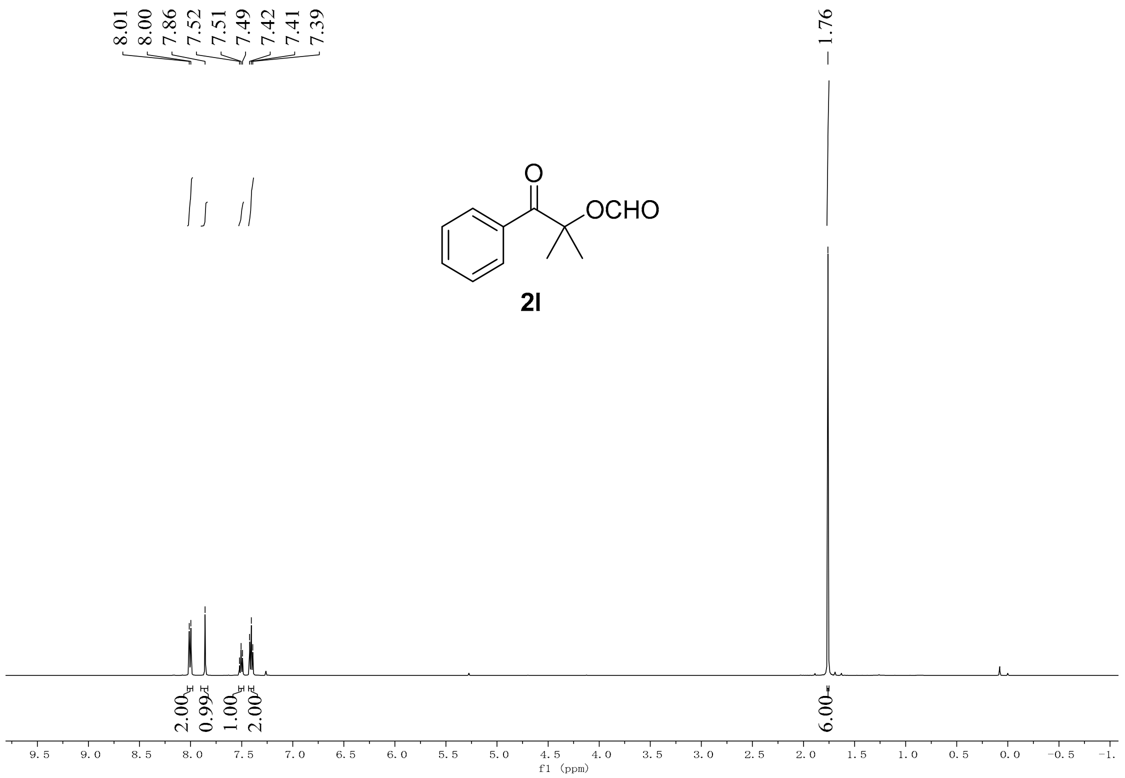

^{13}C NMR (126 MHz, $CDCl_3$) of 2l

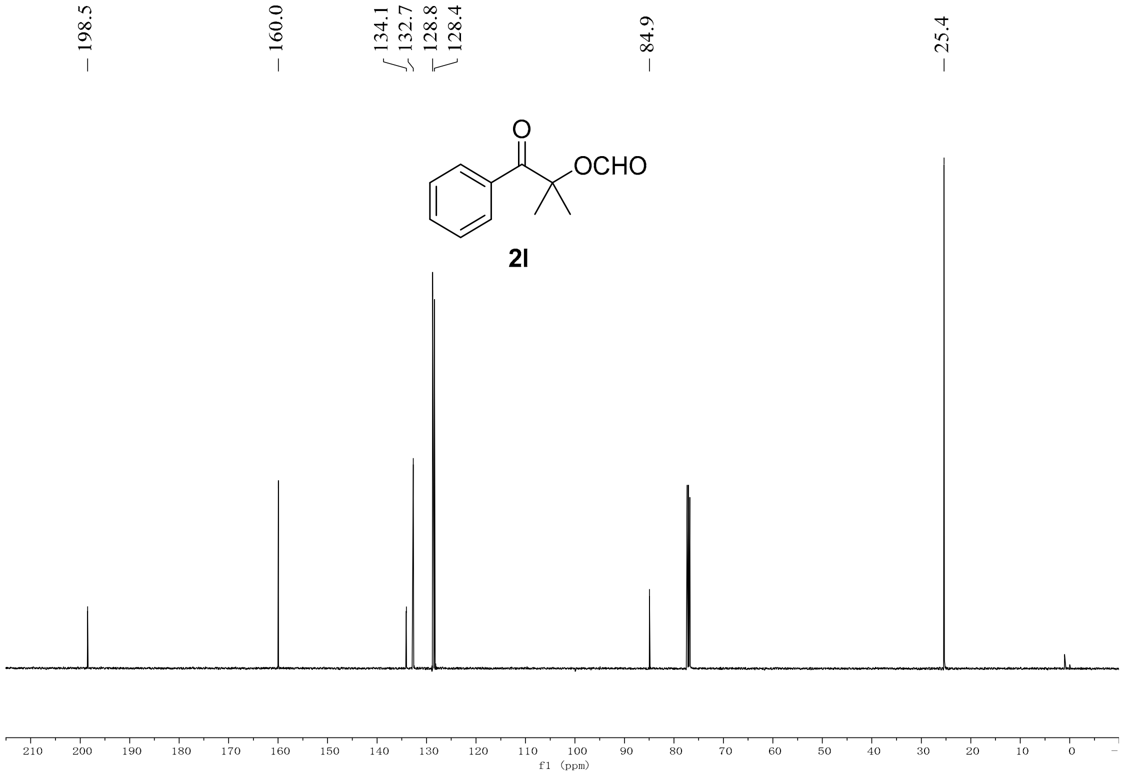

IR(KBr) of 2l

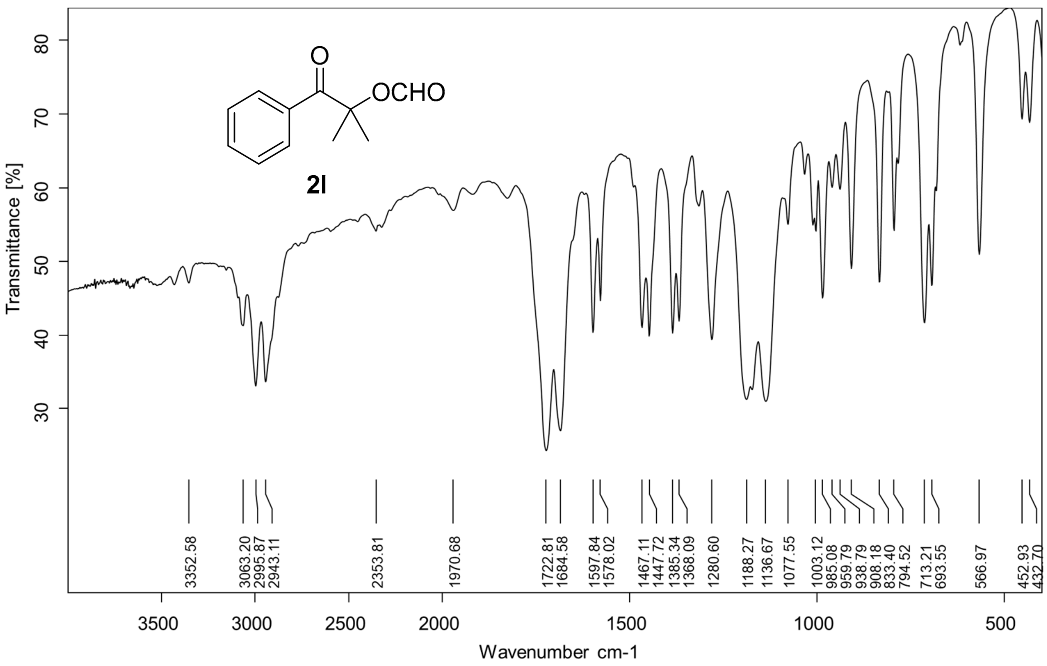

^{1}H NMR (500 MHz, $CDCl_3$) of 2m

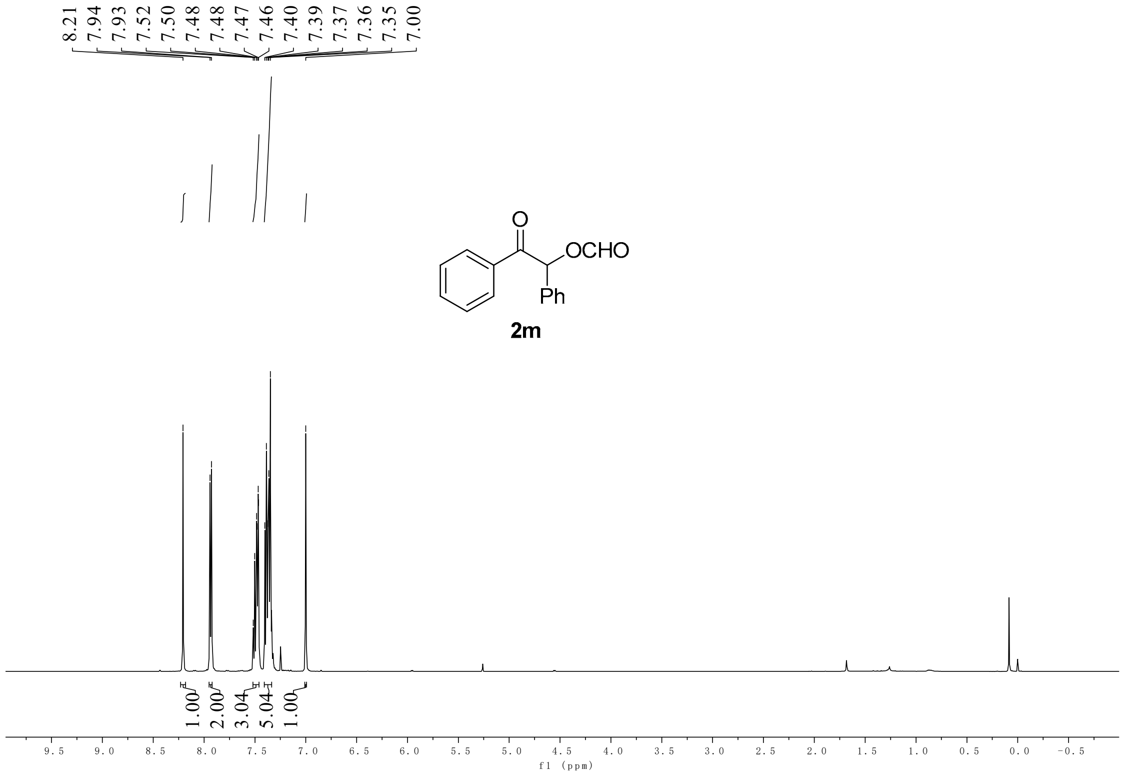

^{13}C NMR (126 MHz, $CDCl_3$) of 2m

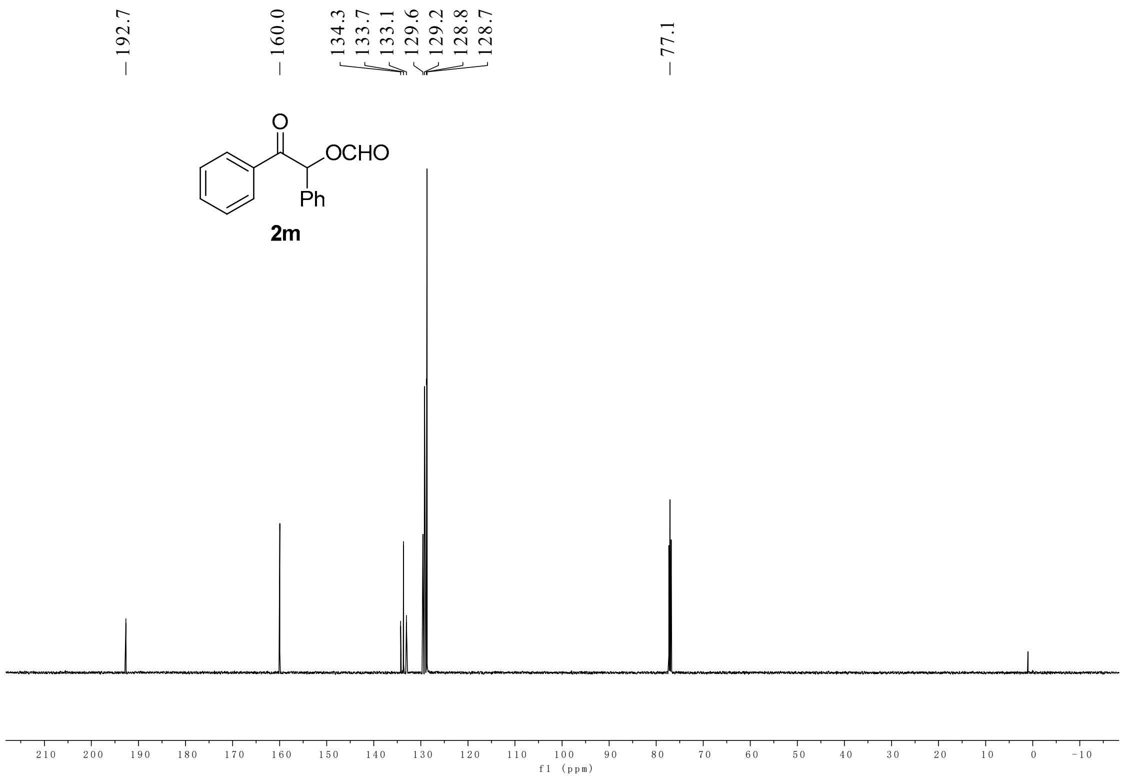

IR(KBr) of 2m

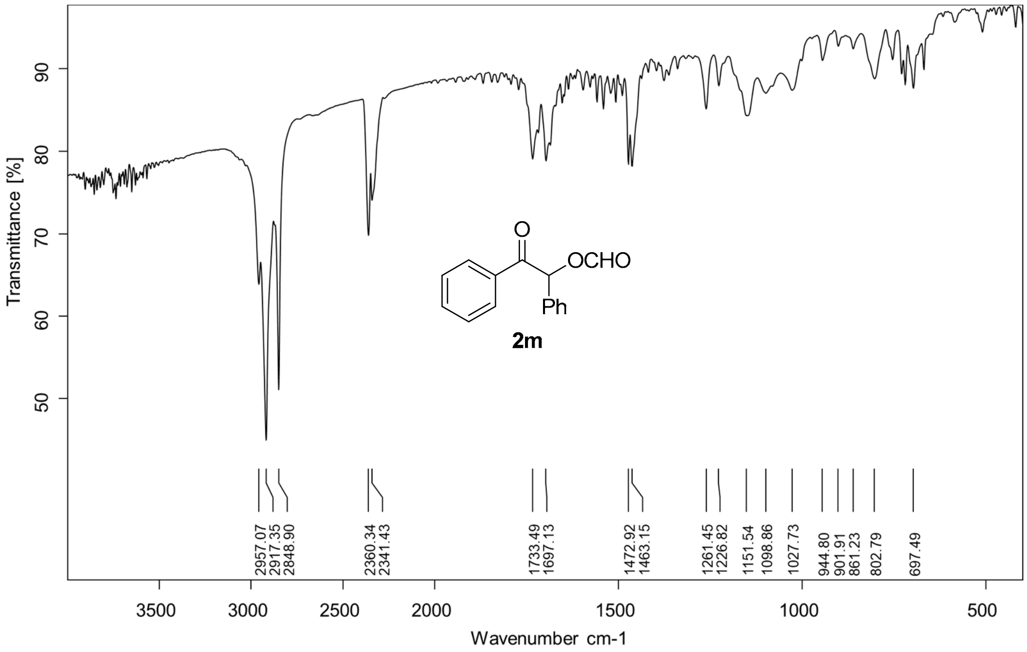

^1H NMR (400 MHz, $CDCl_3$) of 2n

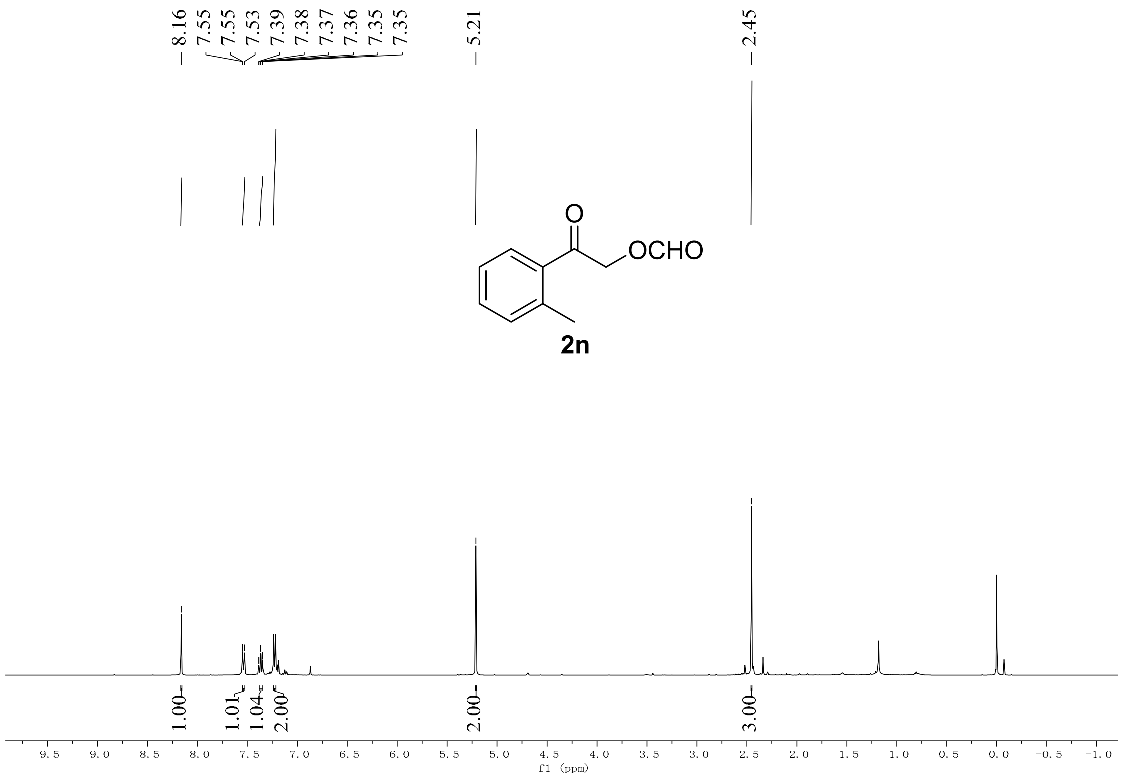

^{13}C NMR (101 MHz, $CDCl_3$) of 2n

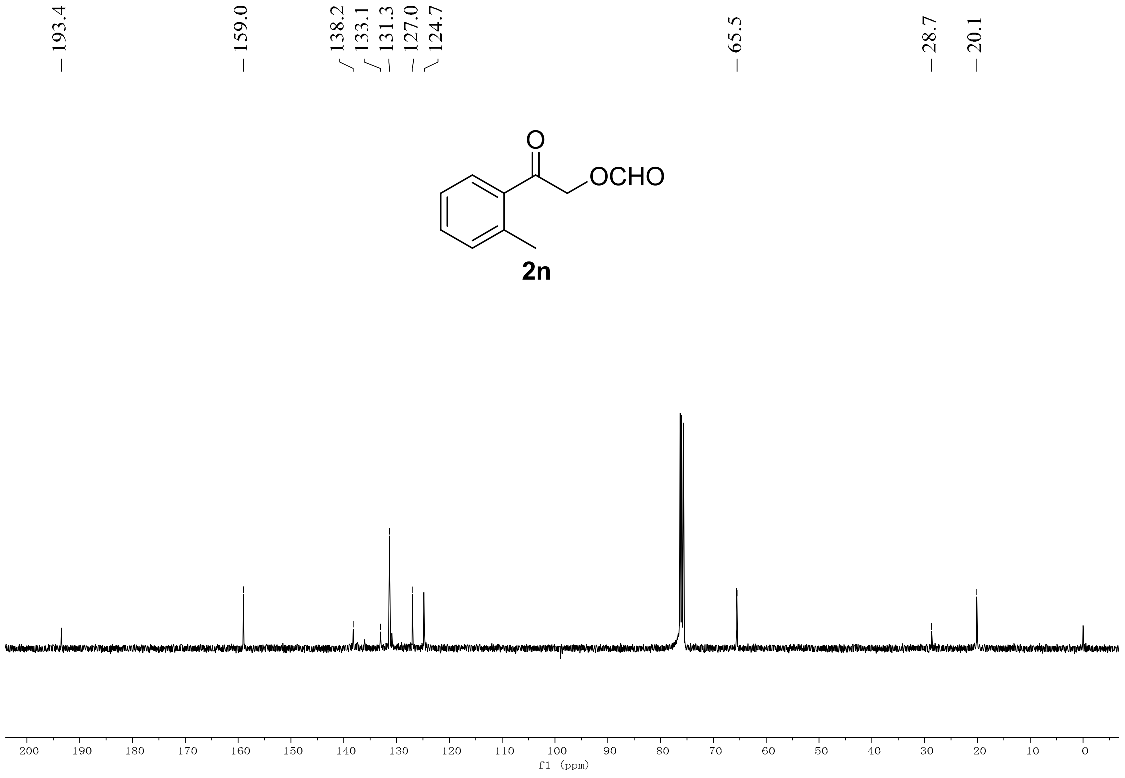

IR(KBr) of 2n

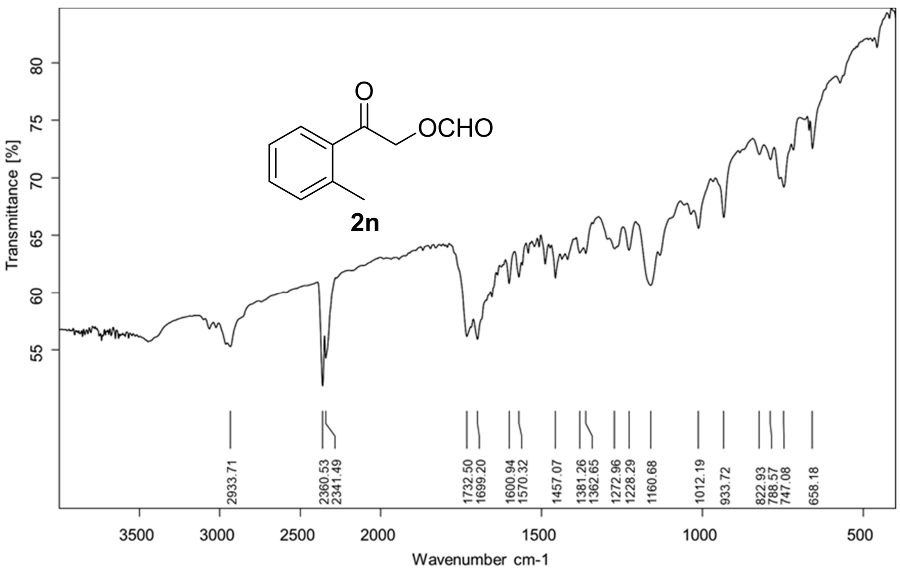

^{1}H NMR (400 MHz, $CDCl_3$) of 2o

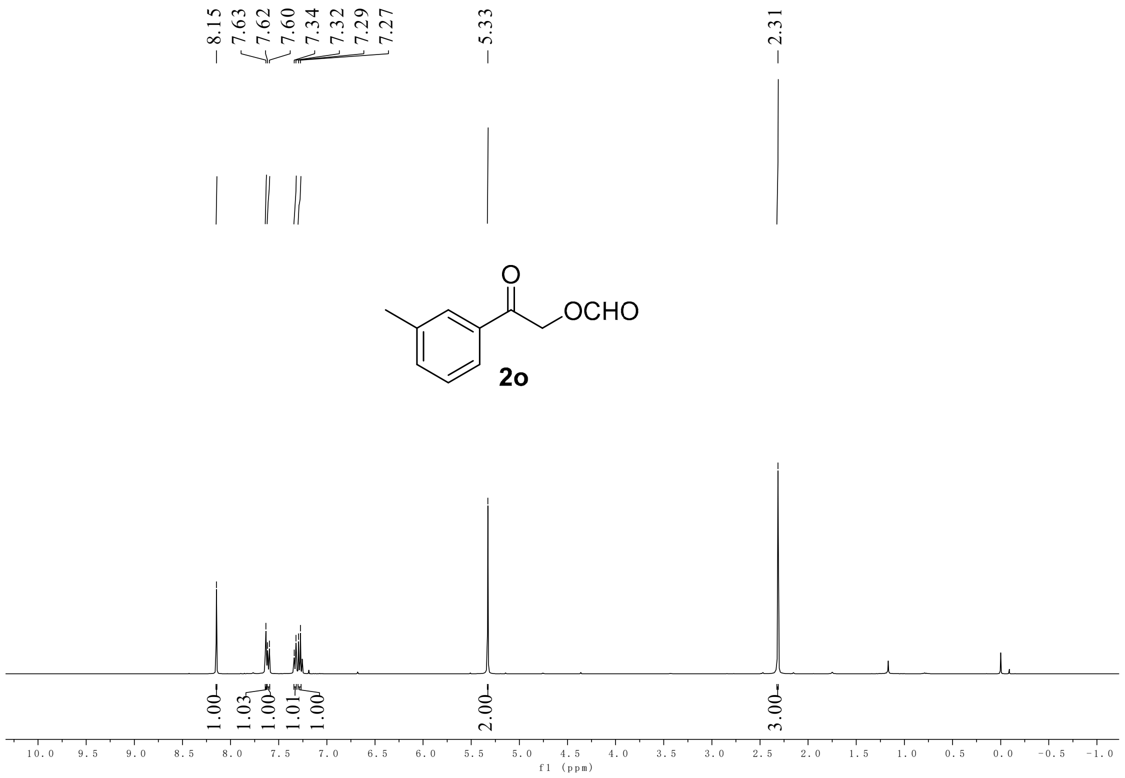

^{13}C NMR (101 MHz, $CDCl_3$) of 2o

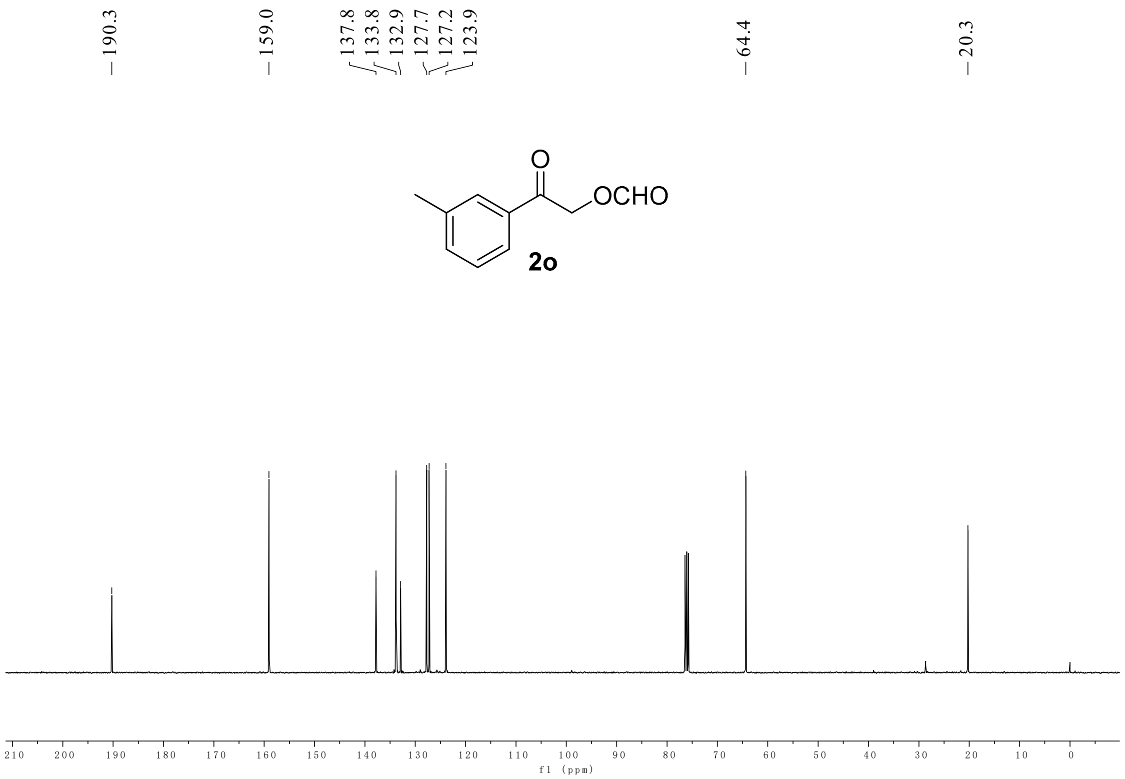

IR(KBr) of 2o

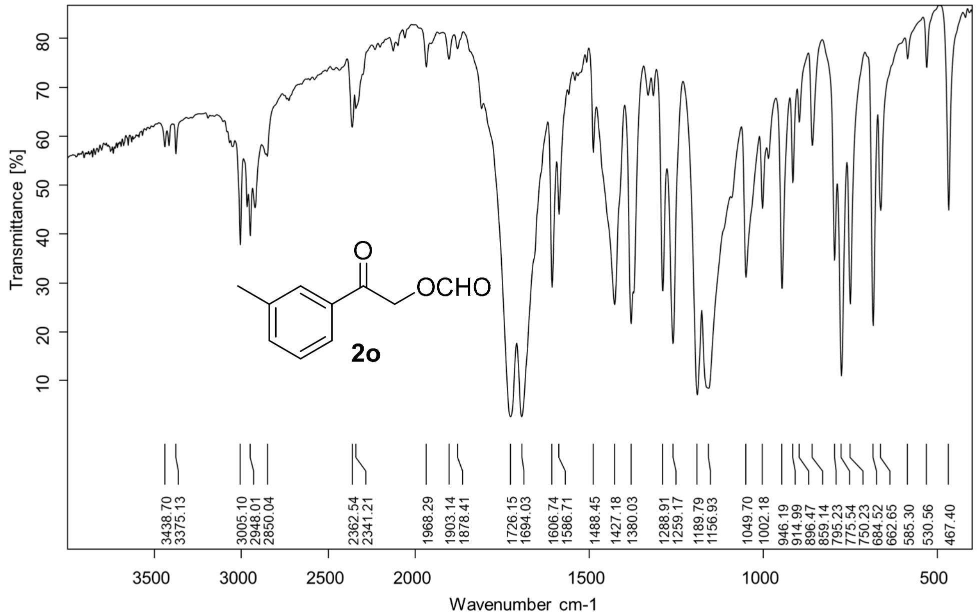

^{1}H NMR (400 MHz, $CDCl_3$) of 2p

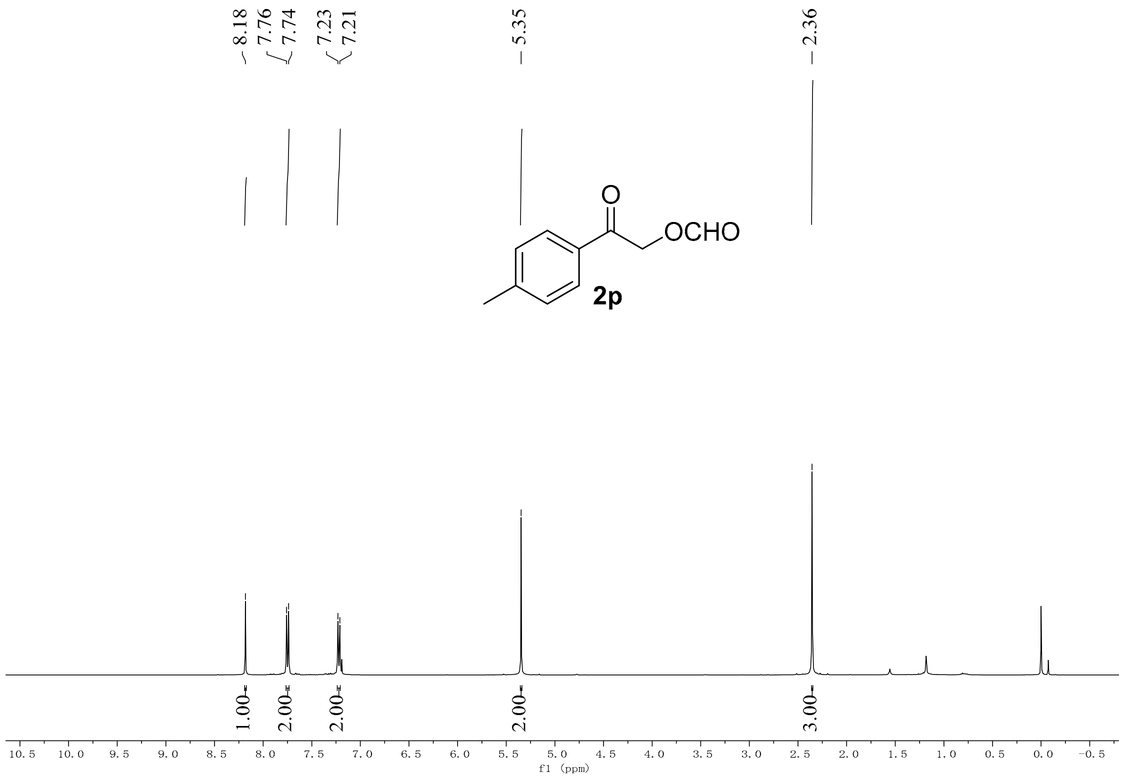

^{13}C NMR (101 MHz, $CDCl_3$) of 2p

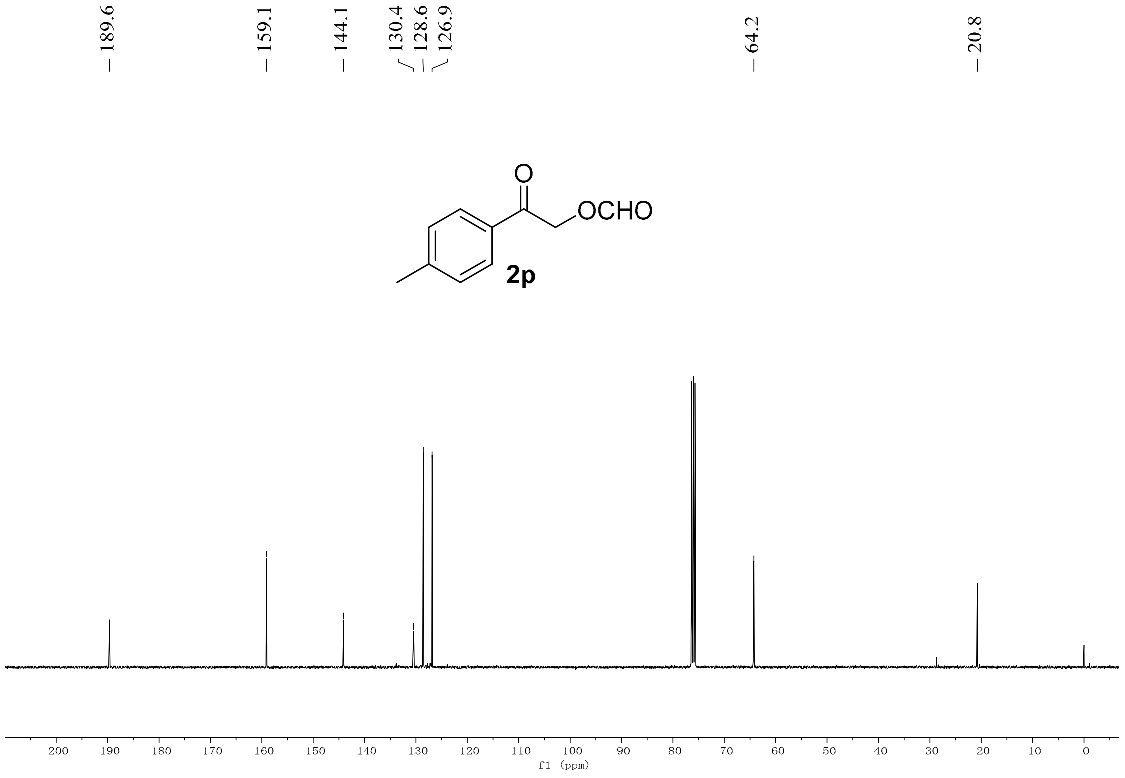

IR(KBr) of 2p

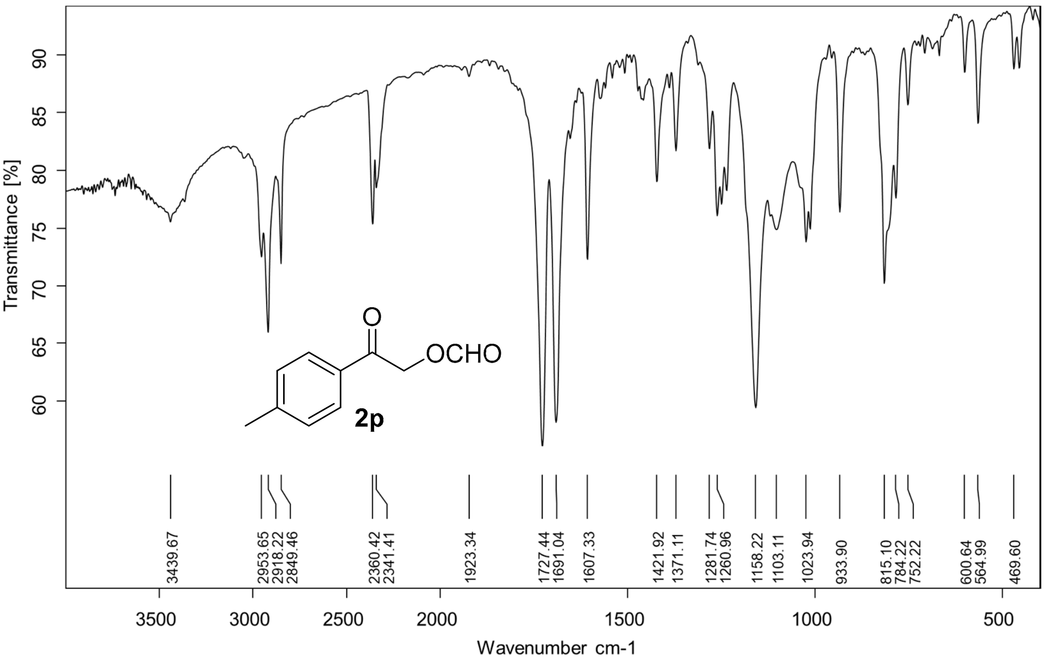

^{1}H NMR (500 MHz, $CDCl_3$) of 2q

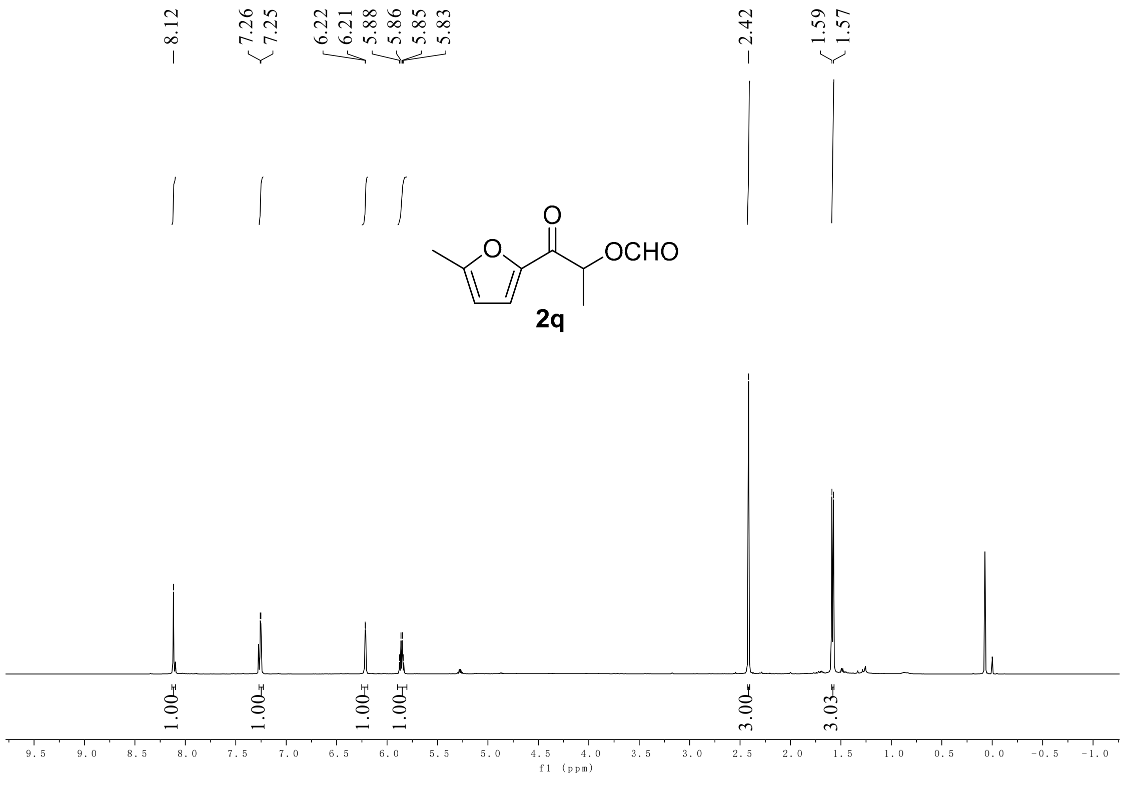

^{13}C NMR (126 MHz, $CDCl_3$) of 2q

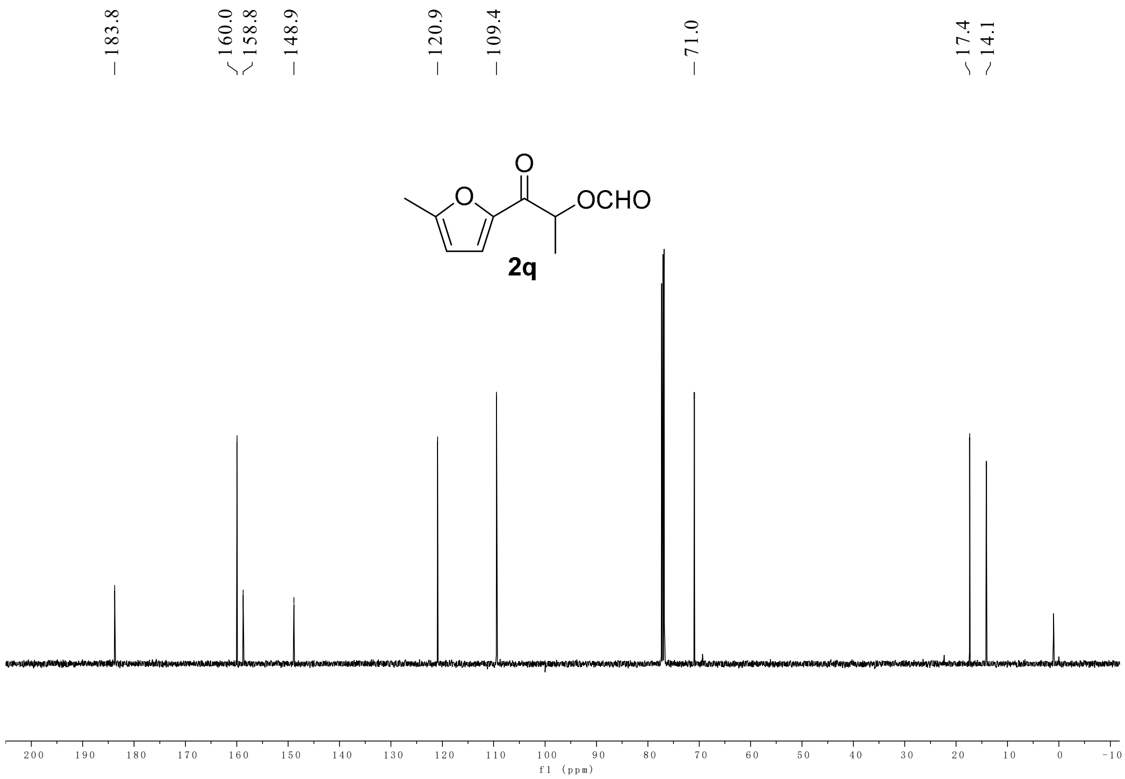

IR(KBr) of 2q

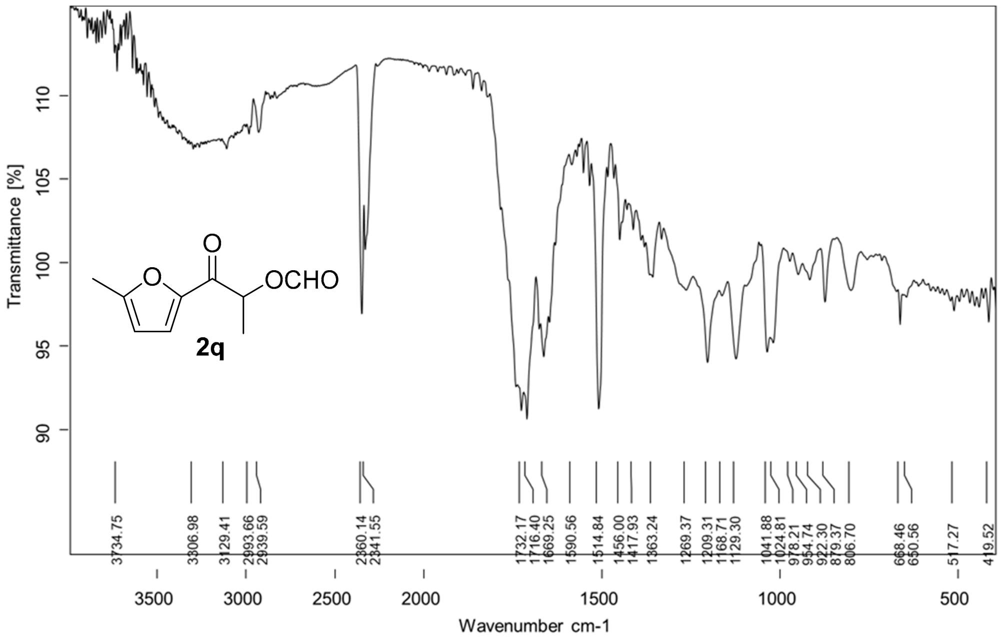

^{1}H NMR (500 MHz, $CDCl_3$) of 2r

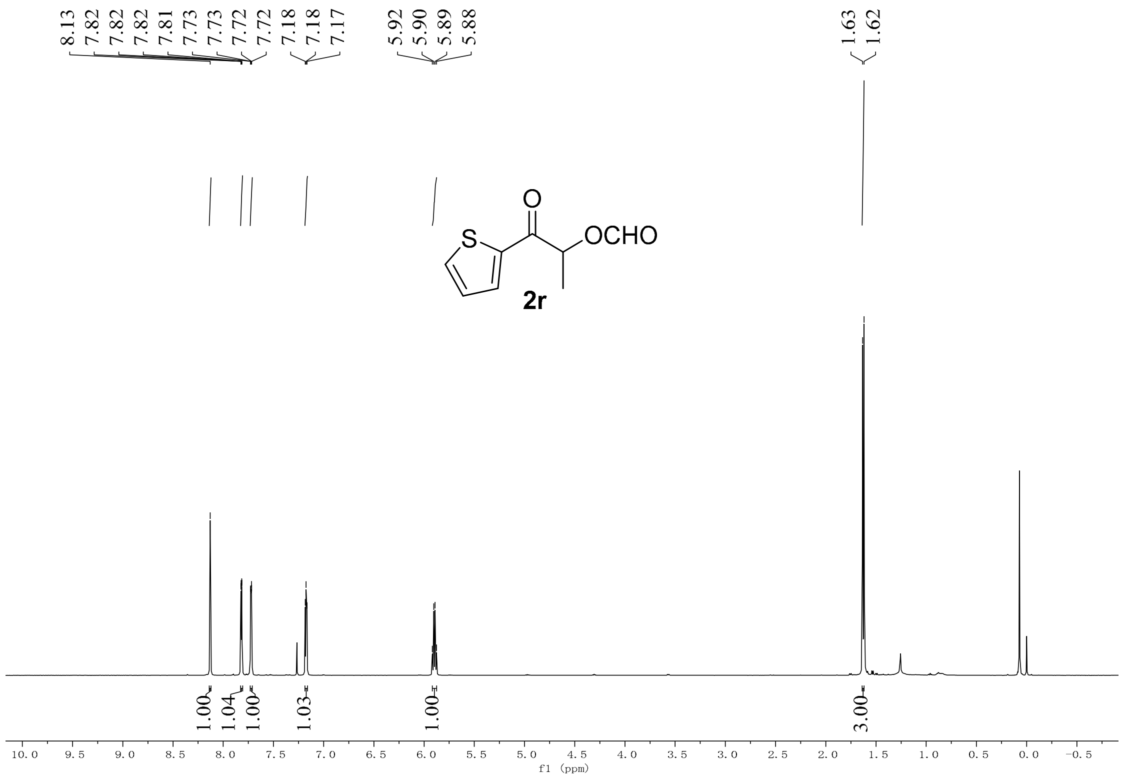

^{13}C NMR (126 MHz, $CDCl_3$) of 2r

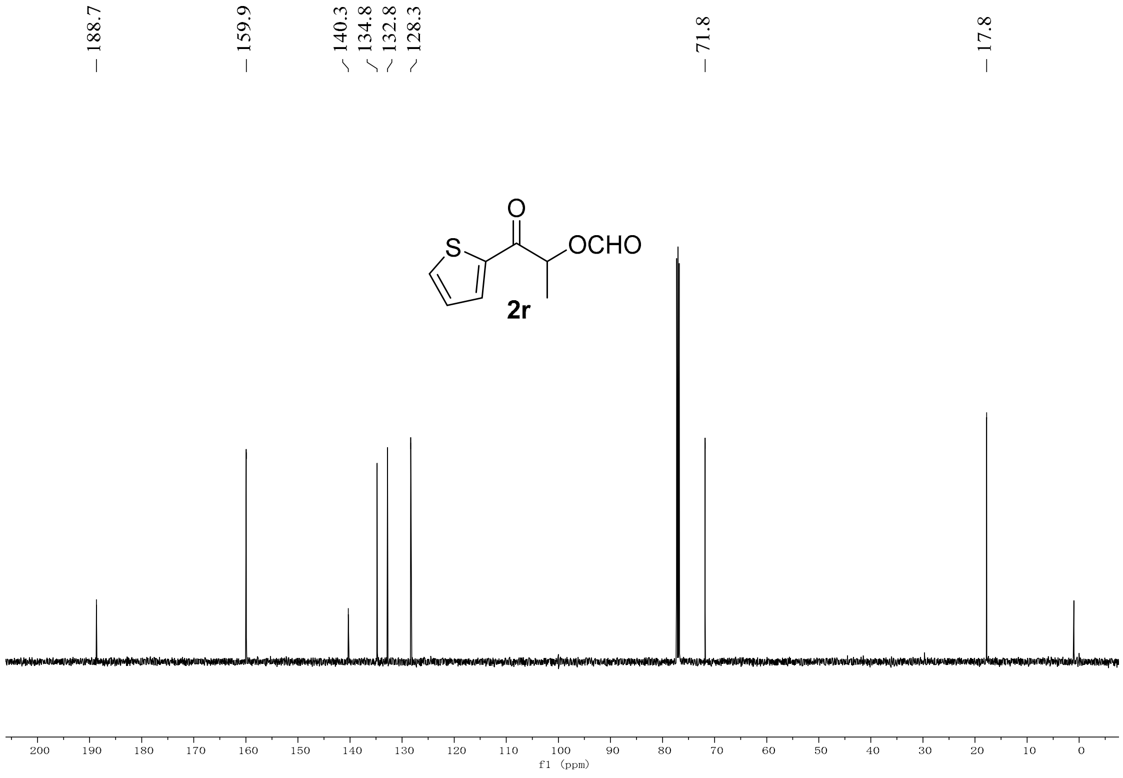

IR(KBr) of 2r

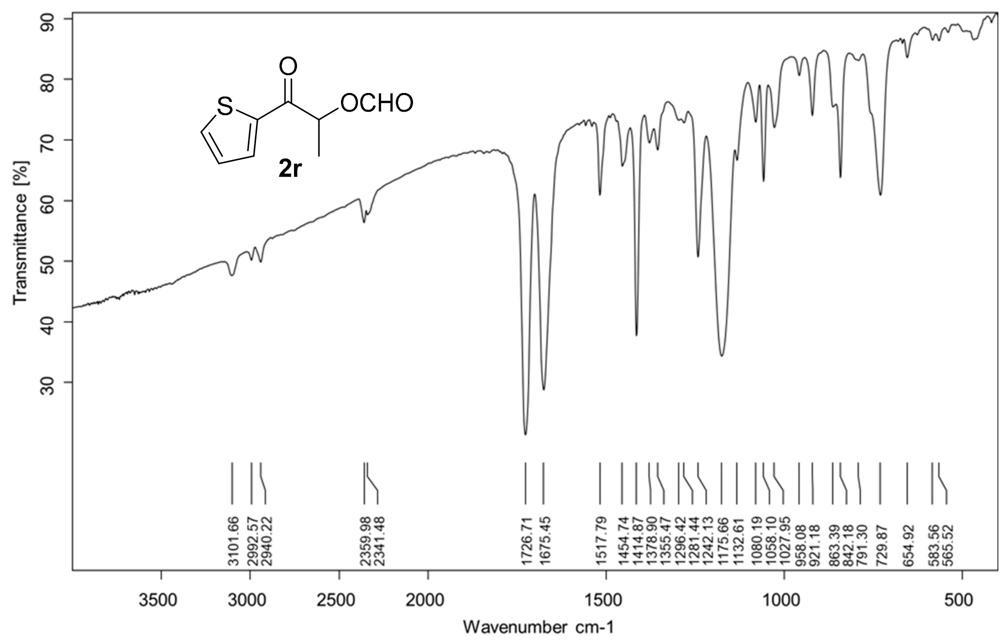

^{1}H NMR (400 MHz, $CDCl_3$) of 2s

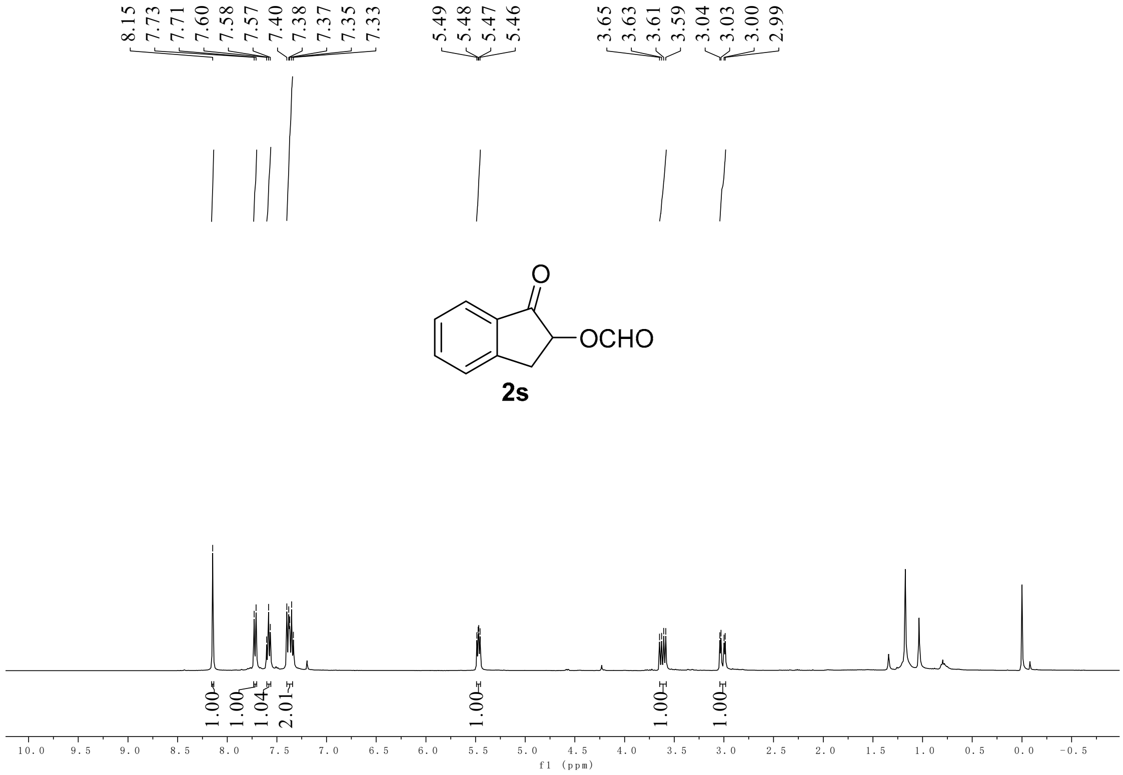

^{13}C NMR (101 MHz, $CDCl_3$) of 2s

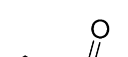

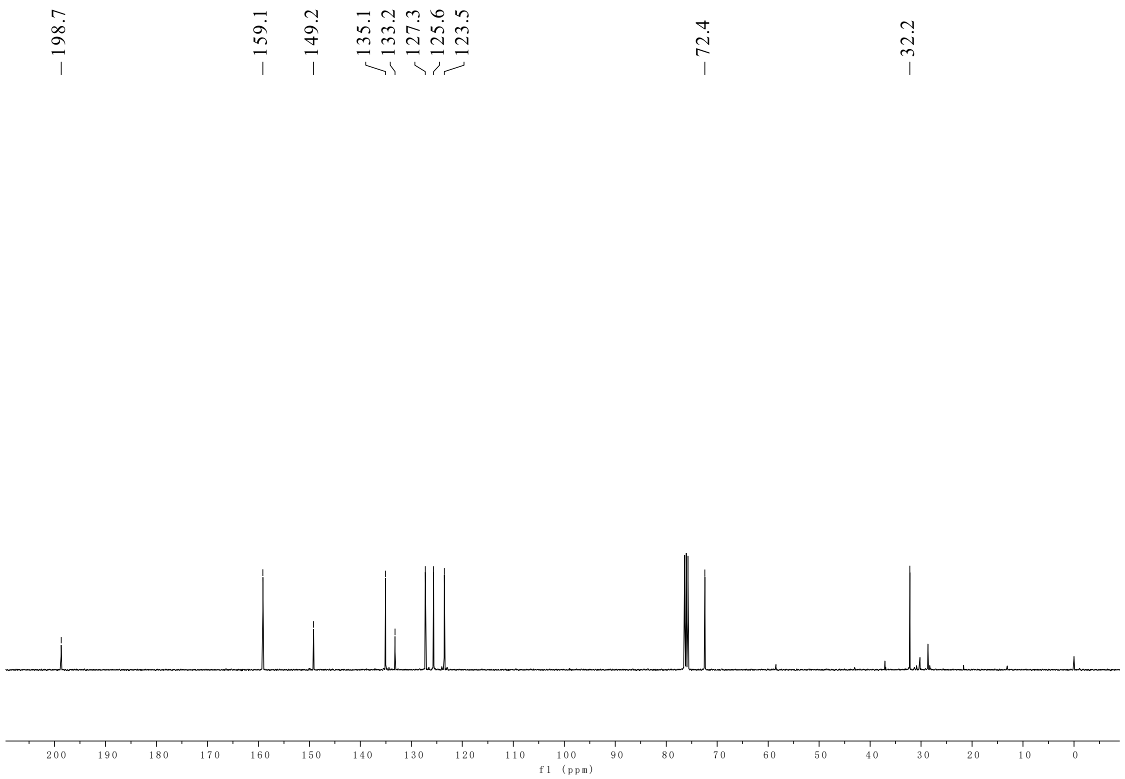

IR(KBr) of 2S

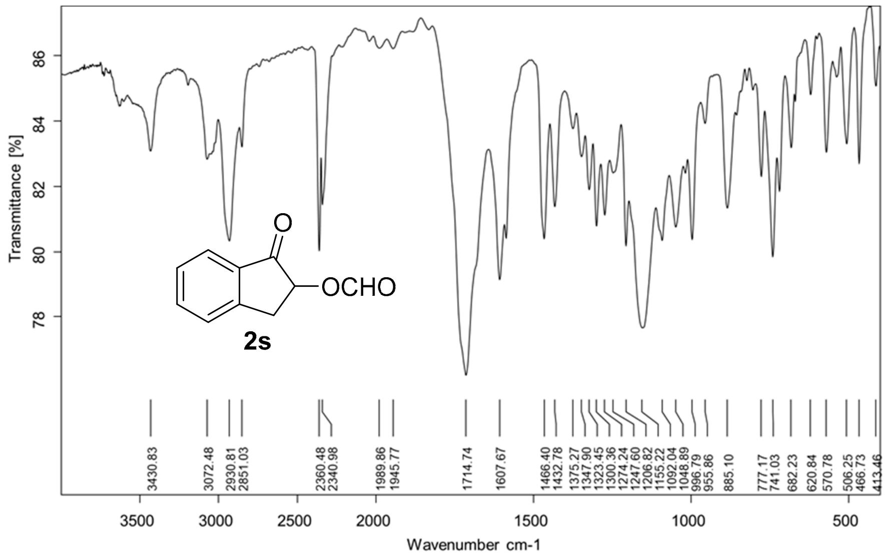

^{1}H NMR (400 MHz, $CDCl_3$) of 2t

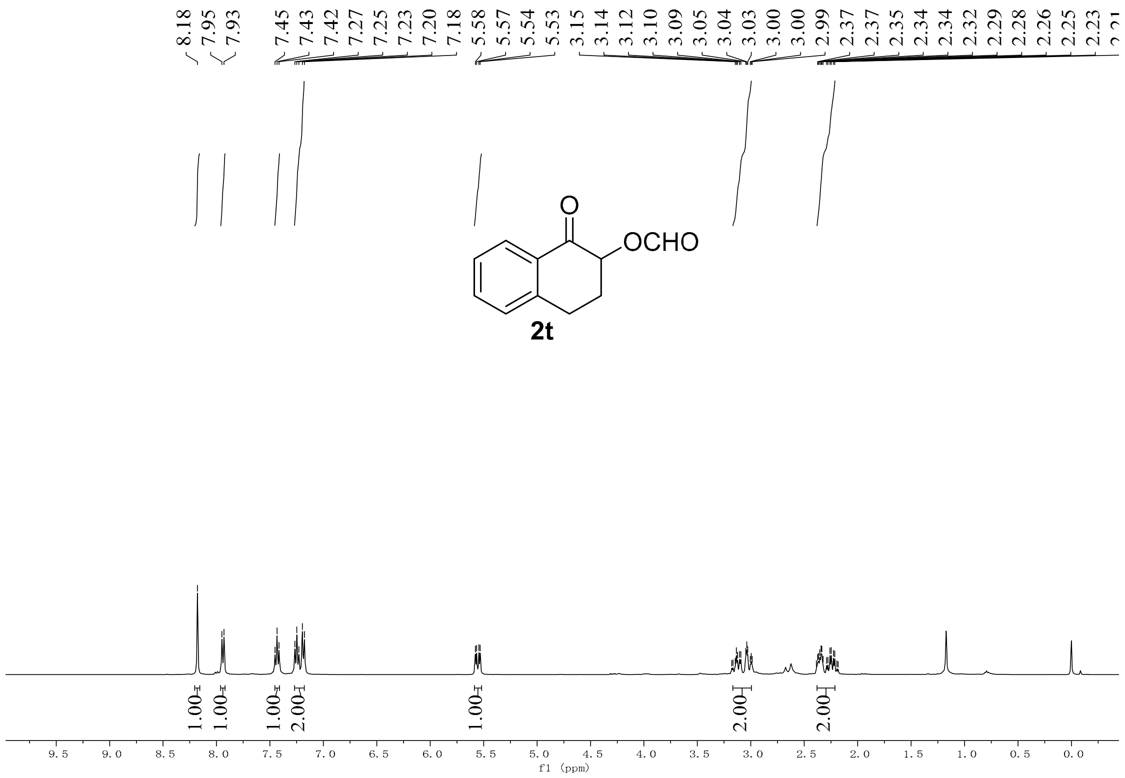

^{13}C NMR (101 MHz, $CDCl_3$) of 2t

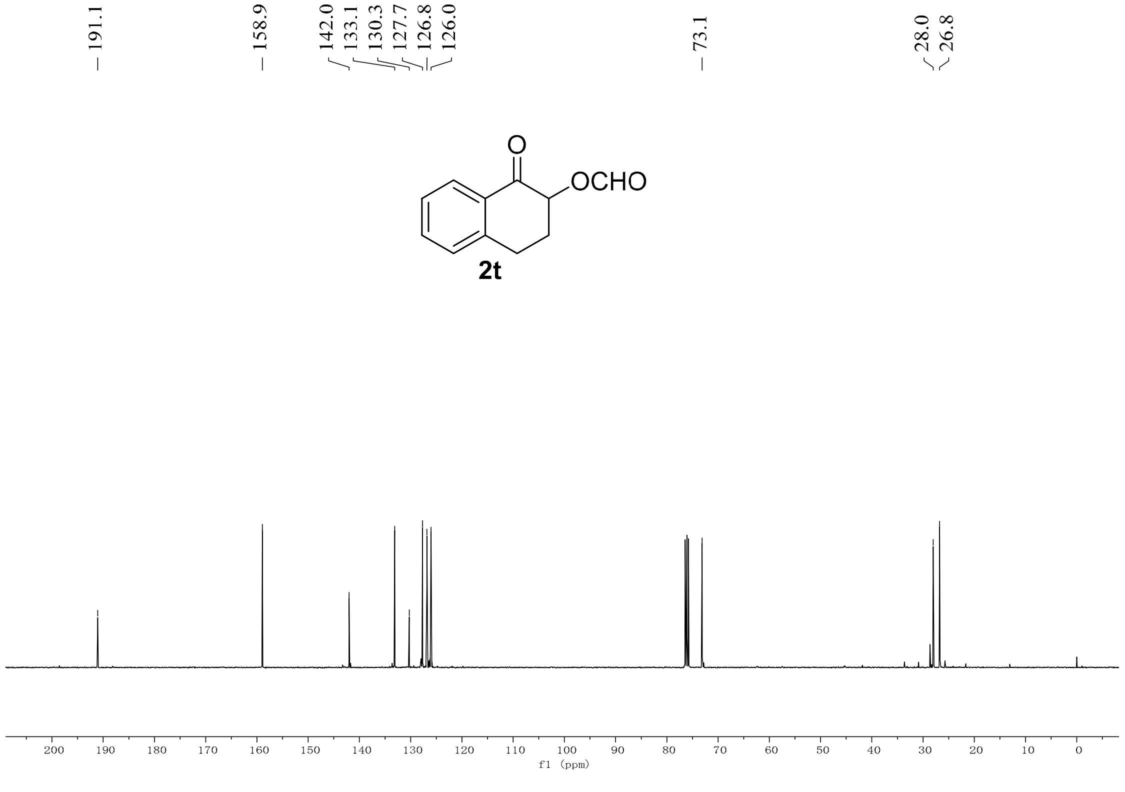

^{1}H NMR (500 MHz, $CDCl_3$) of 3a

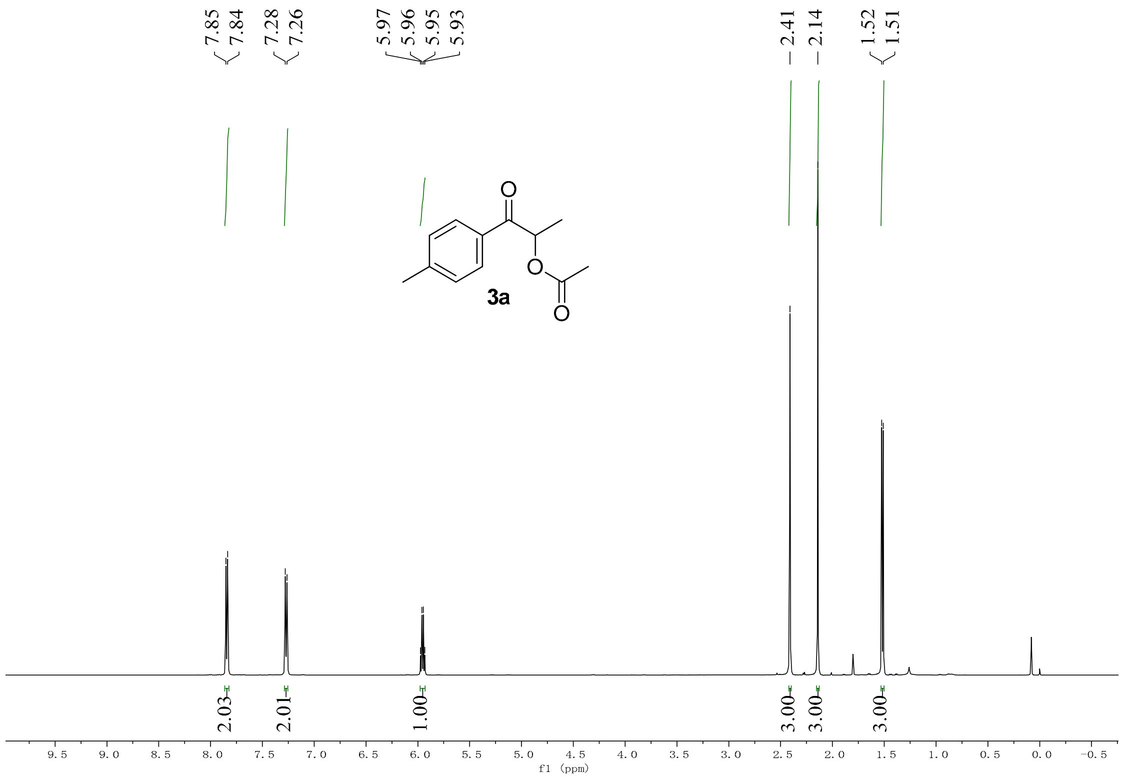

^{13}C NMR (126 MHz, $CDCl_3$) of 3a

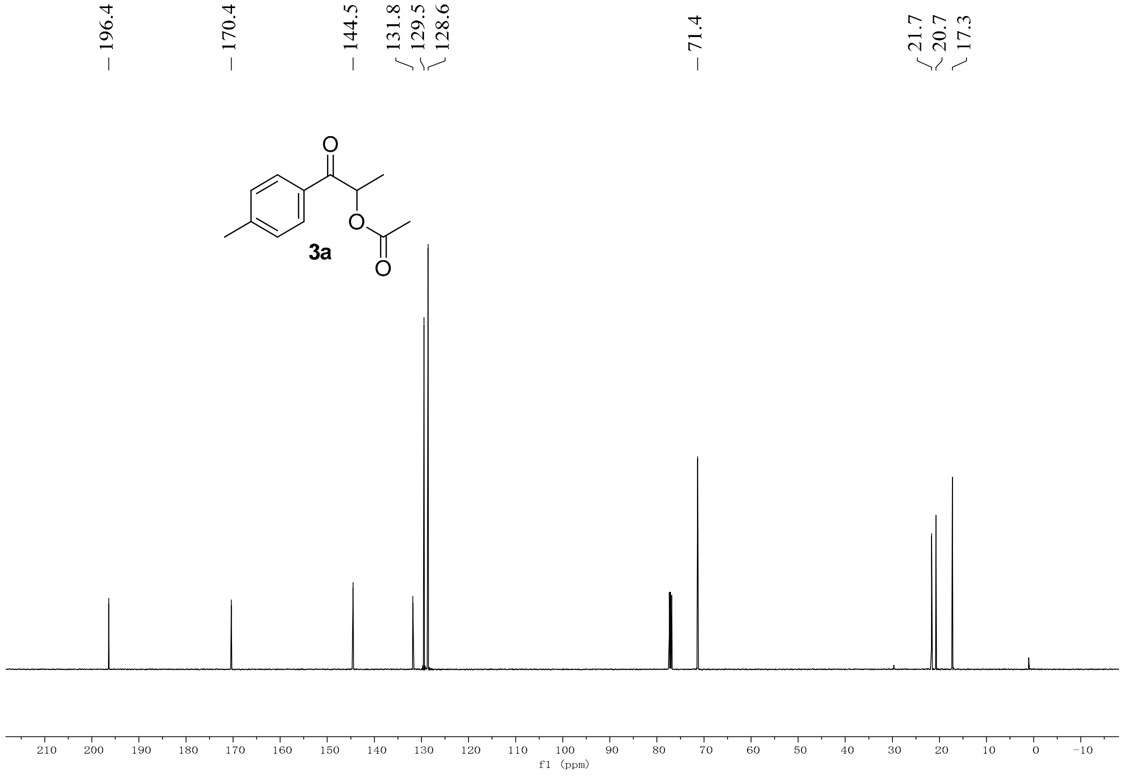

^{1}H NMR (500 MHz, $CDCl_3$) of 3c

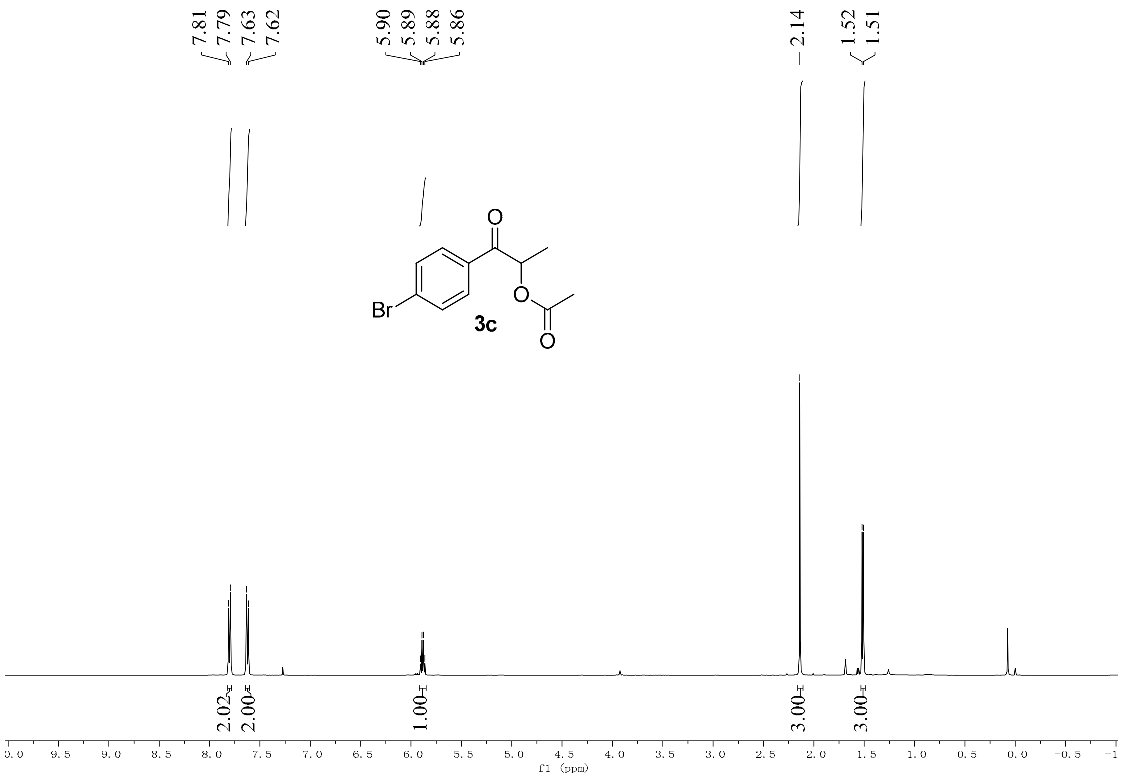

^{13}C NMR (126 MHz, $CDCl_3$) of 3c

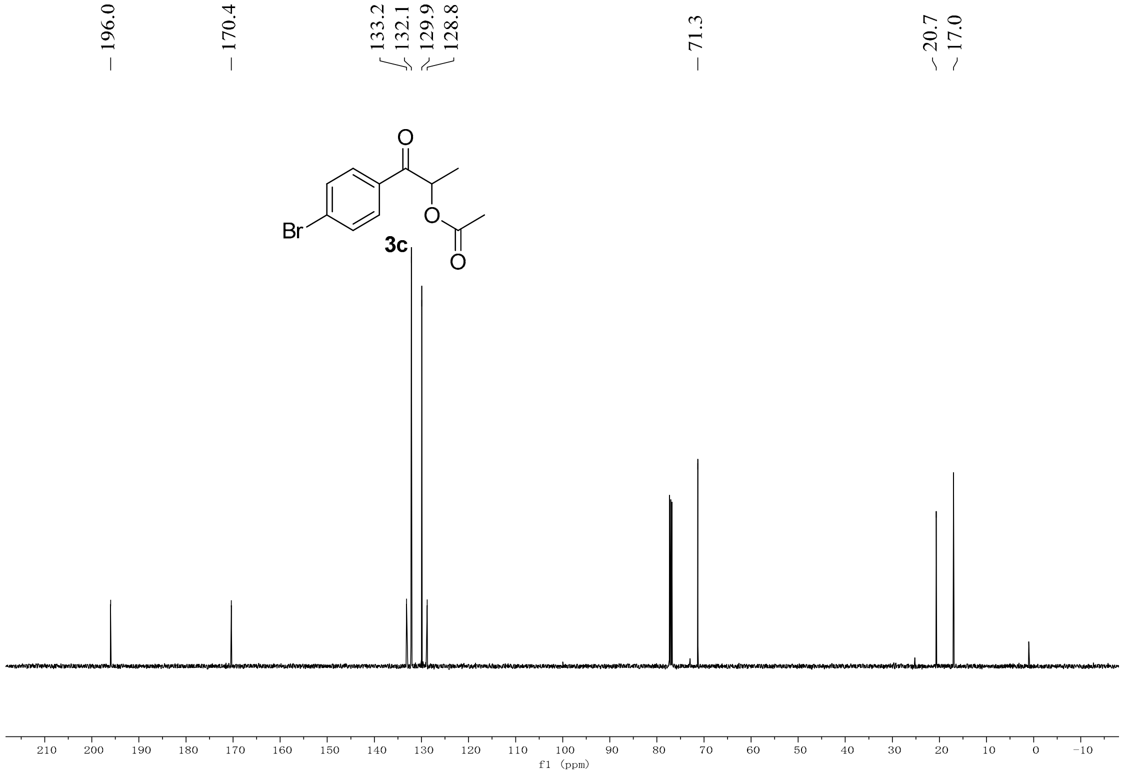

^1H NMR (500 MHz, $CDCl_3$) of 3i

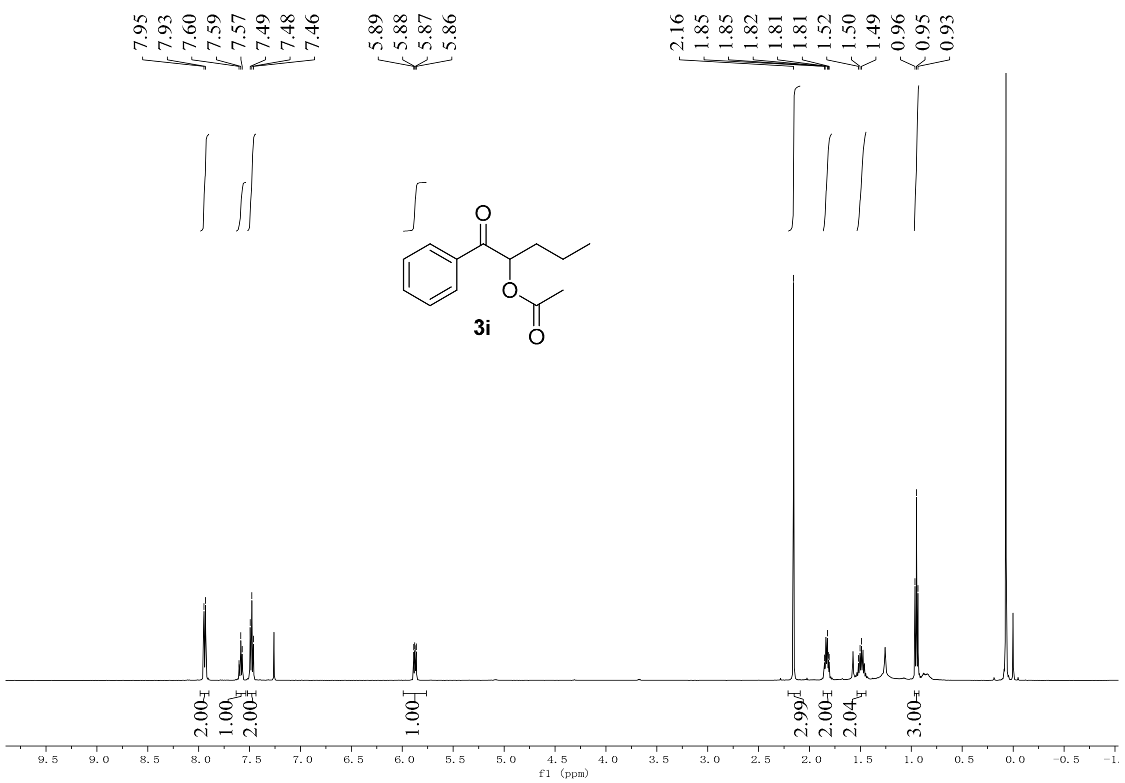

^{13}C NMR (126 MHz, $CDCl_3$) of 3i

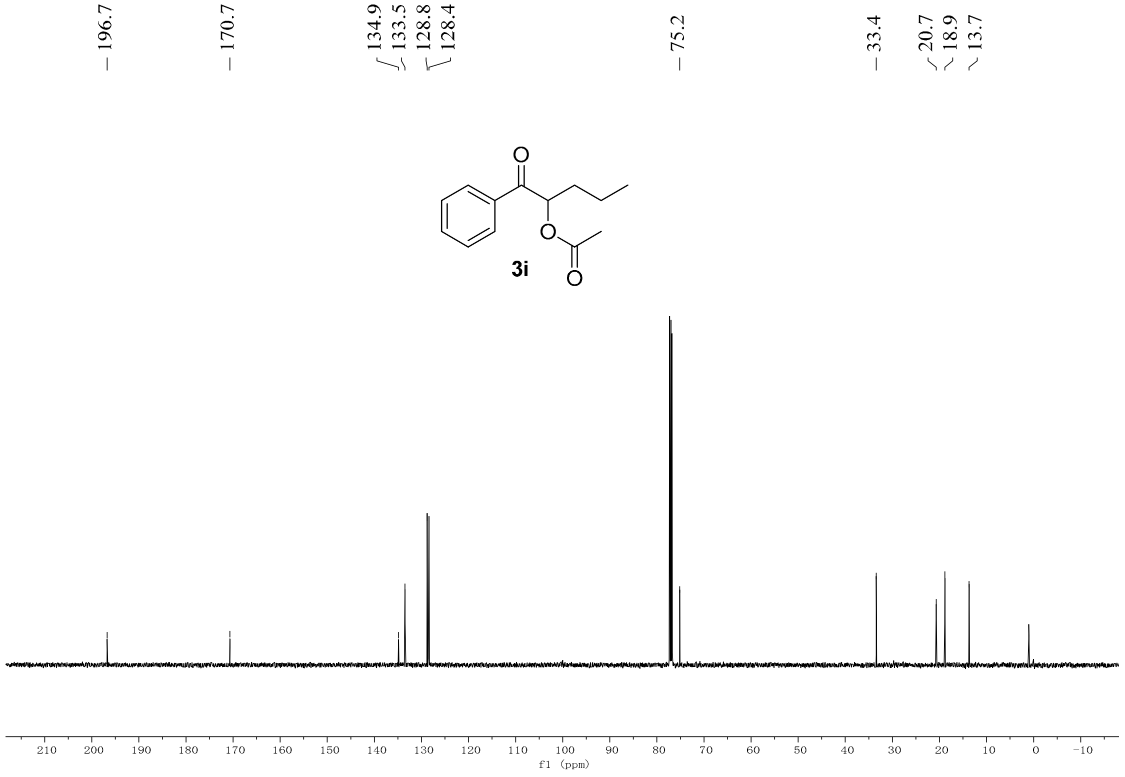

^{1}H NMR (500 MHz, $CDCl_3$) of 3k

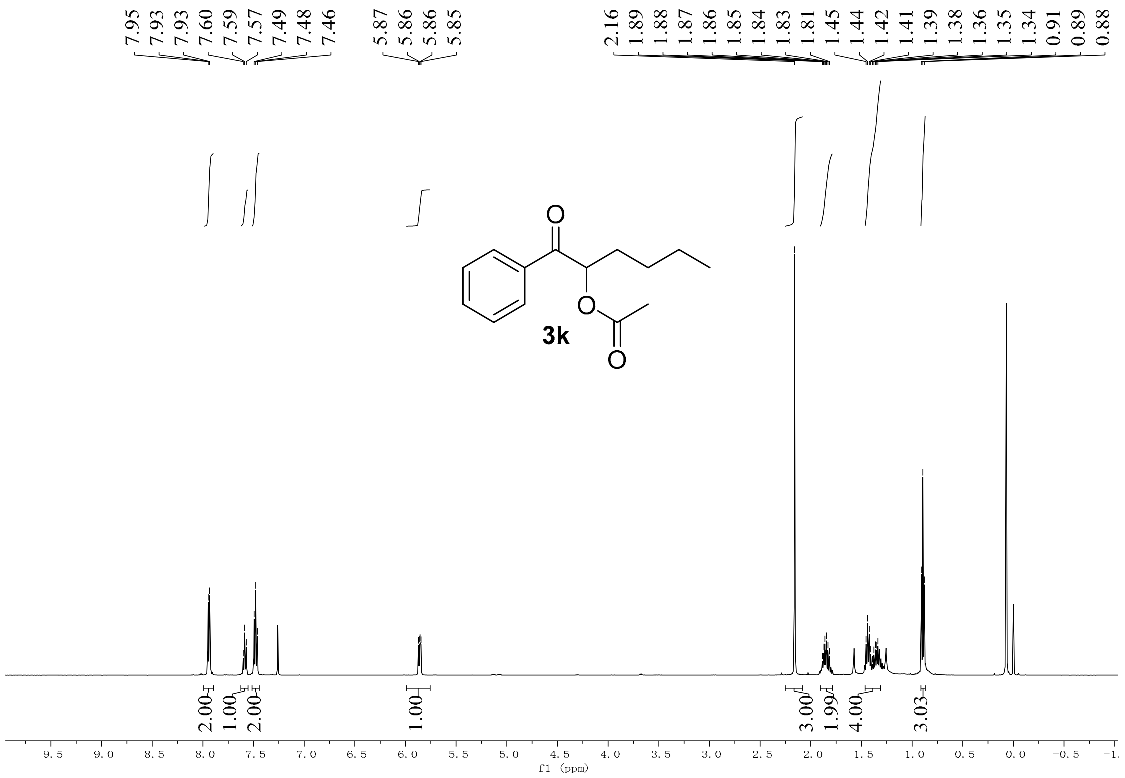

^{13}C NMR (126 MHz, $CDCl_3$) of 3k

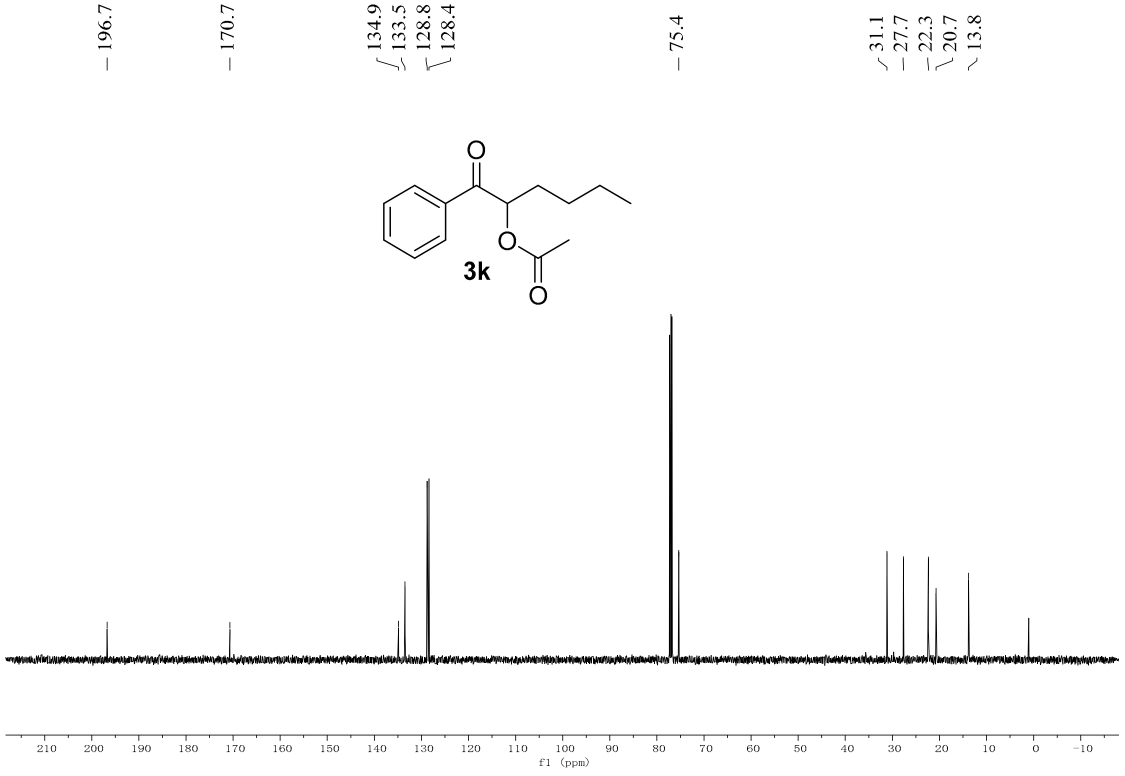

IR(KBr) of 3k

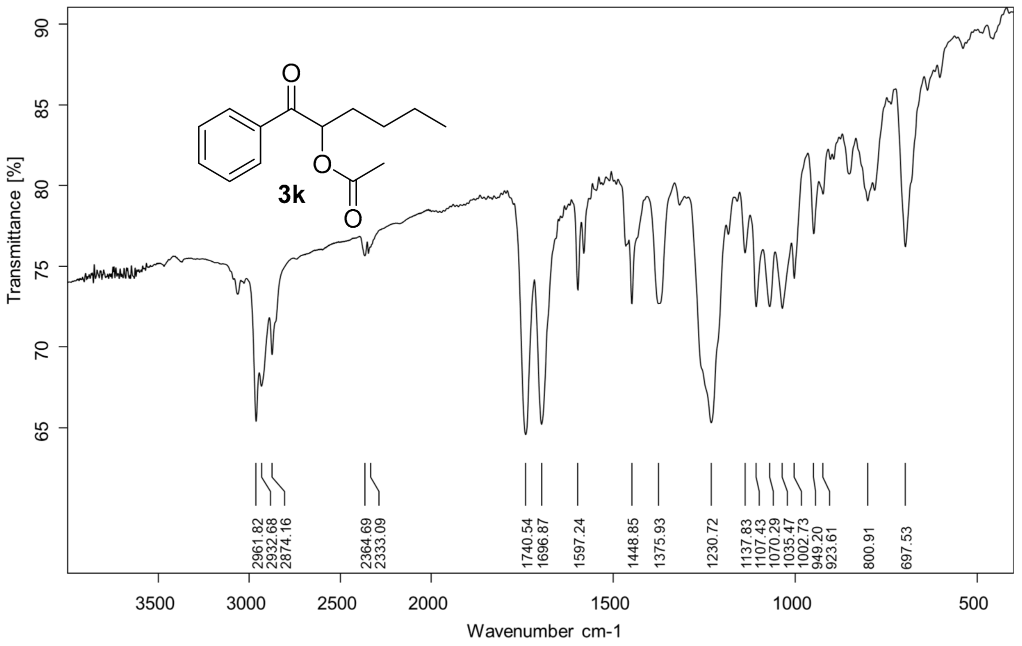

^{1}H NMR (500 MHz, $CDCl_3$) of 3m

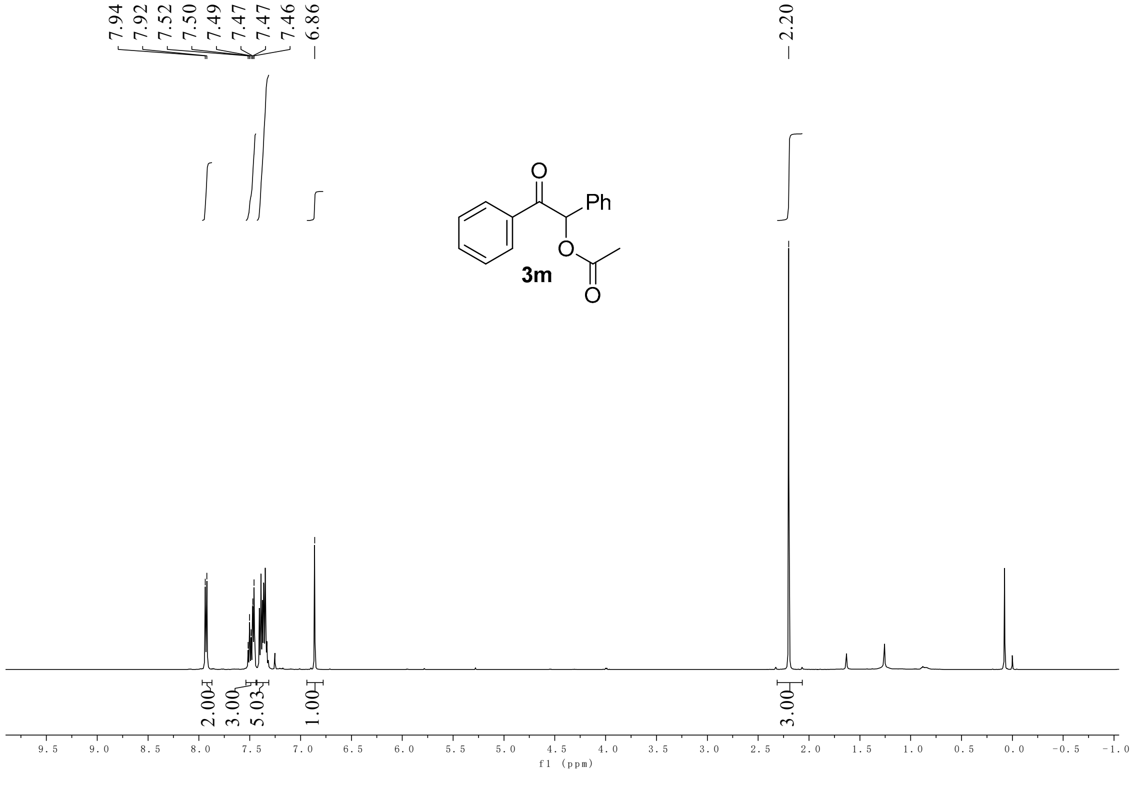

^{13}C NMR (126 MHz, $CDCl_3$) of 3m

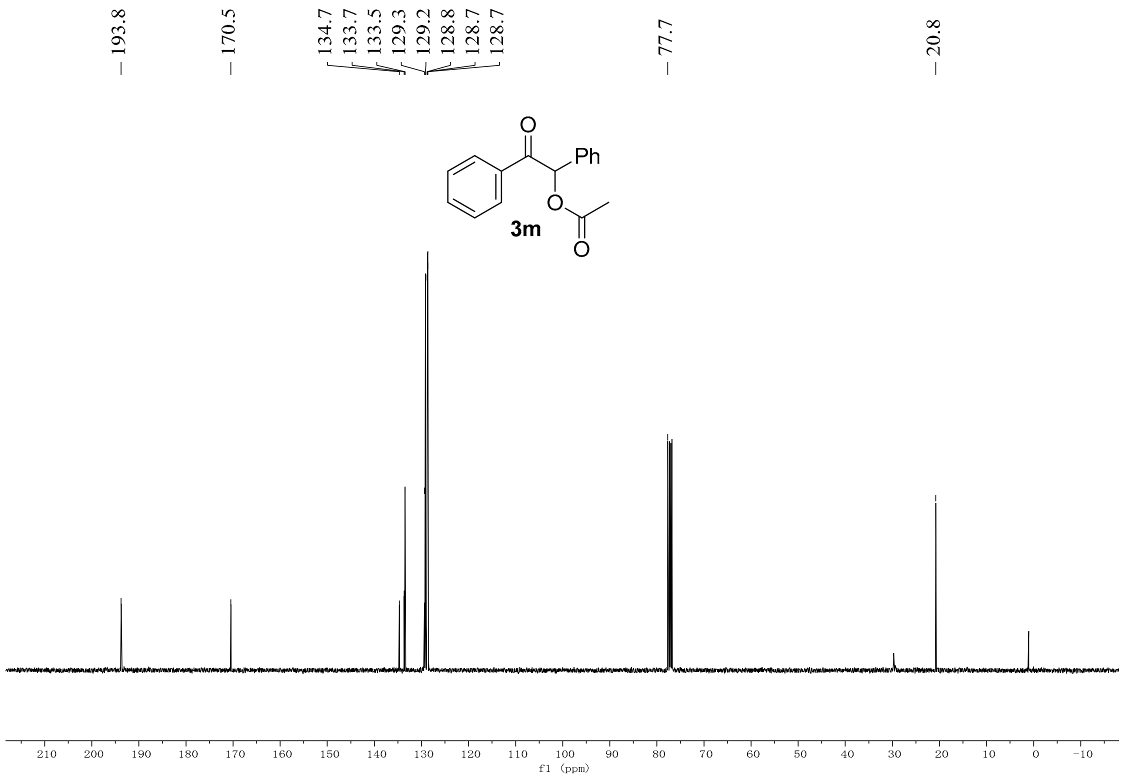

^{1}H NMR (500 MHz, CDCl$_3$) of 3q

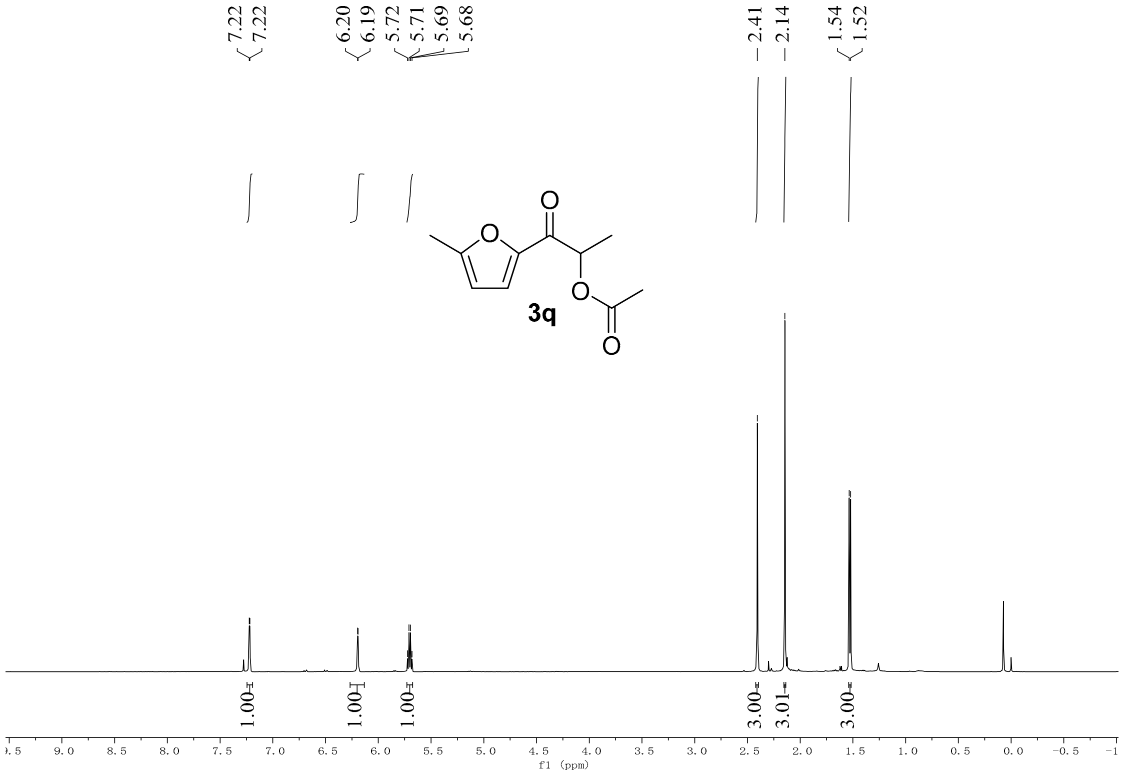

^{13}C NMR (126 MHz, CDCl$_3$) of 3q

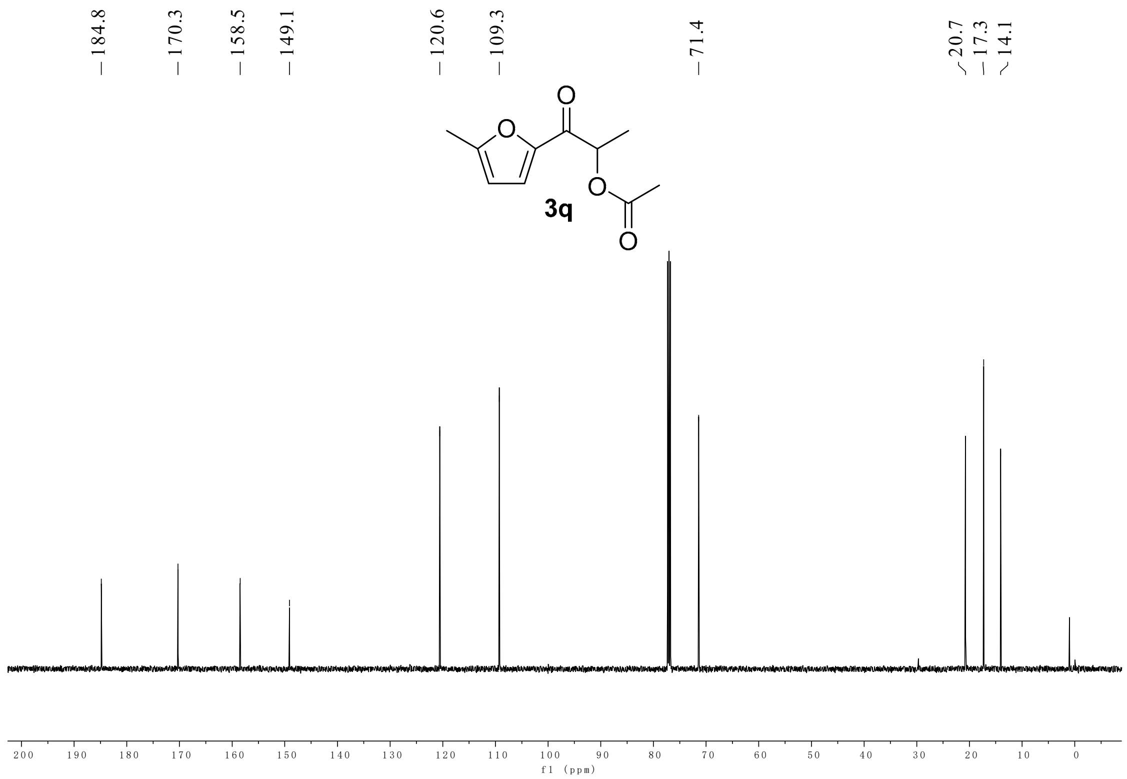

IR(KBr) of 3q

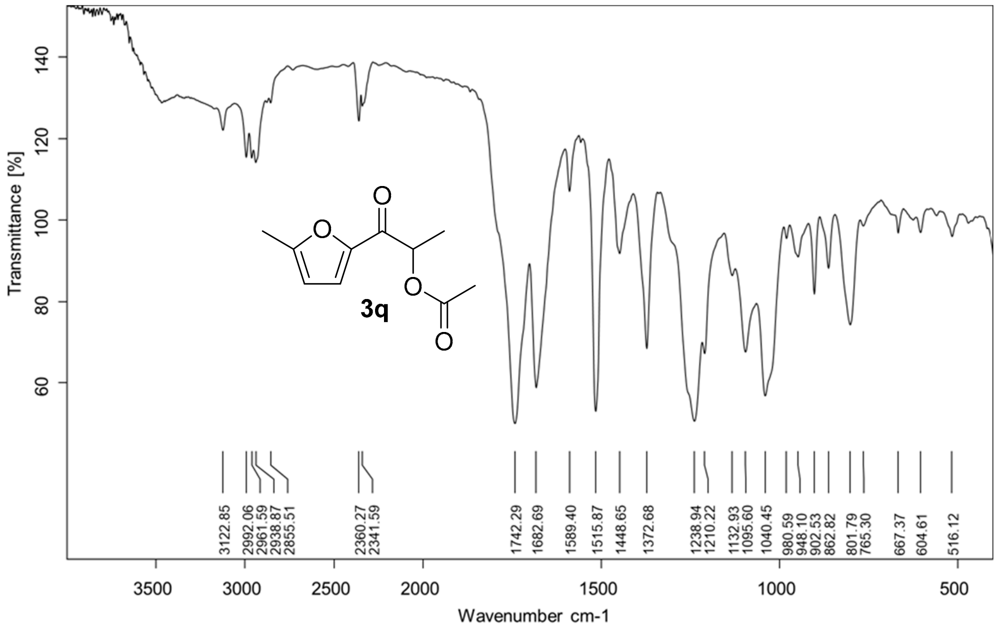

^1H NMR (500 MHz, $CDCl_3$) of 3r

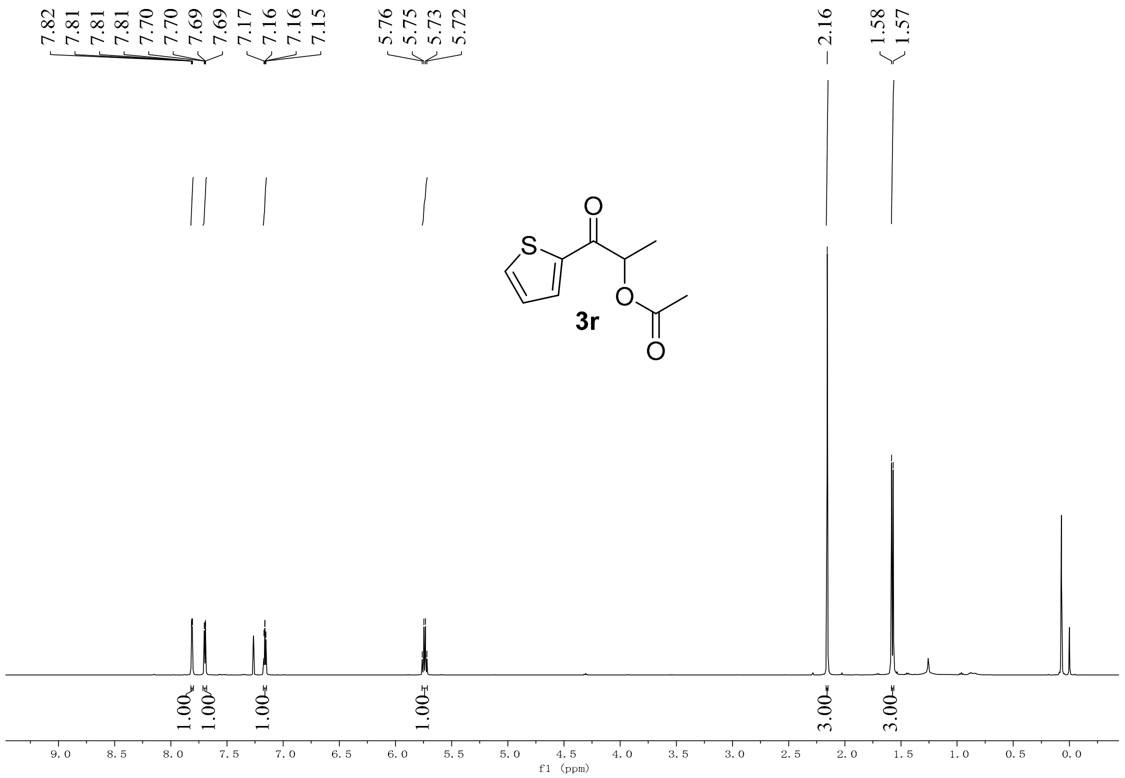

^{13}C NMR (126 MHz, $CDCl_3$) of 3r

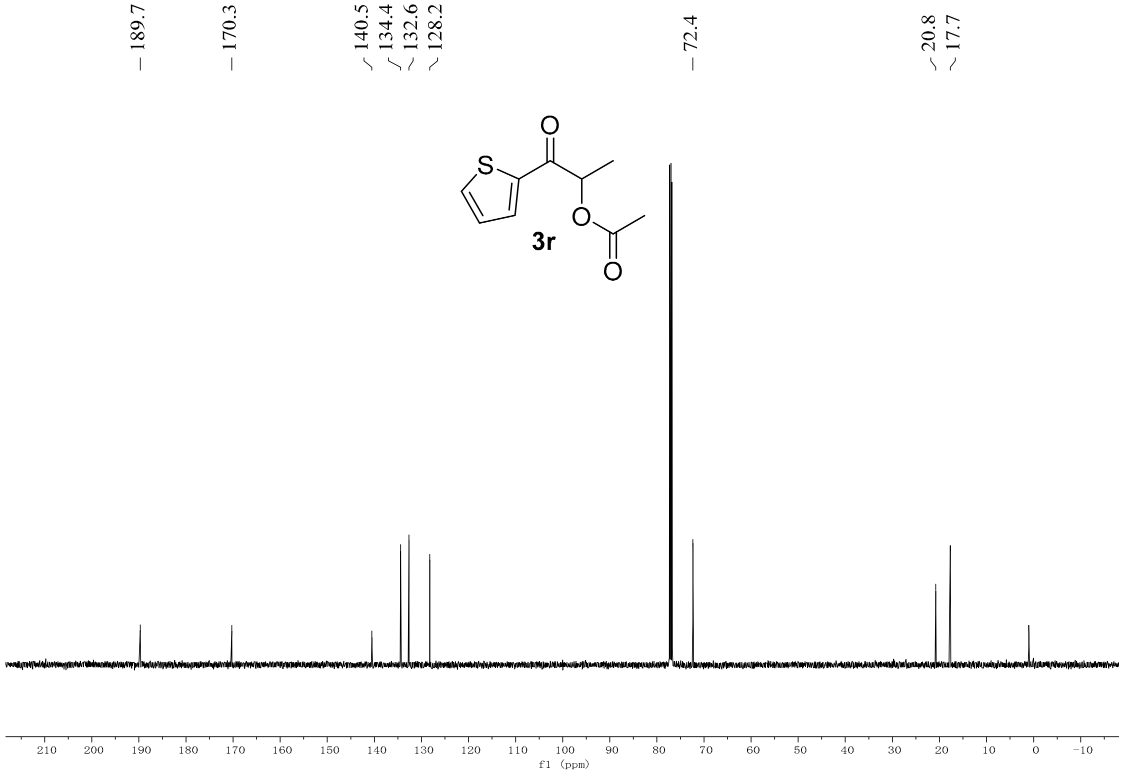

IR(KBr) of 3r

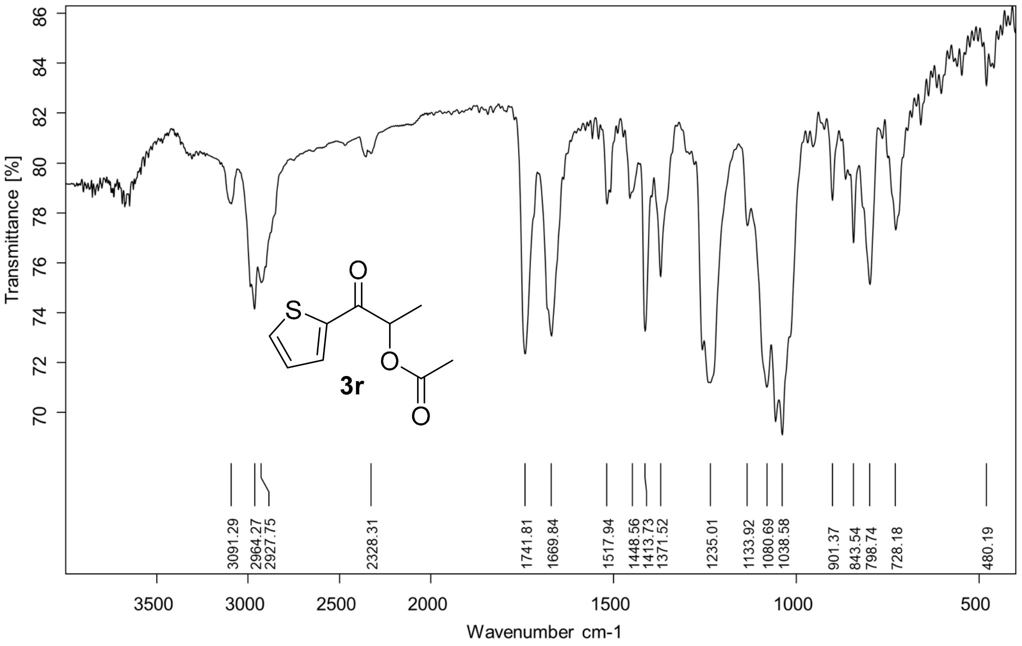

^{1}H NMR (500 MHz, $CDCl_3$) of 3u

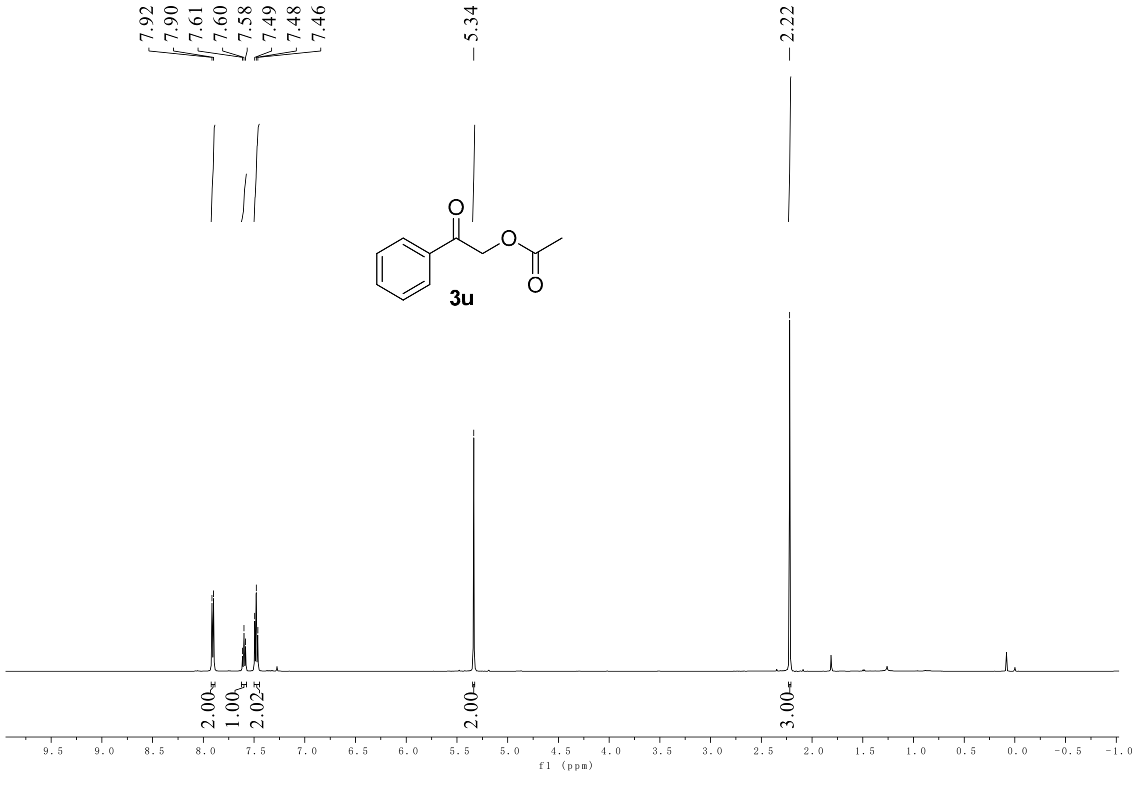

^{13}C NMR (126 MHz, $CDCl_3$) of 3u

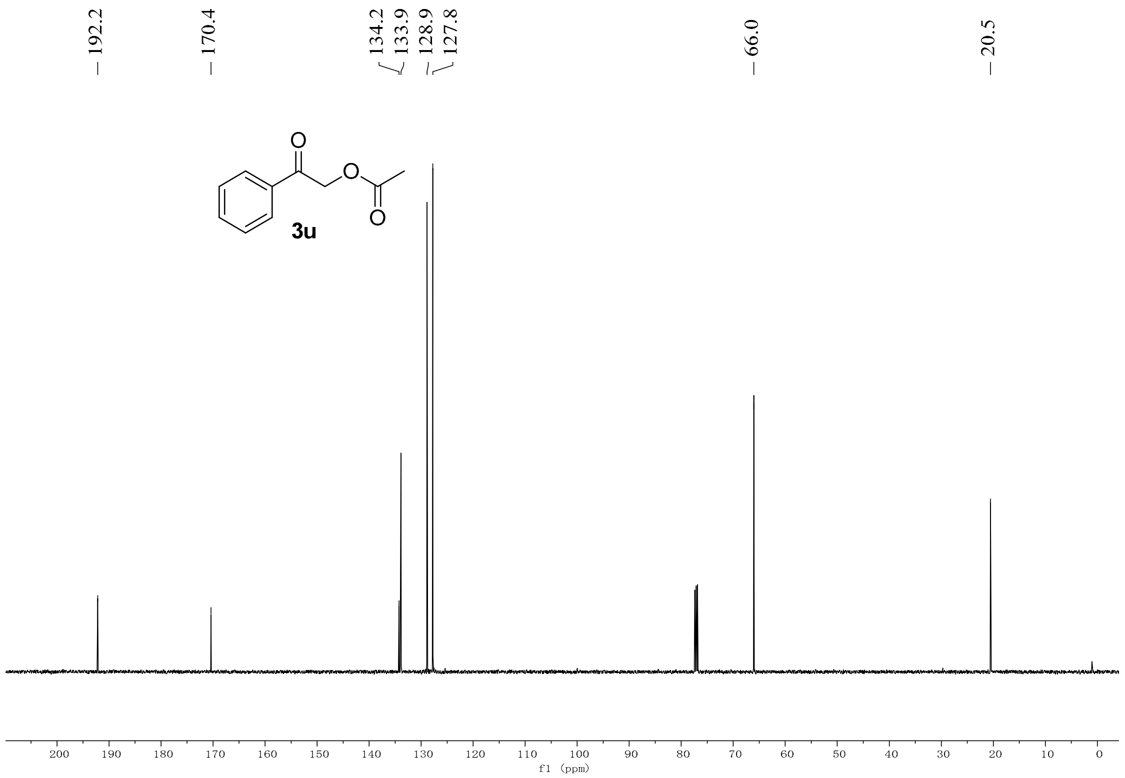

^{1}H NMR (400 MHz, $CDCl_3$) of 4a

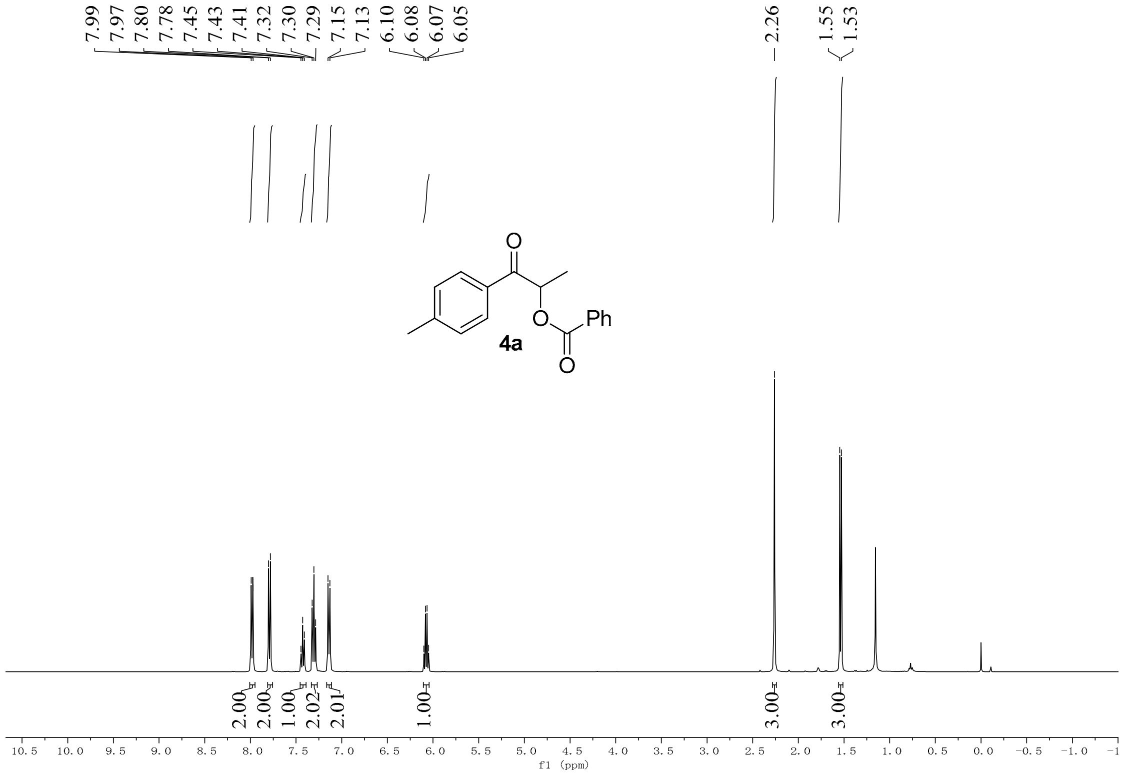

^{13}C NMR (101 MHz, $CDCl_3$) of 4a

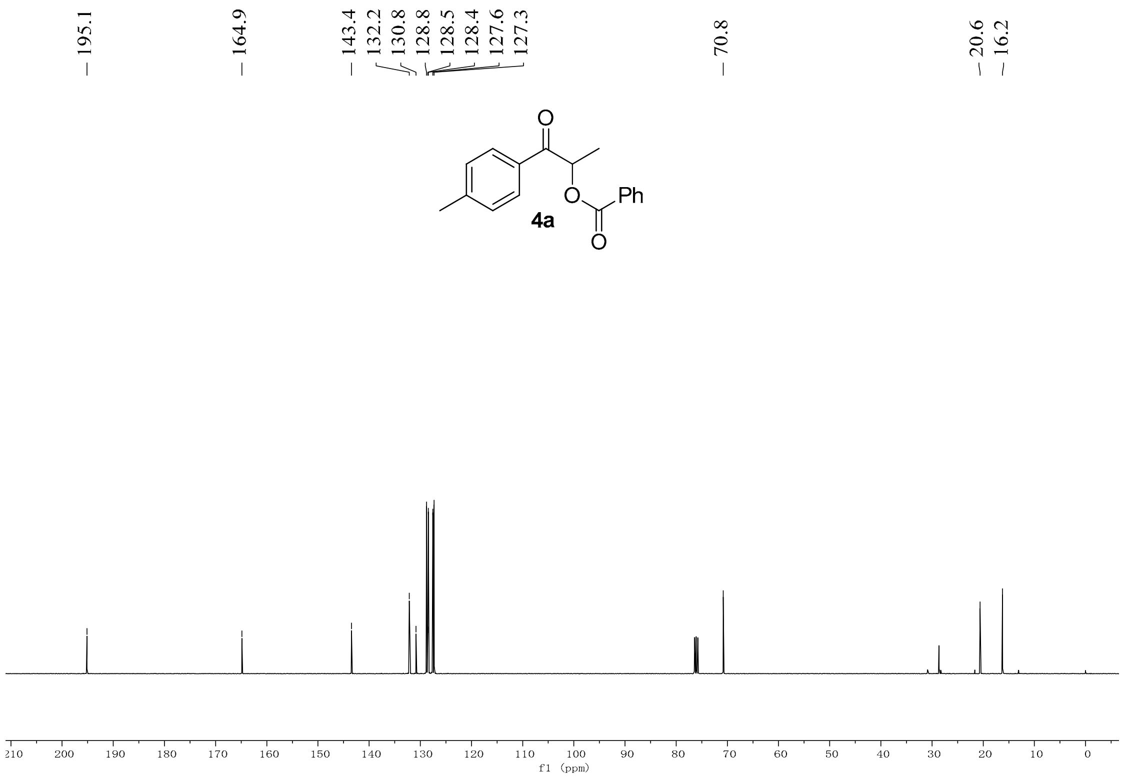

^1H NMR (400 MHz, $CDCl_3$) of 4v

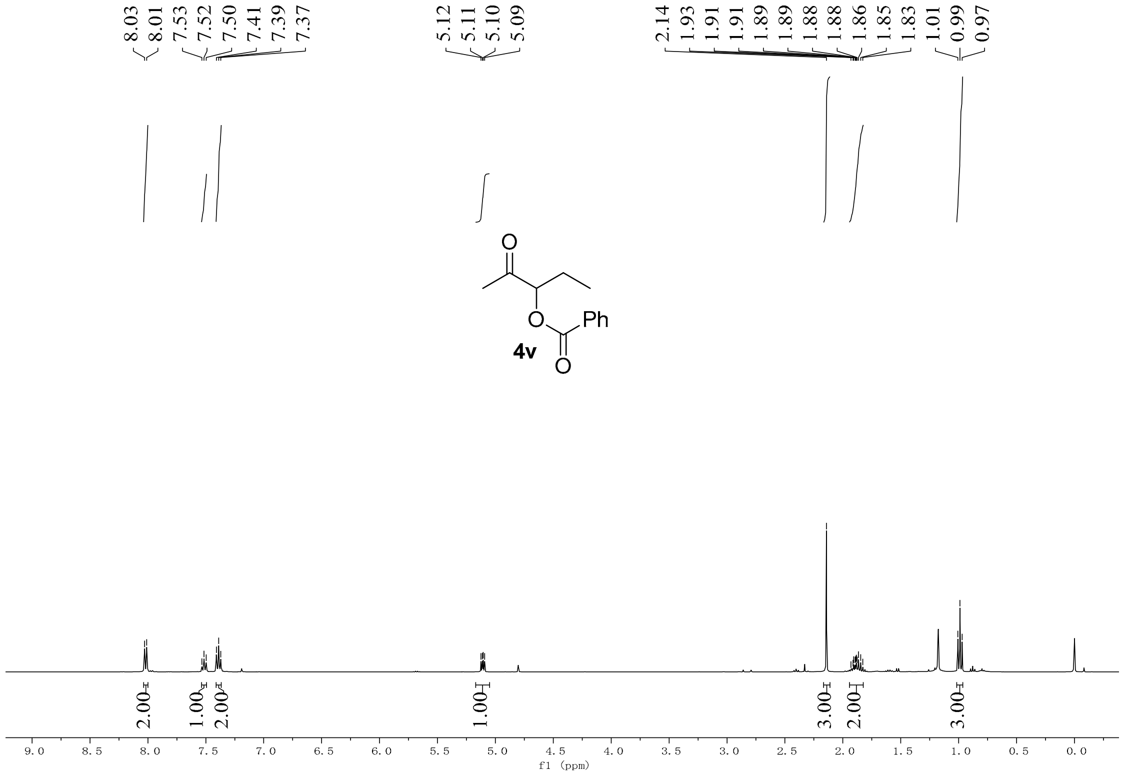

^{13}C NMR (101 MHz, $CDCl_3$) of 4v

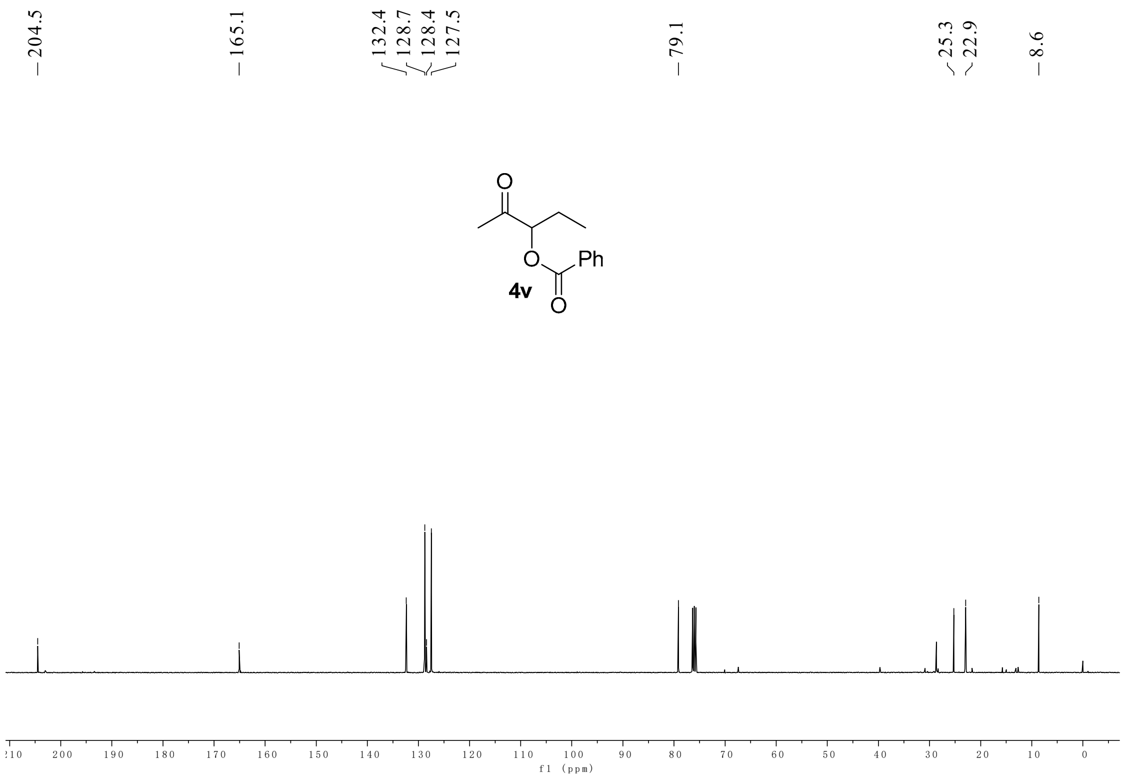

IR(KBr) of 4v

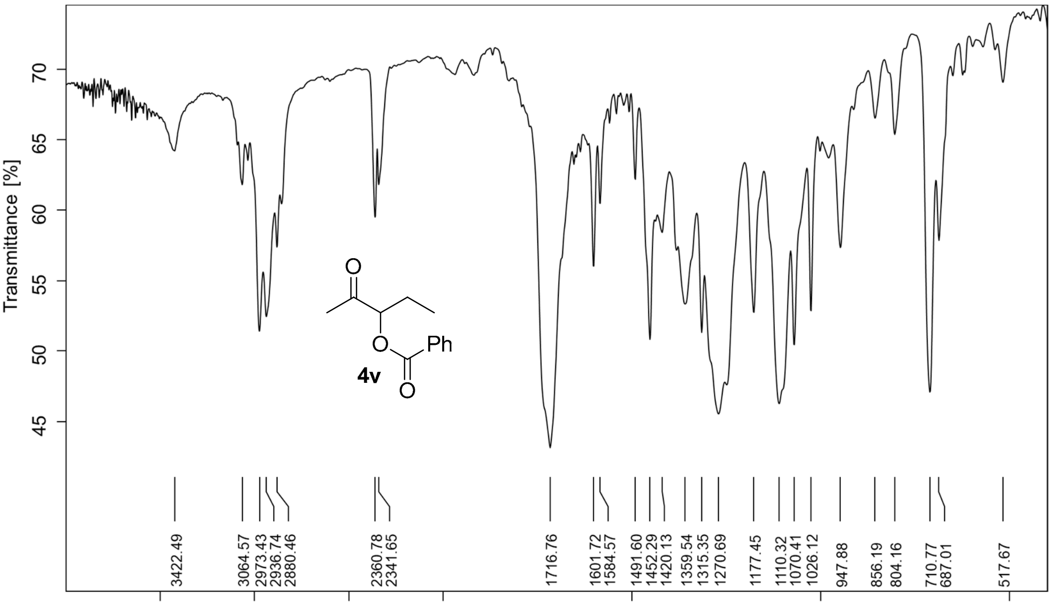

^{1}H NMR (400 MHz, $CDCl_3$) of 4w

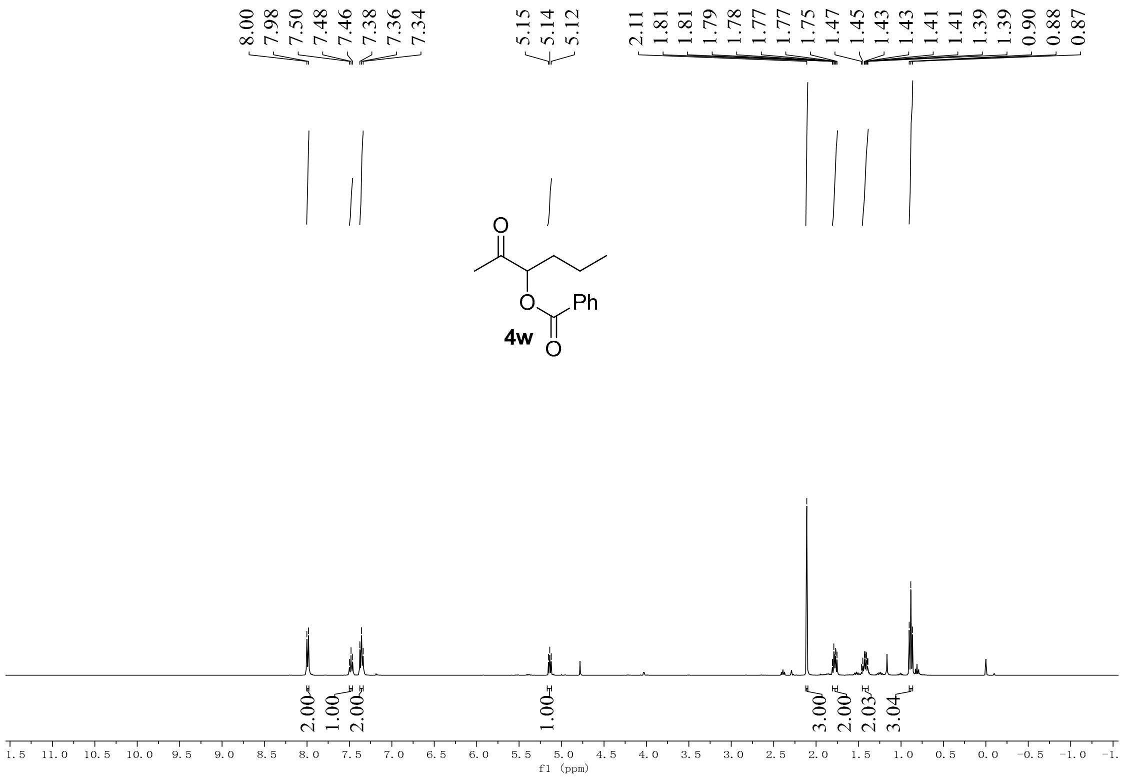

^{13}C NMR (101 MHz, $CDCl_3$) of 4w

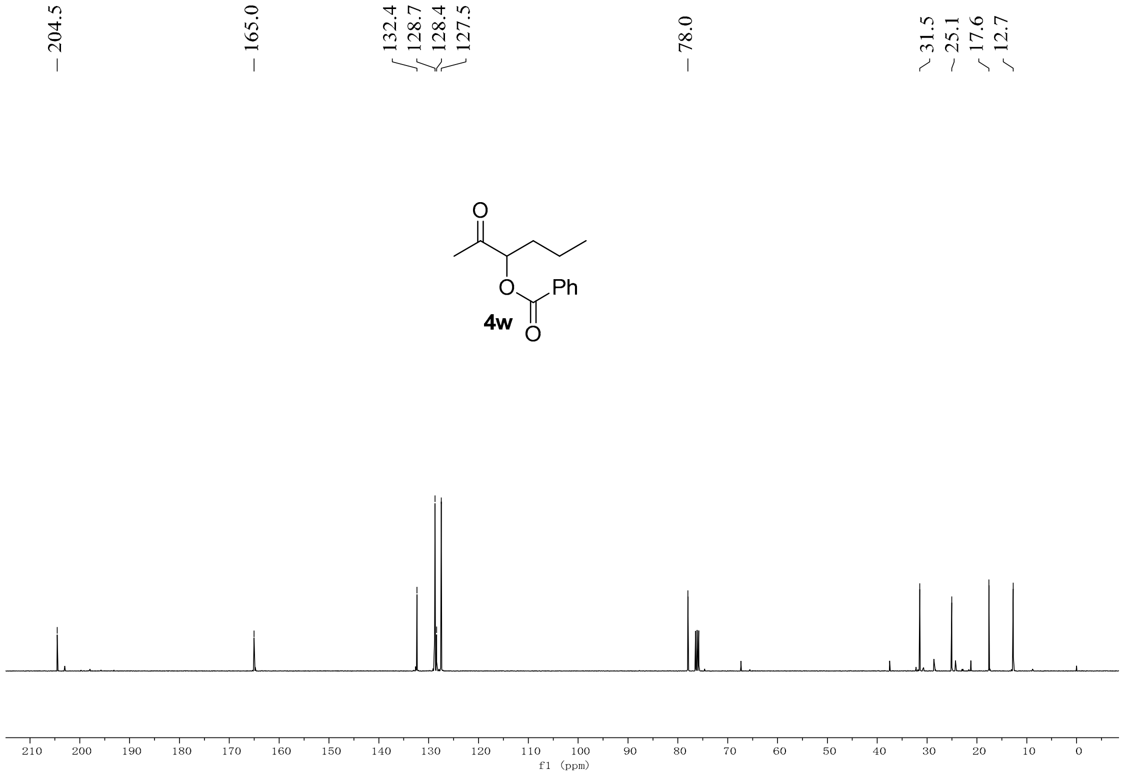

IR(KBr) of 4w

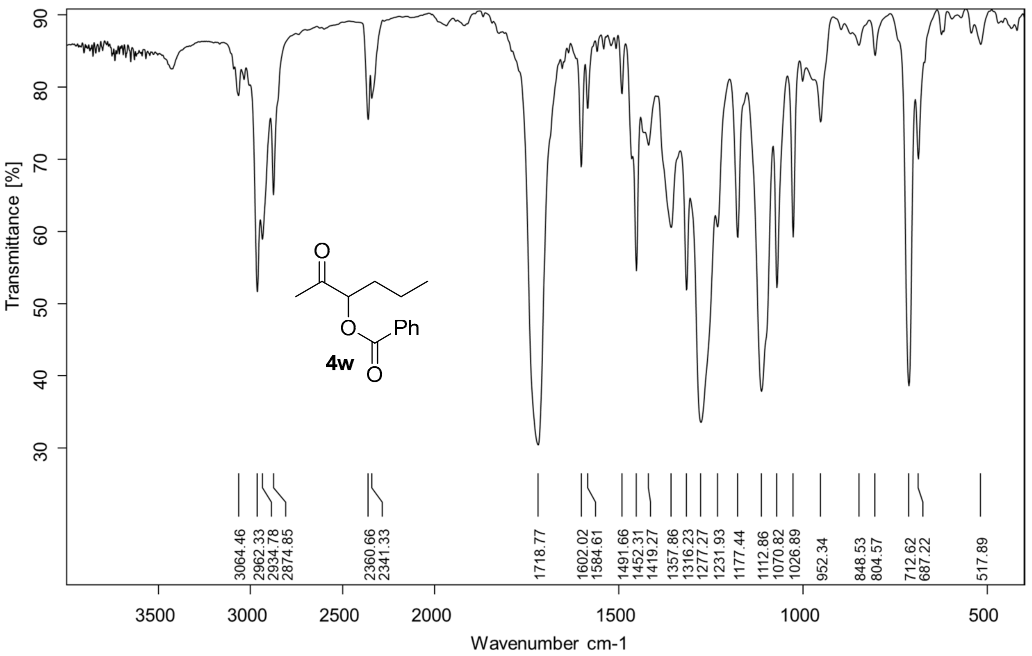

^{1}H NMR (400 MHz, CDCl$_3$) of 5a

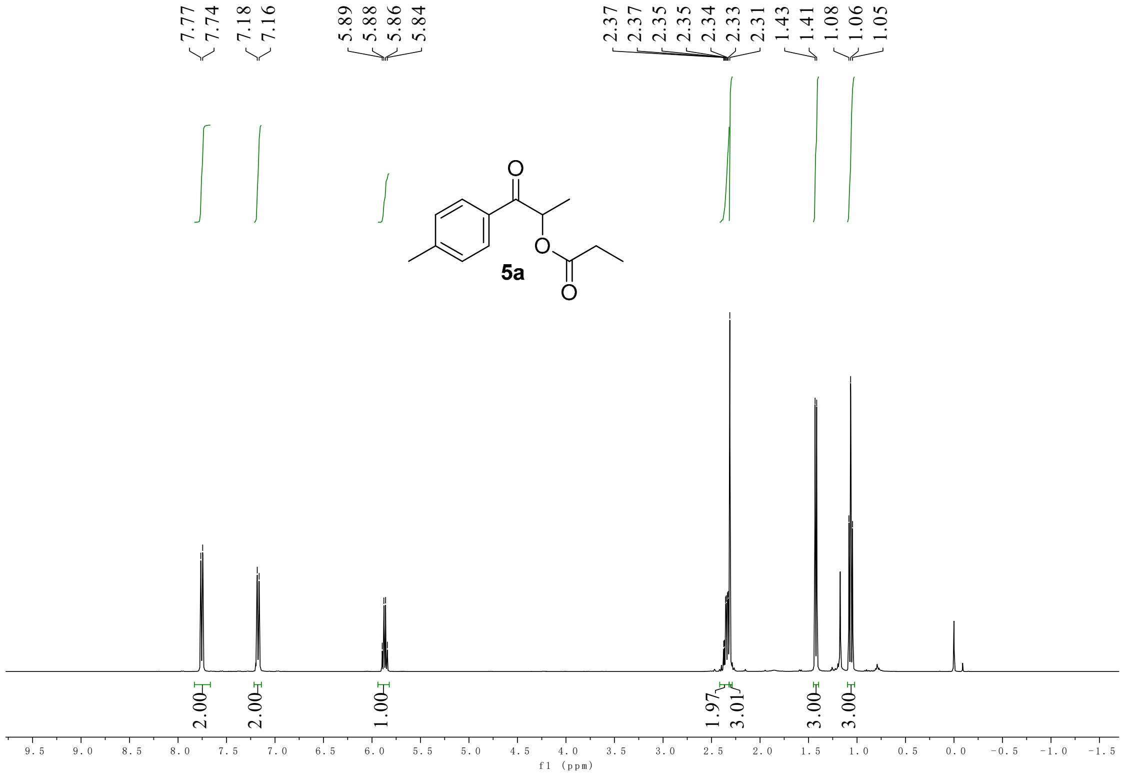

^{13}C NMR (101 MHz, CDCl$_3$) of 5a

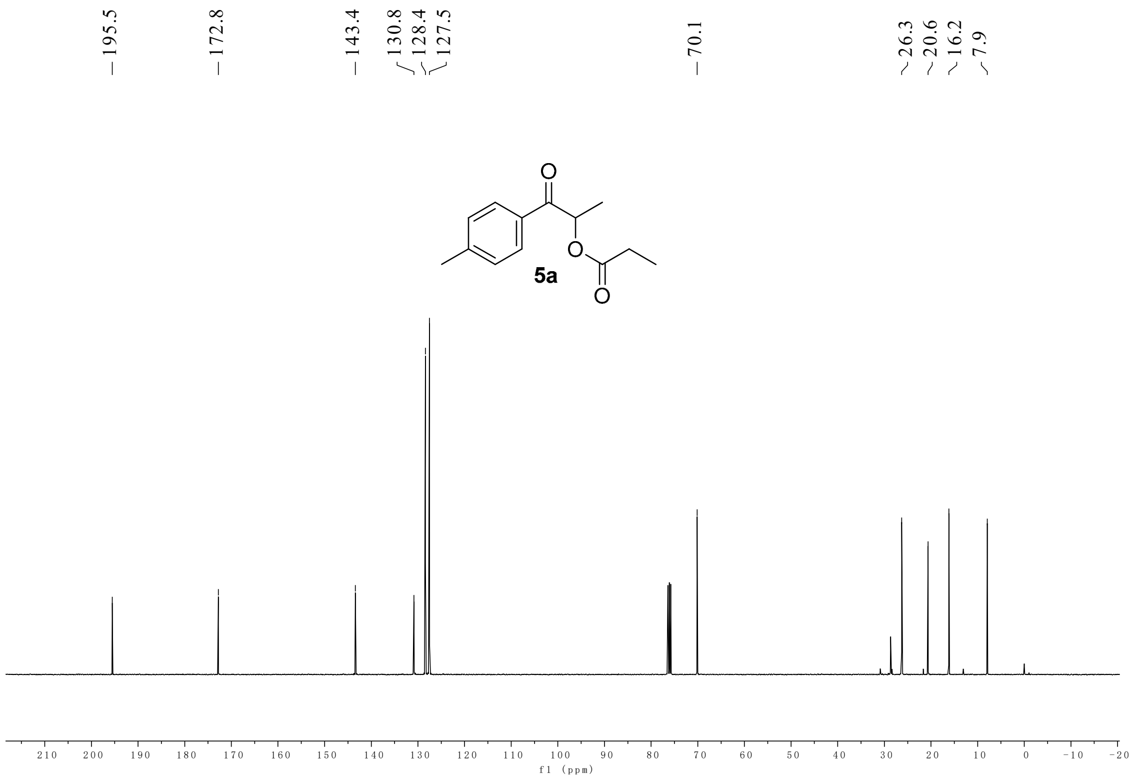

195.5
172.8
143.4
130.8
128.4
127.5
70.1
26.3
20.6
16.2
7.9
O
O
O
5a
210 200 190 180 170 160 150 140 130 120 110 100 90 80 70 60 50 40 30 20 10 0 -10 -20
f1 (ppm)

IR(KBr) of 5a

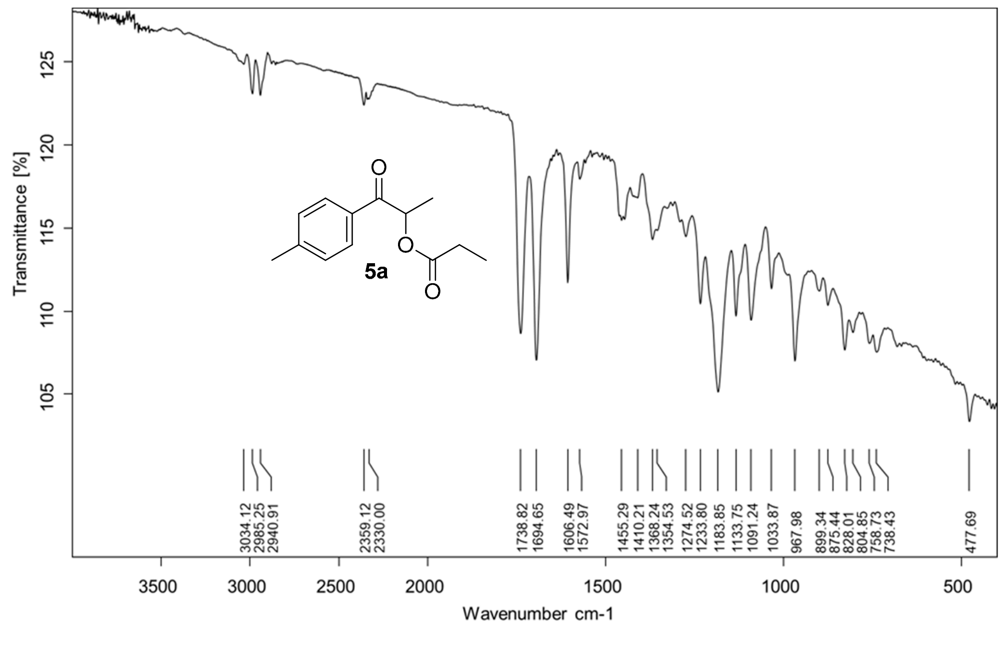

Transmittance [%]
125
120
115
110
105
O
O
O
5a
3034.12
2985.25
2940.91
2359.12
2330.00
1738.82
1694.65
1606.49
1572.97
1455.29
1410.21
1368.24
1354.53
1274.52
1233.80
1183.85
1133.75
1091.24
1033.87
967.98
899.34
875.44
828.01
804.85
758.73
738.43
477.69
3500 3000 2500 2000 1500 1000 500
Wavenumber cm-1
